# Multiscale molecular simulations for the solvation of lignin in ionic liquids

**DOI:** 10.1038/s41598-022-25372-2

**Published:** 2023-01-06

**Authors:** Mood Mohan, Blake A. Simmons, Kenneth L. Sale, Seema Singh

**Affiliations:** 1 Deconstruction Division, Joint BioEnergy Institute, 5885 Hollis Street, Emeryville, CA 94608 USA; 2 Bioresource and Environmental Security Department, Sandia National Laboratories, 7011 East Avenue, Livermore, CA 94551 USA; 3 Biological Systems and Engineering Division, Lawrence Berkeley National Laboratory, 1 Cyclotron Road, Berkeley, CA 94720 USA; 4 Department of Computational Biology and Biophysics, Sandia National Laboratories, 7011 East Avenue, Livermore, CA 94551 USA

**Keywords:** Renewable energy, Ionic liquids

## Abstract

Lignin, the second most abundant biopolymer found in nature, has emerged as a potential source of sustainable fuels, chemicals, and materials. Finding suitable solvents, as well as technologies for efficient and affordable lignin dissolution and depolymerization, are major obstacles in the conversion of lignin to value-added products. Certain ionic liquids (ILs) are capable of dissolving and depolymerizing lignin but designing and developing an effective IL for lignin dissolution remains quite challenging. To address this issue, the COnductor-like Screening MOdel for Real Solvents (COSMO-RS) model was used to screen 5670 ILs by computing logarithmic activity coefficients (*ln*(*γ*)) and excess enthalpies (*H*^*E*^) of lignin, respectively. Based on the COSMO-RS computed thermodynamic properties (*ln*(*γ*) and *H*^*E*^) of lignin, anions such as acetate, methyl carbonate, octanoate, glycinate, alaninate, and lysinate in combination with cations like tetraalkylammonium, tetraalkylphosphonium, and pyridinium are predicted to be suitable solvents for lignin dissolution. The dissolution properties such as interaction energy between anion and cation, viscosity, Hansen solubility parameters, dissociation constants, and Kamlet–Taft parameters of selected ILs were evaluated to assess their propensity for lignin dissolution. Furthermore, molecular dynamics (MD) simulations were performed to understand the structural and dynamic properties of tetrabutylammonium [TBA]^+^-based ILs and lignin mixtures and to shed light on the mechanisms involved in lignin dissolution. MD simulation results suggested [TBA]^+^-based ILs have the potential to dissolve lignin because of their higher contact probability and interaction energies with lignin when compared to cholinium lysinate.

## Introduction

In the race for sustainable energy, lignocellulosic biomass has been estimated to be able to provide 20% of the world's energy demand by 2050^1^. A total of 170 billion metric tons of lignocellulosic biomass are produced each year around the world^2^. In order to realize the full potential of lignocellulosic biomass, the lignin (roughly 20–30 wt% of the initial biomass composition) has yet to be efficiently utilized at an industrial scale^3–5^. Dissolution and depolymerization of lignin during lignocellulosic biomass deconstruction is a critical step in the production of biofuels and lignin-based bioproducts^6–8^. Not only does lignin prevent enzymatic biomass degradation^9,10^, but to make biorefineries economically viable, it must be used to produce value-added bioproducts^11^. After lignin is removed, cellulose and hemicellulose can be further transformed into biofuel precursors^12–14^, while the dissolved lignin can serve as a valuable precursor for carbon fibers^15^, colloids^16^, thermoplastics^17,18^, aromatic chemicals^19^, lignin-based nanomaterials^4,20^, commercial adhesives^21^, and more^22^.

The aromatic backbone of lignin is composed of three main phenylpropanoid units, p-hydroxy phenol (H), guaiacyl (G), and syringyl (S)^7,23^. These phenylpropanoid monomers are linked together by ether (C–O–C: β‒O‒4, α‒O‒4, and 4‒O‒5) and C–C (β‒β, β‒5, β‒1, and 5‒5) bonds formed during biosynthesis^23–25^. Lignin is highly resistant to enzymatic breakdown because of its heterogeneity, hydrophobicity, and cross-linked structure^26^. Additionally, lignin contains an abundant number of intramolecular hydrogen bonds and π–π interactions between the rigid aromatic rings, resulting in a compact three-dimensional H-bond network, adding to its recalcitrance to enzymatic depolymerization. Lignin is also very insoluble in water and most common organic solvents, thus limiting its potential use for high-value chemical applications. As a result, sustainable biorefineries require a solvent that is capable of removing lignin and effectively rendering biomass amenable to deconstruction into upgradable fragments.

Certain types of ionic liquids (ILs) have been demonstrated to be promising biomass solvents over the past few decades and have opened up new opportunities for the dissolution of cellulose and biomass fractionation^13,27–31^. Ionic liquids are molten salts with a melting temperature of less than 100 °C and have unique properties including low vapor pressure, nonflammability, non-toxic, high chemical and thermal stability, and high solubility^32–34^. The most fascinating property of ILs is their diversity in chemical structures resulting from the large number of possible combinations of anions and cations. In the last few years, the dissolution of lignin in ILs has received much attention. Pu et al.^35^ investigated the influence of imidazolium-based ILs on lignin dissolution and found that ILs containing methyl sulfate and trifluorosulfonate anions were effective for dissolving softwood Kraft lignin. Hou et al.^36^ measured the solubility of Kraft lignin in a series of carboxylate anion-based ILs with cholinium cation. It has been reported that as the alkyl chain length of the anion increased from formate to octanoate, the ability of the IL to solubilize lignin also increased. In addition, it also has been reported that the anions with basic group(s) (e.g.: lysinate [Lys]^−^) are effective ILs for biomass delignification and significantly enhanced the enzymatic hydrolysis yields of glucose and xylose. Other studies claimed that the solubility of lignin is influenced not only by anion properties, but also by the properties of the cation. According to Hart et al.^37^, the cation has a considerably more subtle effect on lignin solubility, with planar cations being the most effective. Further, Wang et al.^38^ studied the influence of alkyl chain length of cations on the solubility of lignin in dialkylimidazolium-based ILs. As the alkyl chain length of cations increased from C4 to C8, the solubility of lignin decreased.

Given the potential capabilities of these ILs, it is critical to understand the thermodynamic properties (e.g., solubility, viscosity, interaction between anions and cations, Kamlet–Taft parameters etc.) of neat ILs and their mixtures with lignin. It is, however, impractical to investigate each and every possible IL anion-cation pair experimentally because of the large number of potential combinations of cations and anions exist. Therefore, it is necessary to develop theoretical methodologies that are reliable to screen the enormous number of ILs for lignin dissolution before conducting experiments. Several predictive techniques have recently been developed and adapted for modeling the thermodynamic properties of systems containing ILs, such as the perturbed-chain statistical associating fluid theory (PC-SAFT) method^39^, group contribution methods^40^, quantitative structure–property relationships (QSPR)^41^, Monte Carlo (MC) molecular simulations^42^, molecular dynamics (MD) simulations^43^, and COnductor-like Screening MOdel for Real Solvents (COSMO-RS)^44,45^. On the other hand, a potentially useful approach is to develop machine learning (ML) techniques. Using the ML models, the designing of solvents and catalysts for lignin dissolution and depolymerization are possible with higher accuracy. Recently, Ding et al.^46^ developed ML models for screening efficient ionic liquid for cleavage of the lignin ether (*β*‒O‒4) linkage by predicting the bond dissociation energies (BDE) of lignin in ILs. Smuga-Kogut et al*.*^47,48^ have developed the ML models for predicting bioethanol yields from lignocellulosic biomass with the use of ionic liquid pretreatments. These models are developed with a small set of data points; however, to develop a robust ML model for lignin and biomass conversion, a vast number of databases are needed. Therefore, among these predictive techniques, the COSMO-RS model is a powerful tool for rapidly screening large numbers of ILs and has been widely used for the screening of ILs for different applications^27,49–51^. The ability to screen ILs using predictive methods that only require a minimal number of input parameters would result in significant savings in time, cost, and effort in laboratory studies.

Fundamental understanding and development of a suitable solvent system for lignin dissolution by computational and experimental methods have immense potential, and implementation of the COSMO-RS model in screening of ILs for cellulose, hemicellulose, and lignin dissolution has been successfully demonstrated^27,51–54^. For the dissolution of lignin, Balaji et al*.*^52^ and Casas et al^53,55^ screened hundreds of ILs by predicting the Hildebrand solubility parameter and thermodynamic properties (excess enthalpy and activity coefficient) using the COSMO-RS model. They reported that closer solubility parameter values, lower activity coefficient and exothermic behavior of excess enthalpy are beneficial for higher dissolution of lignin. However, in both studies, only lignin monomer structures were used as model compounds for their computations. Recently, Yu et al.^56^ designed 19 lignin model components (monomers, dimers, and trimers) and computationally screened 3886 ILs using the COSMO-RS calculations. Lignin's monomeric and dimer structures do not accurately depict the lignin molecule because the lack of many different linkages present in lignin. In fact, not only the ILs used for lignin dissolution are limited at present, but also the generalized lignin model structure is not fully explored for molecular simulations. In such a case, no general guidelines can be summarized and used to anticipate the most appropriate solvent to dissolve lignin, which hinders identification and optimization of process for conversion of lignin to and high-value products. Therefore, systematic screening of structurally diverse ILs is essential for developing a comprehensive theoretical understanding of lignin solubilization in IL-based solvent systems and for developing optimal solvents that will facilitate lignin valorization.

For lignin dissolution, ionic liquids have shown some unique properties and have gained much attention in research. Much development work remains to be done, however, as the technical challenges shift from fundamental scientific understanding to process-oriented engineering exploitation, the research priorities are likely expected to evolve. Even though the market for ionic liquids wasn’t as big as expected in the last decade, there is an increasing number of encouraging trends. The most impressive aspect is the 57 ionic liquid applications that have been successfully implemented to this day, which represents a significant leap when compared to the 13 applications that were known to exist in 2008^57^. There will be an optimistic future for ionic liquids, and “this is very much the end of the beginning regarding ionic liquid technologies, and not the beginning of the end”. According to a survey that was conducted by Roland Kalb^57^ at Proionic, which includes 25 academic and industrial leaders, it was determined that “the field of ionic liquids will soon reach maturity and enter the megatrend mass markets”.

Thus, the present study attempts to use multiscale molecular simulation strategies to design and develop the fundamental knowledge required to develop robust IL solvent systems for lignin. The following goals are intended to address: (1) develop a representative lignin model structure for the molecular simulations and screen thousands of ILs for lignin dissolution using the COSMO-RS model. (2) determine the parameters from the COSMO-RS model and quantum chemistry (QC) calculations that influence lignin dissolution. Key parameters such as interaction energy between anion and cation of the IL, viscosity, Hansen solubility parameters, dissociation constants, and Kamlet–Taft parameters were evaluated to assess relationships between IL properties and lignin dissolution. Finally, (3) analyze the structural and dynamic properties (radius of gyration, solvent accessible surface area, hydrogen bonds, radial distribution functions, and mass fractal dimension factor) of lignin in IL-lignin mixtures using molecular dynamics (MD) simulations to shed light on the mechanisms involved in lignin dissolution.

## Results and discussions

### Benchmarking study

The COSMO-RS model is a powerful computational approach to calculating thermodynamic properties and screening solvents for polymer dissolution. Only structural information of the solvent and solute is typically required for the COSMO-RS to predict the solubility and other thermodynamic properties. It should be worthwhile to mention that the COSMO-RS predicted thermodynamic properties such as logarithmic activity coefficient and excess enthalpies are critical parameters in determining the ability of a solvent on the solute’s dissolution. The activity coefficient and excess enthalpies are related to the solubility and interaction between unlike species (e.g., lignin and ILs), respectively. Solute with lower logarithmic activity coefficient *ln*(*γ*) and excess enthalpies (*H*^*E*^) in the solvents indicate that solvent has a stronger tendency to interact and solubilize the solute. In our earlier work on lignocellulosic biomass, we showed the applicability of COSMO-RS for calculating activity coefficients (ϒ) and predicting carbohydrate solubility in ionic liquids and validated the approach against a set of experimentally studied systems. We then computationally screened thousands of ILs for cellulose and hemicellulose dissolution^27,32,58^. In addition, we have validated the COSMO-RS model against experimental data for plastic dissolution in ILs and successfully screened 9405 ILs for plastic dissolution^59^.

**Figure 1:**
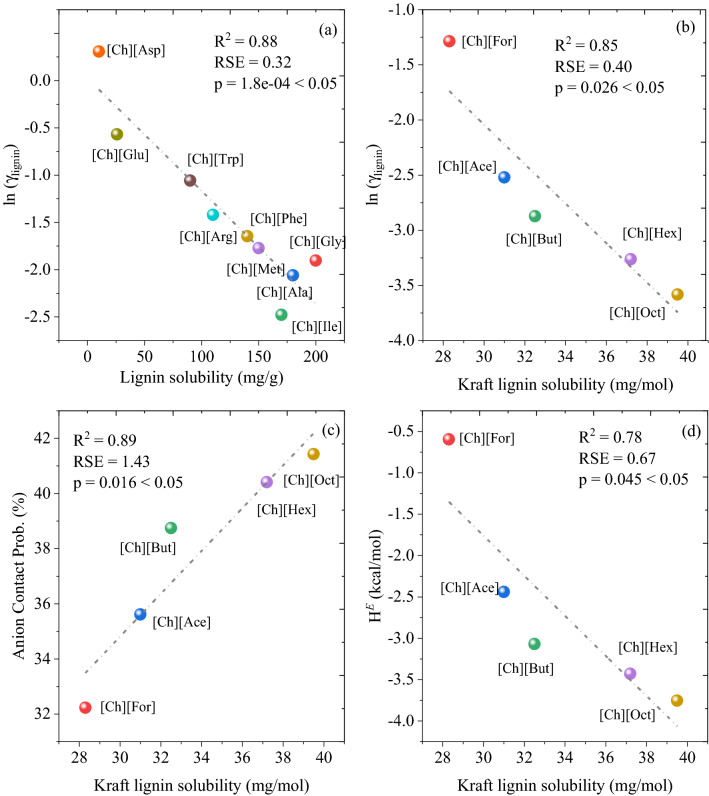
Correlation between the COSMO-RS predicted thermodynamic properties (**a**, **b**) logarithmic activity coefficient (*ln*(*γ*)) versus lignin solubility, (**c**) excess enthalpy, *H*^*E*^, versus lignin solubility, and (**d**) contact probability between lignin-anions versus experimental lignin solubility. COSMOTherm version 19.0.1 was used to calculate the *ln*(*γ*), *H*^*E*^, and contact probabilities (https://www.3ds.com/products-services/biovia/products/molecular-modeling-simulation/solvation-chemistry/biovia-cosmotherm/). (**a**) Data taken from Lie et al.^61^ and (**b**–**d**) data taken from Hou et al.^60^.

In the current work we first performed a series of benchmark studies for dissolution of lignin in a series of cholinium-based ILs by comparing computed thermodynamic properties to experimentally determined lignin solubility (Fig. 1). Figure 1a shows the COSMO-RS predicted ln(γ) against known experimental lignin solubility data taken from the literature^36,60,61^. It was observed that the amino acid based ILs had shown lower *ln*(*γ*) value resulted in higher solubility of lignin. Figure 1b–d shows the correlations between COSMO-RS calculated *H*^*E*^, *ln*(*γ*), and contact probability against experimental lignin dissolution in cholinium-based ILs. Figure 1b–d also elucidating the effect the alkyl chain length of anions on lignin solubility. Similar to *ln*(*γ*), lower values of *H*^*E*^ was associated with greater dissolution capability of lignin in ILs. In contrast, higher values of contact probability were associated with greater dissolution capability of lignin in ILs. Results of the above validation showed good correlation between COSMO-RS calculated thermodynamic properties and experimental lignin dissolution in ILs, indicating calculated *ln*(*γ*), *H*^*E*^ and contact probability should be good predictors of the ability of a specific IL to solubilize lignin. In addition, the solubility of lignin in different classes of ionic liquids was also performed and the results are depicted in Fig. S1. From Fig. S1, the correlation between experimental lignin solubilities and the COSMO-RS predicted activity shows excellent agreement. Therefore, we used the COSMO-RS approach to calculate these thermodynamic properties for an additional 5670 ionic liquids and to predict their propensity toward lignin dissolution. It is important to mention that we have not considered weakly coordinating and halogen-based anions (i.e., [BF4]^−^, [PF6]^−^, Cl^−^, Br^−^, I^−^, F^−^ etc.) for the IL screening study due to their higher melting points and viscosity^62^. Moreover, the current manuscript is a theoretical work and mainly focusses on the development of ILs using multiscale molecular simulation strategies.

In the reported studies^52,53,55,56^, monomeric and dimer structures of lignin were the most popular models, however, these structures do not directly represent the lignin molecule due to the absence of many key linkages present in lignin. The authenticity and objectivity of solute in theoretical models have been largely ignored. In this study, one of our purposes is to establish a good model structure for lignin. Therefore, in order to obtain better theoretical results, a lignin structure was built by joining all the major lignin linkages (β‒O‒4, β‒β, 4‒O‒5, α‒O‒4, and β‒5) present in the native lignin. By performing the quantum chemical calculations, the most stable conformer of lignin structure was obtained and used in the COSMO-RS calculations. The same chemical structure and stable conformer was used in our previous COSMO-RS calculations for lignin in amines, Cyrene, and protic ILs, and the results are in good agreement with experimental lignin solubility^8,63,64^.

The solubility of lignin in different IL species were investigated using the monomeric (coniferyl alcohol and Coumaryl alcohol), dimer (lignin β–O–4 and lignin 4–O–5 linkage), trimer (dibenzodioxocin) and polymeric structure of lignin as model components for the COSMO-RS calculations. In this work, COSMO-RS predicted *ln*(*γ*) and *H*^*E*^ values are correlated with the experimental lignin solubility (Fig. S2)^61^. Linear regression was performed between the COSMO-RS predicted thermodynamics properties (*ln*(*γ*) and *H*^*E*^) and experimental solubilities, and the performance of each lignin model was measured by R-square (R^2^: measures the goodness of data fitting) and the residual sum of squares (RSS), which reflected the prediction accuracy. The R-square of longer chain lignin polymer and trimer (R^2^ = 0.88) were higher among the investigated lignin model structures. However, the RSS values for lignin trimer (i.e., dibenzodioxocin) was smaller than other models. Further, we have performed the linear regression between *H*^*E*^ and lignin solubility (Fig. S2b) and observed that the longer chain lignin polymeric model structure has good R^2^ value (0.78) than other model structures (R^2^ = 0.39–0.41). By considering both regression analysis (*ln*(*γ*) vs. experimental and *H*^*E*^ vs. experimental), the structure which has all the major linkages represents a better model compound for lignin with better predictions.

### COSMO-RS screening of ionic liquids for lignin dissolution

**Figure 2:**
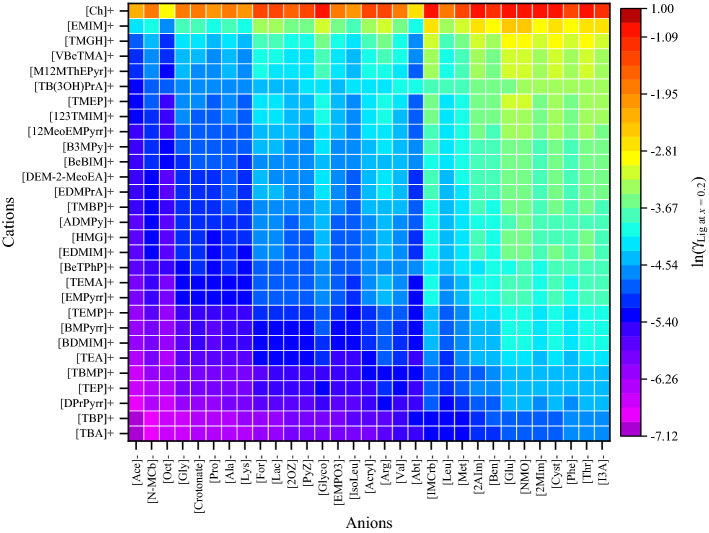
Graphical representation of the logarithmic activity coefficients (ln(*γ*)) of lignin in different ILs at 363.15 K by the COSMO-RS model. Origin2020 SR1 used to draw the heatmaps (https://www.originlab.com/2020).

**Figure 3:**
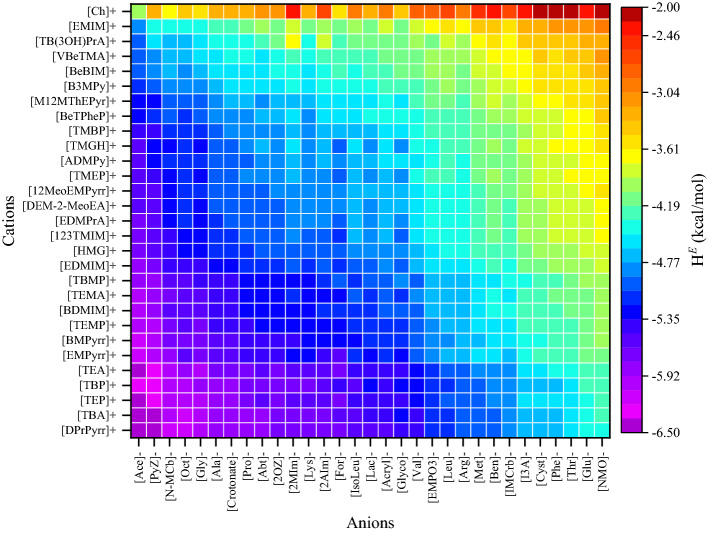
Graphical representation of the excess enthalpy, *H*^*E*^ (kcal/mol) of lignin in different ILs at 363.15 K by COSMO-RS model. Origin2020 SR1 used to draw the heatmaps (https://www.originlab.com/2020).

Ionic liquids can be produced by combining organic cations and organic/inorganic anions, resulting in a wide range of solvents with the potential for polymer (i.e., lignin) dissolution. Experimental screening of the vast number of these combinations for their ability to solubilize lignin is expensive and time-consuming; the ability to accurately predict lignin dissolution in ILs will greatly reduce the cost and time to develop effective solvent systems. In the present study, 63 cations and 90 anions comprising 5670 combinations were screened for lignin dissolution by ranking them based on their ln(γ) and *H*^*E*^ values computed using COSMO-RS model (Figs. 2 and 3). The chemical structures of anions and cations of ionic liquids employed in this work can be seen in Tables S1 and S2, respectively. The COSMO files for all cations and anions were generated based on the procedure outlined in the COSMO-RS calculations section. According to our previous study on plastic-IL screening, the anions and cations were selected with different functional groups^59^. The anions such as amino acid-based anions are considered for the screening study based on the Ohno and Fukumoto^65^ study. Note that some of the investigated cation–anion combinations were not liquids at room temperature, thus the ILs were screened at 363.15 K.

According to the solid–liquid equilibria assumptions^66,67^, the reciprocal of the activity coefficient characterizes lignin solubility in the respective IL. Cations and anions were sorted according to their dissolving capacity and arranged in such a way that ILs with high dissolving power (i.e., *ln*(*γ*) and *H*^*E*^ <  < 0) of lignin are situated in the left and bottom portion of Figs. 2 and 3, and the weaker ones (i.e., *ln*(*γ*) and *H*^*E*^ > 0) are situated at the top portion and right side of Figs. 2 and 3. The anions such as acetate, methyl carbonate, octanoate, glycinate, alaninate, and lysinate are predicted to dissolve lignin more efficiently when in combination with cations like tetraalkylammonium, tetraalkylphosphonium, and pyridinium. This is due to the fact that effective ILs form strong H-bonds, C‒H…π, and cation‒π interactions with lignin. However, the anions such as triflate, gentisate, histidinate, and bis(trifluoromethylsulfonyl)imide have high positive values of *ln*(*γ*). The *ln*(*γ*) and *H*^*E*^ values of lignin in 5670 ILs are provided in Tables S3 and S4. As the alkyl chain length of anion or cation increases, the *ln*(*γ*) and *H*^*E*^ of lignin were seen to be decreasing. For example, in a comparison between the imidazolium-based cations ([12Dmim]^+^, [Amim]^+^, [Emim]^+^, [Bmim]^+^, [Hmim]^+^, and [Omim]^+^) with all 90 investigated anions, the *ln*(*γ*) of lignin decreased (i.e., more negative) as alkyl chain length of the cation increased from [Emim]^+^ to [Omim]^+^ (Fig. S3a). A similar observation was made in the case of anions (Fig. S3b). A contrary observation was reported in the literature, where Wang et al.^38^ reported that the solubility of lignin decreases with increase in the alkyl chain length of cations. This discrepancy may be due to the viscosity of ionic liquids. As the alkyl chain length of cation increases, the viscosity of IL also increased^68^. This higher viscosity of IL restricts the mass transfer rate of liquids which results in the lowering of lignin solubility. A similar observation was also noticed in our previous studies^63^, where spermidine and spermine showed stronger interaction with lignin, but results in lower biomass delignification due to their higher viscosity. Therefore, the combination of longer alkyl chain length of both the cation and anion resulted in weaker solvent for lignin solvation as compared to that of the combination of a highly polar and less polar ions. Based on these observations, the ions of IL should obey the following successive criteria: (1) either of the ions should be a good hydrogen bond acceptor or donor, and (b) another ion to be slightly polar (to weakly coordinate with counter ion thereby reduces the cross interactions between anion and cation). According to this thumb rule, the cations such as tetraalkylammonium, tetraalkylphosphonium, and alkylpyridinium are less polar, and the anions such as acetate, methyl carbonate, glycinate, alaninate, and lysinate are highly polar in nature. The interaction between the polar anion and the lignin is energetically much stronger than the interaction between anion and cation, resulting in a high lignin solvation capability.

Casas et al.^53,55^ performed comprehensive COSMO-RS calculations to screen all combinations of 20 cations and 16 anions of ionic liquids for lignin dissolution. Further, Balaji et al. (2012) screened 1156 ILs for extraction of lignin from biomass based on COSMO-RS calculated solubility parameters. Casas et al.^53^ and Balaji et al.^52^ used lignin monomers as a lignin model compound for the COSMO-RS calculations and reported that 1-ethyl-3-methylimidazolium acetate, tetrabutylammonium fluoride, tetrabutylammonium acetate, and tetrabutylammonium chloride are potential solvents for lignin solvation. However, in our case tetrabutylammonium glycinate, tetrabutylammonium acetate, tetrabutylphosphonium glycinate, and 1,1-dipropylpyrrolidinium acetate are the better ILs for lignin dissolution. The discrepancy between the literature (Casas et al. and Balaji et al.) and results from our study are due to the chemical structure of lignin that is used in the COSMO-RS calculations. Lignin monomers and dimers are rich in hydrogen bond donors (see Fig. S4); thus, they interact with anions (rich in hydrogen bond acceptors). While the polymeric structure of lignin is less polar in nature and exhibits significant hydrophobic sites. Recently, Yu et al.^56^ screened 3886 ILs for the lignin using the COSMO-RS model and reported that 1-propyl-1-methylpyrrolidinium chloride, 1-propyl-1-methylpyrrolidinium acetate, 1-methyl-1,8-diazabicyclo(5.4.0)undec-7-ene chloride, 1-methyl-1,8-diazabicyclo(5.4.0)undec-7-ene acetate, and 1-butyl-1,8-diazabicyclo(5.4.0)undec-7-ene chloride are better ILs for lignin dissolution. In the same study, it was also reported that phosphonium cation-based ILs show lower *ln*(*γ*) values of lignin than ammonium-based ILs. However, the present study results demonstrate that ammonium-based ILs are relatively better solvents for lignin than phosphonium cation-based ILs. Moreover, Yu et al.^56^ ranked the solvents only based on the activity coefficient of lignin, however, excess enthalpy of lignin is also a critical parameter in ranking the solvents^53^. Lignin monomeric and dimeric structures shows the poor linear regression fitting (R^2^ = 0.39–0.41) between the *H*^*E*^ and lignin solubility (Fig. S2b). Therefore, the monomeric and dimer structures of lignin do not directly represent the lignin molecule due to the absence of many key linkages present in lignin. It is important to mention that the ILs based on tetraalkylammonium, tetraalkylphosphonium, and pyrrolidinium had a greater capacity for dissolving lignin. This was because these ILs had weaker interactions between their cations and anions, as a result, the ions of ILs had more opportunities to interact with lignin, thereby enhancing the lignin dissolution capability.

### Sigma potential of anions and cations

**Figure 4:**
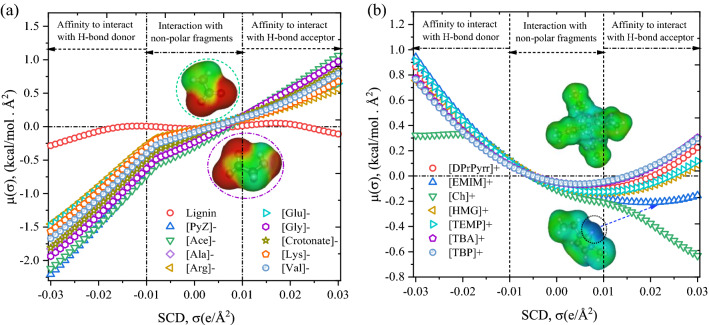
COSMO-RS predicted sigma potential of (**a**) lignin and anions, and (**b**) cations of ILs at 363.15 K. COSMOTherm *version* 19.0.1 was used to calculate the sigma potentials (https://www.3ds.com/products-services/biovia/products/molecular-modeling-simulation/solvation-chemistry/biovia-cosmotherm/).

The sigma potentials (*μ*(*σ*)) of lignin, anions, and cations were calculated to understand the affinity of solvents for the surface polarity of lignin. The *σ*-potentials are divided into three main types: non-polar (green/yellow: − 0.01 e/Å^2^ < *σ* > + 0.01 e/Å^2^), H-bond acceptor (red: *σ* < − 0.01 e/Å^2^), and H-bond donor (blue: *σ* > + 0.01 e/Å^2^) regions (see Fig. 4). The sigma potential, *μ*(*σ*), of lignin is negative in both the negative and positive charge density regions (*σ* < − 0.01 e/Å^2^ and *σ* > + 0.01 e/Å^2^), indicating that lignin has a tendency to interact with both negative and positive polar surfaces of molecule (i.e., H-bond donors and acceptors in the solvent) (Fig. 4a). On the negative screening charge densities side (*σ* > − 0.01 e/Å^2^), the σ-potential value of anions is negative (− 1.5 to − 2.4 kcal/mol Å^2^), implying that anions have more affinity to interact with the positive surface charge density of lignin or cation (i.e., H-bond donors) (Fig. 4a). In contrast, the σ-potential value of anions is positive (0.5–1.0 kcal/mol Å^2^) in the region of positive screening charge densities (*σ* > + 0.01 e/Å^2^), which reflects that the anions lack electron donor surfaces. For cations such as [Ch]^+^ and [Emim]^+^, the *μ*(*σ*) value is negative in the positive screening charge density side (*σ* > + 0.01 e/Å^2^), indicating that [Ch]^+^ and [Emim]^+^ cations have higher affinity toward negatively charged surfaces of lignin and anions (Fig. 4). For system composed of lignin and [Ch]^+^ or [Emim]^+^-based ILs, the anions are more likely to interact with the cations than lignin since [Ch]^+^ and [Emim]^+^ are polar cations with higher tendency to interact with negatively charged surfaces thereby forming stronger electrostatic and hydrogen bondings. Therefore, the logarithmic activity coefficient and excess enthalpy of lignin in such ILs are weaker. However, in the case of [TBA]^+^, [TBP]^+^, and [DPrPyrr]^+^, the cations lack both positive and negative surfaces (positive *μ*(*σ*) value), implying these cations have less tendency to interact with anions i.e., the interaction between anion and cation is weaker. Thus, both the anions and cations have higher odds of interacting with lignin, thereby having a stronger interaction with lignin. Anions interacting through electrostatic and H-bonding interactions with lignin and the [TBA]^+^, [TBP]^+^, and [DPrPyrr]^+^ cations interact with lignin through vdW and cation-*π* interactions.

## Parameters that influence lignin dissolution

### Viscosity and interaction energy between anion and cation of ILs

The viscosity of solvent also plays an important role in the dissolution of a polymer and higher viscosity can restrict the effective mass transfer rate, which is known to have a negative effect on polymer dissolution^27,33,69^. Ionic liquids present a higher viscosity (around 10–726 mPa s) than typical molecular liquids (around 0.2–10 mPa s). The viscosity of an IL depends on the combination of cation–anion and impurities present, and these relationships are well studied in the literature^62^. The ILs with lower excess enthalpy, *H*^*E*^, and lower activity coefficient, *ln*(*γ*), values of lignin should also be cross-checked for viscosity of a solvent. In our previous publication on plastic dissolution in ILs, we predicted the viscosity of ILs using the COSMO-RS model and showed that the predicted values were highly correlated with the experimental viscosities^59^. Therefore, the viscosity of tetrabutylammonium, tetrabutylphosphonium, cholinium, 1-ethyl-3-methylimidazolium, and 1,1-dipropylpyrrolidinium-based cations in combination with amino acid and carboxylate-based anions were predicted using the COSMO-RS model.

**Figure 5:**
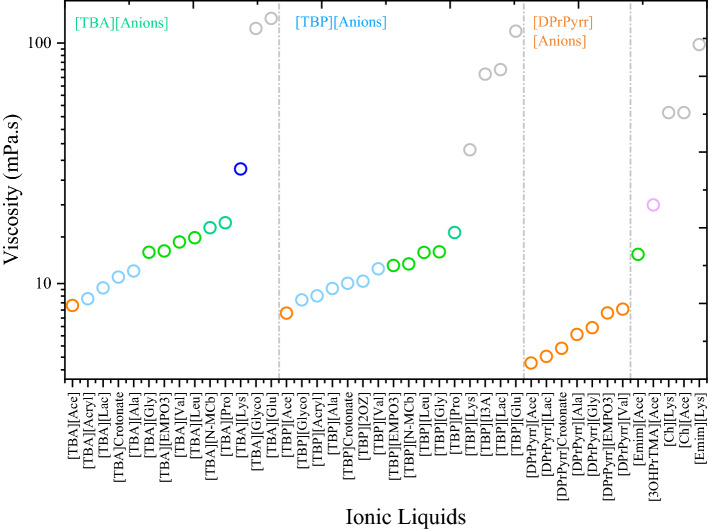
COSMO-RS-based predicted viscosities of pure ionic liquids at 363.15 K. COSMOTherm version 19.0.1 was used to calculate the sigma potentials (https://www.3ds.com/products-services/biovia/products/molecular-modeling-simulation/solvation-chemistry/biovia-cosmotherm/).

For calculating viscosity using the COSMO-RS model, the conformers of each ionic liquid were generated using the Turbomole^70,71^ and BIOVIA COSMOconfX2022 package, and the predicted viscosities are summarized in Fig. 5. The viscosity of liquids are calculated from a quantitative-structure property relationship (QSPR) using molecular descriptors (molecular surface area, *σ*-moments, pure compound entropy, and number of ring atoms in the compound) that are computed by COSMOtherm^72^. From Fig. 5, the viscosity of ammonium, pyridinium, and phosphonium-based ionic liquids had a lower viscosity than cholinium and imidazolium-based ionic liquids. It has been reported that as the alkyl chain lengths of either the cation or anion increased, the viscosity of ionic liquids increased and the H-bond acidity decreased^73^. A similar pattern was also observed in our study. As the alkyl chain length of the anion increased ([Gly]^−^, [Ala]^−^, [Val]^−^, and [Lys]^−^), the viscosity of amino acid-based ILs (e.g.: [TBA][Ala], [TBA][Gly], [TBA][Val], and [TBA][Lys]) increased. The viscosity of ILs with tetraalkylammonium, tetraalkylphosphonium, and pyridinium-based cations was lower than cholinium and imidazolium-based ILs with the same anion (e.g., [Lys]^−^ and [Ace]^−^). On the other hand, the viscosity of ammonium and phosphonium-based ILs were predicted to be lower than the traditional ionic liquids such as [Ch][Lys], [Ch][Ace], and [Emim][Ace]. Similar trends were also observed for interaction energy between anion and cation of ILs (Fig. S5). Tetrabutylammonium, tetrabutylphosphonium, and 1,1-dipropylpyrrolidinium-based ILs have shown lower interaction energies between cation and anion, which is in line with sigma potential observations (Fig. 4). The ILs [Ch][Lys], [Ch][Ace], [Emim][Lys], and [Emim][Ace] showed stronger and higher interaction energies between anion and cations (Fig. S5), which helps explain why the viscosity of cholinium and imidazolium-based ILs are higher than tetraalkylammonium, tetraalkylphosphonium, and pyridinium-based ILs.

### Solubility parameters

**Figure 6:**
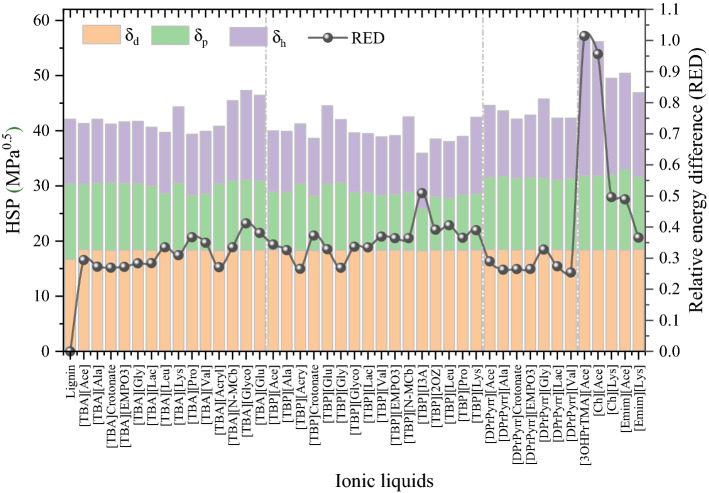
The COSMO-RS-based predicted Hansen Solubility Parameters of ionic liquids. COSMOquick tool version 1.7 is used to predict the HSP of ILs (https://www.3ds.com/products-services/biovia/products/molecular-modeling-simulation/solvation-chemistry/biovia-cosmoquick/).

The solubility parameter (SP) of a molecular species is a vital property that characterizes polarity and quantifies the ‘like-seeks-like' principle, which is used in chemistry to screen solvents for dissolution of solutes. There are several literature reports on the delignification of biomass using ionic liquids^13,38,74–76^. Therefore, we explored Hansen solubility parameters as a measure of the capability of ionic liquids to delignify biomass. Hansen solubility parameters, δ, characterize the affinity of solvents towards polymers dissolution using three parameters δ_*d*_ for dispersion or van der Waals, δ_*p*_ for polarity, which is related to dipole moments, and δ_*h*_ for hydrogen bonding. The solubility parameters of lignin are 13.5 MPa^1/2^ polar (δ_*p*_), 11.3 MPa^1/2^ hydrogen-bonded (*δ*_*h*_), 16.7 MPa^1/2^ dispersion (*δ*_*d*_), and 24.3 MPa^1/2^ total (*δ*_*t*_)^63,77^. The ‘like-seeks-like’ principle suggests that for cases in which solubility parameters of lignin and IL are closer, the affinity between the lignin polymer and the IL will be higher (i.e., ILs with similar solubility parameters as lignin will have greater dissolution capability). The solubility parameters of tetrabutylammonium, tetrabutylphosphonium, cholinium, 1-ethyl-3-methylimidazolium, and 1,1-dipropylpyrrolidinium-based ionic liquids were computed using the COSMO-RS model, and the results are depicted in Fig. 6. The solubility parameters of ILs were calculated using the COSMOquick^78^. The detailed calculation procedure of HSP using COSMOquick is reported elsewhere^79,80^. In our earlier publication on the development of solubility parameters for lignin and ILs, multiscale molecular simulation approaches were used to successfully predict the HSPs of lignin and ILs. It has also been demonstrated that the COSMO-RS model accurately predicts the HSPs of IL without any experimental data, hence here we used the COSMO-RS model to predict the solubility parameters of ILs^80^.

Figure 6 reports the HSPs of lignin and ionic liquids along with their RED (relative energy difference) values of lignin in ILs. The RED values are calculated as the ratio of radius of interaction (R_a_) to the 3D sphere radius of the solute (R_0_)^80^. If RED < 1, then the affinity of the IL towards lignin is said to be high. While If RED > 1, the affinity between the IL and lignin is weaker and leads to lower biomass delignification. From the RED point of view, [TBA]^+^, [TBP]^+^, and [DprPyrr]^+^-based ionic liquids had lower RED values of lignin than [Ch]^+^ and [Emim]^+^-based ILs. The ILs based on [Ch]^+^ and [Emim]^+^ had higher polar and hydrogen-bonded contributions, which is due to the stronger polarity and hydrogen-bonding capability of [Ch]^+^ and [Emim]^+^-based ionic liquids. The larger RED values of [Ch]^+^ and [Emim]^+^-based ILs are due to the higher polarity and hydrogen bonding contributions, which further leads to stronger interactions between anion and cation of the IL. The stronger anion and cation interactions (Fig. S5) result in higher viscosity (Fig. 5) and weaker solvents for lignin dissolution. The RED ranking of anions with [TBA]^+^, [TBP]^+^, and [DprPyrr]^+^-based cations for lignin dissolution was as follows: [Ala]^−^ > [Ace]^−^ > [Gly]^−^ > [Lys]^−^ > [Val]^−^.

### Kamlet–Taft parameters and dissociation constant

**Figure 7:**
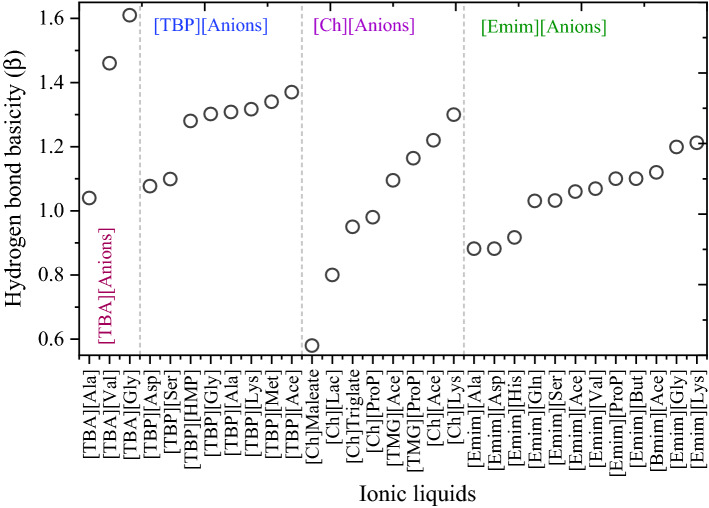
Hydrogen-bond basicity (β) of ionic liquids. The experimental β values are taken from literature.

Kamlet–Taft parameters, first introduced in 1976, are also commonly used solvent scales for predicting the behavior of solvents. The Kamlet–Taft solvent parameter π^*^ defines the polarizability of a molecule, which is the ability of a solvent to stabilize a charge or a dipole by virtue of its dielectric effect. The *α* parameter describes the strength of a hydrogen bond donor (i.e., hydrogen bond acidity), while the *β* parameter is used for describing the strength of a hydrogen bond acceptor (i.e., hydrogen bond basicity). Generally, ILs have high π^*^ and *β* values and moderate *α* values, while *β* primarily depends on the choice of anion and *α* depends on the cation. It was reported that hydrogen bond basicity *β* of IL was the most important parameter for the dissolution of cellulose and lignin, as well as biomass fractionation^56,81^. Dissolution of biopolymers occurs in ILs when the value of *β* is greater than approximately 0.8. The Kamlet–Taft ‘*β’* parameter of tetrabutylammonium, tetrabutylphosphonium, cholinium, and [Emim]-based ILs were taken from the literature and depicted in Fig. 7^82–84^. From Fig. 7, amino acid-based anions had higher *β* values than carboxylate-based anions, indicating that amino acid-based anions in combination with tetrabutylammonium and tetrabutylphosphonium cations could effectively dissolve the lignin and deconstruct biomass. The higher HB basicity of [TBA]^+^ and [TBP]^+^-based ILs were due to the weaker interaction energy between anion and cation of ILs (Fig. S5). The weaker interaction energy between anion and cation of ILs leads to the presence of more free anions, thus the glycinate, valinate, and lysinate-based anions exhibit higher HB basicity values. The ranking of ILs with high *β* values was: [TBA][Gly] > [TBA][Val] > [TBP][Ace] > [TBP][Lys] > [Ch][Lys].

In addition to Kamlet–Taft parameters, the dissociation constant (pK_a_) of ILs plays a critical role in the deprotonation of lignin. Recently, Mohan et al.^7^ reported the efficacy of [Ch][Lys] over [Ch][Oct] for lignin dissolution. The pK_a_ values of lignin protons lie in the range of 1–5 (carboxylic protons) and 6–11 (phenolic protons)^85^. This implies that all lignin protons would be deprotonated (hence enhancing the solubility of lignin) in the presence of a chemical with a similar pK_a_ value or greater than 11. The pK_a_ of carboxylate anions are in the range of 4.27–5.5 (acetate to decanoate), while the pK_a_’s of amino acid-based anions are 2.74 (COOH)–12.41 (NH_2_) (Fig. S6). Amino acid based ILs are therefore able to deprotonate the protons of lignin with a pK_a_ value up to10.3, which implies the deprotonation of almost all the available carboxylic and phenolic protons of lignin, leading to a maximum charge density and higher delignification.

### Molecular dynamics simulations of lignin-ionic liquids

Molecular dynamics simulations are a prevailing computational technique for investigating the interactions among molecules in binary mixtures and are a useful tool for understanding the delignification of lignocellulosic biomass in ionic liquids^7,77^. From the COSMO-RS calculations, [TBA]-based ILs stood out as potential solvents for lignin dissolution, and we investigated biomass delignification with [TBA]-based ILs and compared the results to cholinium lysinate. For MD simulations, lignin model polymer structure is composed of 26 units of G-, S-, and H-units with a molecular weight of ~ 5 kDa (Fig. S7).

### Radius of gyration and solvent-accessible surface area analysis

**Figure 8:**
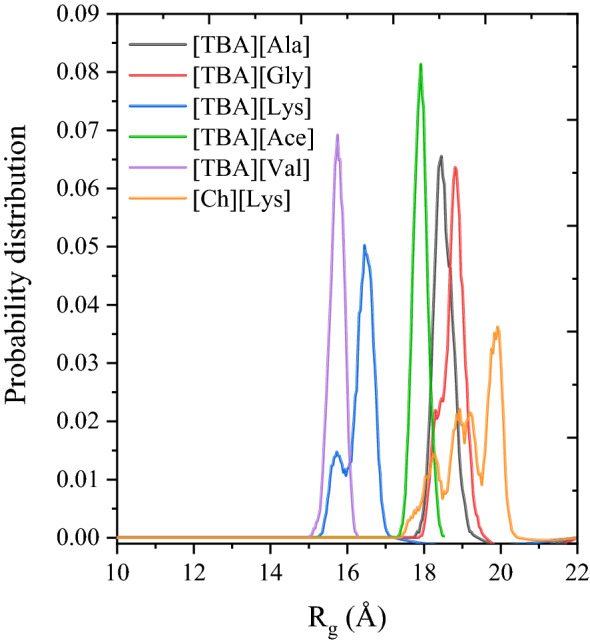
The probability distribution of R_g_ for the lignin polymer in ionic liquids.

The radius of gyration (*R*_*g*_) of lignin (DP-26) in different IL environments were measured to study the compactness of the lignin polymer structure. For polymers, a lower *R*_*g*_ corresponds to the collapsed-like structure, whereas a larger value of *R*_*g*_ corresponds to the coil or extended polymer-like structure. Figure 8 depicts the probability distribution of the radius of gyration of lignin in different IL systems. The average *R*_*g*_ values were 15.75 Å, 17.92 Å, 18.45 Å, 18.85 Å, and 19.96 Å for [TBA][Val], [TBA][Ace], [TBA][Ala], [TBA][Gly], and [Ch][Lys], respectively. According to this *R*_*g*_ values (> 14 Å), lignin adopted an extended polymer-like structure, while if the *R*_*g*_ value of lignin is lower than 13 Å, lignin forms collapsed compact conformation which was generally obtained in the water environment^86^. Representative snapshots of lignin (i.e., coil-like structure) from the simulations in ILs were shown in Fig. S8.

**Figure 9:**
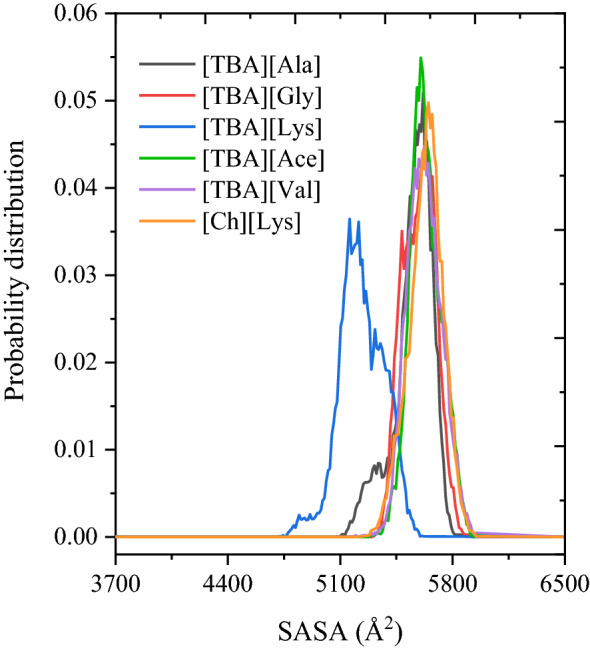
The probability distribution of SASA for the lignin polymer in ionic liquids.

The solvent-accessible surface area (SASA) of a polymer is a useful metric for measuring the interface between a solute and its solvent i.e., the surface area of solute that is in contact with the solvent in which it resides. For solutes, hydrophobicity increases with a decreasing SASA. The probability distribution of lignin SASA in the investigated IL systems is illustrated in Fig. 9. From Fig. 9, the SASA of lignin in all investigated ILs was similar and higher than 3200 Å^2^, indicating lignin makes more energetically favorable interactions with the solvent than with itself, resulting in solubilization of lignin. The conclusions drawn from the SASA’s probability distribution analysis support those observed from the analysis of *R*_*g*_’s distribution.

### Radial distribution functions and intra and intermolecular hydrogen bonds of lignin

**Figure 10:**
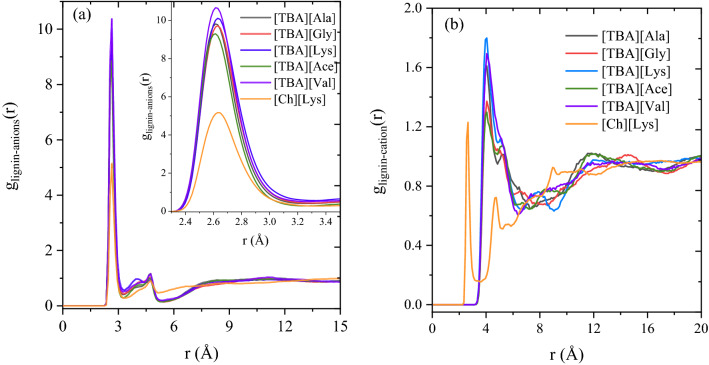
RDF plot between the O atom of lignin with (**a**) O1 atom of anion, and (**b**) N1 atom of cation for different lignin-IL systems.

To obtain the structural arrangements and microscopic interactions, radial distribution functions (*g*(*r*) or RDF) were computed between lignin and the IL solvent. Radial distribution functions are the probability of finding a molecule at a distance of ‘*r*’ from the reference molecule^7^. The RDF plots are a useful tool for understanding the structural and explicit interactions of lignin and solvents. In our case, the RDFs are plotted between the oxygen (O) atom of lignin molecule with O1 (acceptor) atom of anion and N atom of cations, and the results are depicted in Fig. 10a,b.

**Figure 11:**
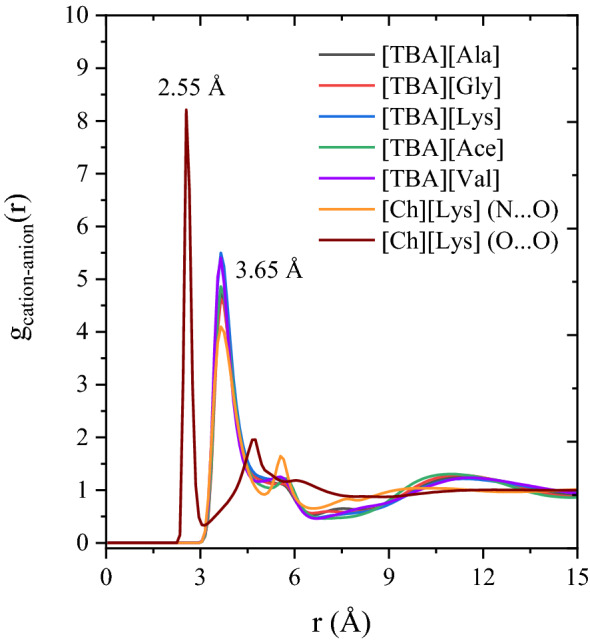
RDF plot between the O atom of anion with N1 atom of cation in different lignin-IL systems.

Figure 10a shows the RDF plot between the lignin and anions of ILs. The first and largest solvation shell of RDF peak between lignin and anion was attained at 2.65 Å with a *g*(*r*) peak height of 5–10, indicates that anion forms regular and definite coordination spheres around lignin moiety at a distance of 2.65 Å and the RDF plot is primarily dominated by the first coordination shell. The secondary solvation shells are less ordered when compared to the first solvation shell (Fig. 10a). It is interesting to note that the height of RDF peak *g*(*r*) for lignin-anion from [TBA]-based ILs are ~ 10, implying that the contact probability between lignin and anion of [TBA][-ILs] are almost 10 times in their first solvation shell (3.45 Å), which is higher than [Ch][Lys]’s RDF peak. In the case of lignin-cation RDFs, cations also form a regular and well-ordered shell around the lignin molecule, where the [TBA]^+^ cation approaches the lignin moiety at a distance of 4.0 Å, while the [Ch]^+^ approaches lignin moiety at a distance of 2.65 Å. For the [TBA]-based systems, the cation [TBA]^+^ had higher *g*(*r*) value of lignin molecule than [Ch]^+^ cation, indicating that the contact probability between [TBA]^+^ cation and lignin is stronger than [Ch]^+^-lignin. On the other hand, the RDF peak between anion and cation of IL were also measured and depicted in Fig. 11. From Fig. 11, the peak of first solvation shell between lysinate and cholinium was obtained at distance of 2.65 Å with a peak height of ~ 8, indicating that the interactions between anion and cation of [Ch][Lys] were much stronger than the [TBA]^+^-based ILs (*g*(*r*) peak heights are in the range of 4.5–5). The stronger the anion and cation contact probability, the weaker the solvation capability of a solvent because of its higher viscosity. From these findings, it is confirmed that lignin solubility predominantly depends on and can thus be manipulated by the choice of the anion and cation of IL. The less polar cation and high polar anions results in stronger van der Waals interactions and electrostatic interactions with lignin, thereby stabilizing the lignin molecule and enhancing the lignin solubility.

**Figure 12:**
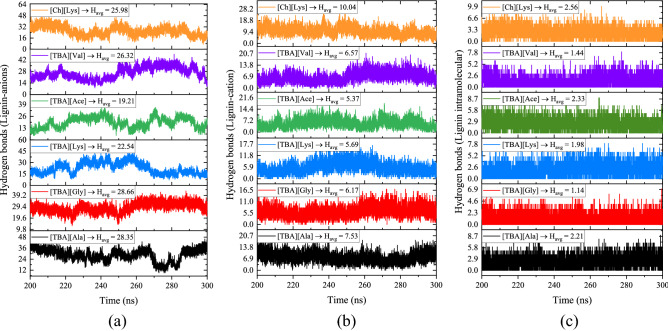
MD calculated average number of hydrogen bonds between the (**a**) lignin-anion, (**b**) lignin-cation, and (**c**) within the lignin molecules in different IL-lignin systems. Origin 2020 SR1 software is used to draw this plot (https://www.originlab.com/2020).

In addition to the R_g_, SASA, and RDF plots, the average number of hydrogen bonds (HBs) per lignin molecule were calculated to further understand the structural rigidity of lignin polymer. The intra and inter-molecular hydrogen-bonding patterns in the lignin polymer and lignin-ILs were monitored as a function of simulation time and the results are shown in Fig. 12. Figure 12a shows the intermolecular hydrogen bonding between lignin and anions of the various ILs. From Fig. 12a, amino acid-based anions ([Gly]^−^, [Ala]^−^, [Val]^−^, and [Lys]^−^) had a higher number of hydrogen bonds with lignin than carboxylate-based anion (e.g. [Ace]^−^), indicating that amino acid anion forms multiple hydrogen bonds with lignin than acetate. However, [TBA][Ala], [TBA][Gly], and [TBA][Val] formed a marginally higher number of hydrogen bonds with lignin than [Ch][Lys]. This may be due to the formation of additional hydrogen bonds between cholinium and lysinate ions. On the other hand, HBs between lignin and cation of ILs were also characterized and are shown in Fig. 12b. [TBA]^+^ cation had a lower number of H-bonds with lignin than [Ch]^+^ which is due to the higher polarity of the cholinium cation. The additional hydroxyl group of cholinium forms HBs with lignin, resulting in a higher number of lignin-[Ch]^+^ H-bonds. The average number of intramolecular H-bonds within the lignin molecule was also measured and [Ch][Lys] had a higher number of intrachain H-bonds (Fig. 12c). The higher number of intramolecular H-bonds of lignin in [Ch][Lys] is due to the weaker hydrogen bonding dynamics of lignin-anion and lignin-cation, which is further evidenced by their RDF plots (lower contact probability). The significant loss of intrachain H-bonds in [TBA][Gly] and [TBA][Val] ILs revealed effective lignin dissolution in these solvent environments. The breakdown of intramolecular hydrogen bonds of lignin could be due to the favorable interactions between the lignin polymer and [TBA]-based ILs. On the other hand, the interaction within the lignin molecule is less favorable in [TBA]-based ILs, thus could lead to a better solvent for lignin.

### Mass fractal dimension factor

The mass fractal dimension (α) factor, which is related to the solubility of a polymer (as α below 2 indicates a good solvent, while above indicates a poor solvent). The mass fractal dimension factor was obtained by fitting the relationships between lignin size and structural observables such as the radius of gyration (*R*_*g*_) and solvent accessible surface area (SASA). The summary of molecular dynamics simulations for different lignin polymers in [TBA][Gly] was presented in Table S5 and the chemical structures and linkages of lignin are reported in Tables S6–S16. Within the (bio)polymer physics, these observables are typically related through a power law and the governing equation of scaling properties are as follows:^87,88^

3 $$O = AM^{\nu }$$

4 $$\alpha = \frac{2}{5\nu - 1}$$

The power law described in Eq. 3 is used to fit the relationships between structural observables, *O*, such as *R*_*g*_ and SASA, and the molecular weight, *M*, of lignin polymers. The fitting parameters *A* and particularly ‘*ν*’ have meaning when interfaced with polymer theory and used to compute the mass fractal dimension (*α*) factor (Eq. 4).

**Figure 13:**
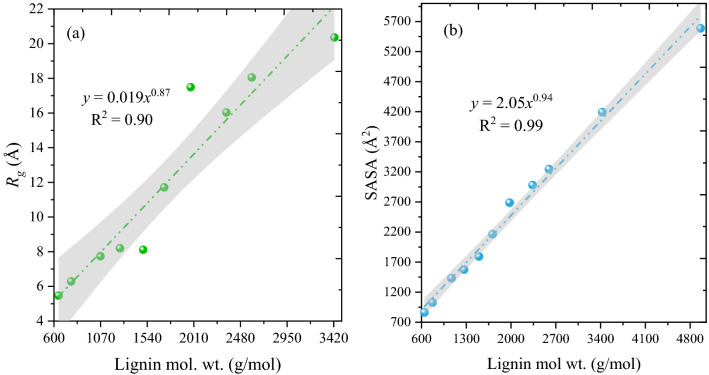
Relationship between (**a**) *R*_*g*_, and (**b**) SASA of lignin and the molecular weight of the lignin polymer.

Figure 13 shows the relationship between the structural observables, *R*_*g*_/SASA and molecular weights of lignin polymers. From 13a, the *R*_*g*_ is sensitive to lignin polymer molecular weight and the scaling parameter ‘*ν*’ is > 0.334, reflective a good solvent in polymer theory^88^. On the other hand, SASA for a given lignin polymer is largely determined by its molecular weight and variation within SASA values within a trajectory is comparatively smaller than it was for *R*_*g*_. For a sphere of uniform density, the SASA should increase with the M^2/3^_sphere_. The scaling parameter *ν* > 0.67 (i.e., 0.94), indicating that there is an excess surface area for real lignin polymers relative to an ideal spherical globule^87,88^. Mass-fractal dimensions *α*, closer to 1 (i.e., below 2) are “good” solvents while fractal dimensions near 3 or above are “bad/poor” solvents. From the literature, in water, the *α* value of lignin is greater than 3 and thus water is a poor solvent for lignin dissolution^87,89^.

## Conclusions

The present study reports the screening of large number of ionic liquids for the dissolution of lignin using the COSMO-RS model. A total of 5670 ILs (combination of 63 cations and 90 anions) were screened for lignin by predicting the logarithmic activity coefficient (*ln*(*ϒ*)) and excess enthalpies (*H*^*E*^) of lignin in ILs. Based on the COSMO-RS predictions, it can be concluded that anions such as acetate, methyl carbonate, glycinate, alaninate, and lysinate are predicted to dissolve the lignin more efficiently in combination with the cations like tetraalkylammonium, tetraalkylphosphonium, and pyridinium. After screening 5670 ILs, the dissolution properties such as interaction energy between anion and cation, viscosity, Hansen solubility parameters, dissociation constants, and Kamlet-Taft parameters have been evaluated to assess the lignin dissolution. It has been noticed that [TBA]^+^ and [TBP]^+^-based ILs were seen to be potential solvents for lignin dissolution as they show weaker interactions with their counter ion, possess lower viscosity, closer HSP values, and higher hydrogen bonding basicity. Furthermore, MD simulations were performed for lignin and [TBA]-based ILs and the results were compared with [Ch][Lys]. MD simulation results show that the lignin adopts an extended polymer-like structure (higher *R*_*g*_ and SASA) in both [TBA]^+^ and [Ch]-based ILs, however, the contact probability and the intermolecular hydrogen bonds between lignin and [TBA]-based ILs were stronger than that of lignin and [Ch][Lys], revealing the potential capabilities of [TBA]-based ILs for lignin dissolution. It is worth mentioning that the cation and anion of IL play a crucial role in the dissolution of lignin. In addition, the mass fractal dimensional (*α*) factor was also evaluated for [TBA][Gly] to assess the potential capability of [TBA]-based ILs and observed that the value of ‘*α*’ factor below 1 results in a good solvent for lignin. This work provides an effective strategy for fast screening of appropriate IL for biomass delignification and the analysis of obtained results help to comprehend the dissolution mechanism of lignin in ionic liquids.

## Computational details

### COSMO-RS calculations

The COSMO-RS calculations were carried out to measure the capability of ILs for lignin dissolution based on the activity coefficient and excess enthalpy of lignin in ILs. The geometries of all the investigated lignin polymer and ions (anions and cations) of ionic liquids were generated in the Avogadro software *version* 1.2.0^90^. The geometries of investigated molecules were fully optimized using *Gaussian09* package at B3LYP theory and 6–311 + G(d, p) basis set^91–93^. COSMO files were generated at the BVP86/TZVP/DGA1 level of theory and basis set using the keyword "scrf = COSMORS"^51,58,94^. The generated COSMO files were then used as an input to the COSMOtherm (version 19.0.1, COSMOlogic, Leverkusen, Germany) package with BP_TZVP_19 parametrization, which was used to calculate the sigma potentials, viscosity, solubility parameters of ILs, and the logarithmic activity coefficient (ln(*γ*)) and excess enthalpy (*H*^*E*^) of binary mixtures (lignin and IL)^95,96^.

The logarithmic activity coefficient (ln(*ϒ*)) of component *i* is related to the chemical potential *μ*_i_ is given as following Equations^7,97^:

1 $$\ln \left( {\gamma_{i} } \right) = \left( {\frac{{\mu_{i} - \mu_{i}^{0} }}{RT}} \right)$$

where *μ*_*i*_^*0*^ is the chemical potential of the pure component *i*, *R* and *T* are the real gas contact and absolute temperature. Excess enthalpy of a binary mixture was calculated using Eq. (2)^94^

2 $$H_{M}^{E} = \sum {x_{i} H_{i}^{E} = \sum {x_{i} \left[ {H_{(i,mixture)} - H_{(i,pure)} } \right]} }$$

where *H*_*m*_^*E*^ is the excess enthalpy of each molecule in the mixture, defined as the enthalpy difference between component *i* in the mixture and in the pure state, and the total excess enthalpy of a mixture is the algebraic sum of electrostatic (misfit), hydrogen bonding, and van der Waals interactions. To calculate *H*^*E*^ and ln(*γ*) in the COSMO-RS model, the mole fractions of lignin, cation, and anion was set to 0.2, 0.4, and 0.4, respectively. More details of COSMO-RS calculation in predicting the excess enthalpies and activity coefficients are provided in the COSMOtherm’s user Manual^98^.

In general, ILs might either be modelled by different ‘conformers’ with respect to the relative arrangement of their ions in the COSMO calculation, or simply as two independent, equimolar, ionic species, whose sigma profiles can be combined freely. The second approach is much efficient and was successfully implemented in earlier works^27,54,59,66^. Similar to that, it is carried out in this study. After screening thousands of ILs using the COSMO-RS model, separate quantum chemical (QC) calculations were performed for ILs (combined equimolar ionic species) to predict the structural properties such as viscosity, solubility parameters, and interaction energies between anion and cation of ILs. We performed a search for conformations of ionic liquids using the Turbomole^70,71^ and BIOVIA COSMOconfX2022 program (version 22.0.0, COSMOlogic, Leverkusen, Germany), which automatically identifies conformers relevant for subsequent COSMO-RS calculations. COSMO calculations within the COSMOConf were performed using the BP-TZVP method and basis set.

### Molecular dynamics simulations

Molecular dynamics (MD) simulations of lignin in tetrabutylammonium glycinate [TBA][Gly], tetrabutylammonium alaninate [TBA][Ala], tetrabutylammonium acetate [TBA][Ace], tetrabutylammonium lysinate [TBA][Lys], tetrabutylammonium valinate [TBA][Val], and cholinium lysinate [Ch][Lys] were performed to understand the effect of ILs on the delignification of lignocellulosic biomass. The ILs [TBA][Gly], [TBA][Ala], [TBA][Ace], [TBA][Lys], [TBA][Val], and [Ch][Lys] were chosen based-on the COSMO-RS predictions. The lignin model polymer structure is taken from our previous study^77^ and composed of 26 units of G-, S-, and H-units with a molecular weight of ~ 5 kDa (Fig. S7). To build the lignin structure, all feasible lignin linkages were joined together, and the sequence of the polymer is shown in Table S6. All MD simulations were carried out using the NAMD package^99^ and the CHARMM force field parameters were used for all the investigated molecules. The force field parameters for lignin was taken from Vermaas et al.^100^ and for ILs, the force field parameters were developed by following CHARMM-GUI tool^101,102^.

The initial configuration for all the investigated systems was prepared using PACKMOL^103^. The lignin molecule was solvated in five different [TBA]-based ILs and the results were compared with [Ch][Lys]. The simulation details such as the number of solvent molecules, lignin molecules, and final box size are summarized in Table S17. The potential energy of the system was first minimized for 200,000 steps using a steepest-descent algorithm. After minimization, the system was heated and equilibrated for 15 ns under the NPT ensemble using the Langevin thermostat and Nose–Hoover Langevin barostat^104,105^. The SHAKE algorithm was implemented to constrain all bonds involving hydrogen atoms^106^. The Particle Mesh Ewald (PME) method was implemented to treat long-range electrostatic interactions at a cut-off distance of 12 Å with an accuracy of 10^–6^ (PME tolerance)^107^. Three separate production runs for 300 ns were performed on each simulated system, starting with a different velocity distribution. A 2 femtoseconds (fs) time step was used to integrate the equations of motion and the coordinates of the system were saved every 5 ps for structural and dynamics analysis. All MD simulation trajectories were visualized and analyzed by using TCL scripts, TRAVIS package, and Visual Molecular Dynamics (VMD) tool^108–110^. The non-bonded interaction energies and the number of hydrogen bonds between lignin and ILs were calculated per mole of lignin.

## Supplementary Information

Supplementary Information.

## Data Availability

All data generated or analyzed during this study are included in this published article and its Supplementary Information file.
