# Supplementary material for: Multiscale molecular simulations for the solvation of lignin in ionic liquids
Source: Sci Rep. 2023 Jan 6;13:271. doi: 10.1038/s41598-022-25372-2 (PMC9822913; doi:10.1038/s41598-022-25372-2)
Supplement: Supplementary file 1 — Supplementary Information. [file 41598_2022_25372_MOESM1_ESM.docx]

***Supporting Information***

**Multiscale Molecular Simulations for the Solvation of Lignin in Ionic Liquids**

Mood Mohan^1,2^, Blake A. Simmons^1,3^, Kenneth L. Sale^1,4^, and Seema Singh^*,1,2^

^1^ Deconstruction Division, Joint BioEnergy Institute, 5885 Hollis Street, Emeryville, California 94608, United States

^2^ Bioresource and Environmental Security Department, Sandia National Laboratories, 7011 East Avenue, Livermore, California 94551, United States

^3^ Biological Systems and Engineering Division, Lawrence Berkeley National Laboratory, 1 Cyclotron Road, Berkeley, California 94720, United States

^4^ Department of Computational Biology and Biophysics, Sandia National Laboratories, 7011 East Avenue, Livermore, California 94551, United States

^*^Corresponding Author

E-mail: *ssingh@lbl.gov;* *seesing@sandia.gov* (Seema Singh)

**Table S1.** List of cations used in this work

| Sl. No. | Name of the cation and Acronym | Chem. Formula / mol. wt. | Chemical structure |
| --- | --- | --- | --- |
|  | Acetylcholine  [ACh]^+^ | C_7_H_16_NO_2_^+^  146.21 |  |
|  | Benzyltrimethylammonium  [BeTMA]^+^ | C_10_H_16_N^+^  150.24 |  |
|  | Cholinium  [Ch]^+^ | C_5_H_14_NO^+^  104.17 |  |
|  | (3-hydroxypropyl)trimethylammonium  [(3OH)PrTMA]^+^ | C_6_H_16_NO^+^  118.20 |  |
|  | Diethylmethyl(2-methoxyethyl)ammonium  [DEM-2-MeoEA]^+^ | C_8_H_20_NO^+^  146.25 |  |
|  | Ethyldimethylpropylammonium  [EDMPrA]^+^ | C_7_H_18_N^+^  116.23 |  |
|  | Triethylammonium  [TEM]^+^ | C_6_H_16_N^+^  102.20 |  |
|  | Diethylmethylammonium  [DEMA]^+^ | C_5_H_14_N^+^  88.17 |  |
|  | N,N-dimethyl-N-butyl ammonium  [DMBA]^+^ | C_6_H_16_N^+^  102.20 |  |
|  | Bis(2-hydroxyethyl)dimethylammonium  [D(OH)EDMA]^+^ | C_6_H_16_NO_2_^+^  134.20 |  |
|  | Tris(2-hydroxyethyl)methylammonium  [Tris(2OH)EMA]^+^ | C_7_H_18_NO_3_^+^  164.22 |  |
|  | 1,1-dimethyluronium  [DMUr]^+^ | C_3_H_9_N_2_O^+^  89.12 |  |
|  | N,N-dimethyl-N-octyl ammonium  [DMOA]^+^ | C_10_H_24_N^+^  158.31 |  |
|  | Methyltrioctylammonium  [MTOA]^+^ | C_25_H_54_N^+^  368.71 |  |
|  | Tetrabutylammonium  [TBA]^+^ | C_16_H_36_N^+^  242.47 |  |
|  | Tributyl-(3-hydroxypropyl)ammonium  [TB(3OH)PrA]^+^ | C_15_H_34_NO^+^  244.44 |  |
|  | Tetraethylammonium  [TEA]^+^ | C_8_H_20_N^+^  130.25 |  |
|  | 2-(dimethylamino)-N,N,N-trimethylethan-1-aminium  [DMA-TMA]^+^ | C_7_H_19_N_2_^+^  131.24 |  |
|  | Tetramethylammonium  [TMA]^+^ | C_4_H_12_N^+^  74.15 |  |
|  | Triethylmethylammonium  [TEMA]^+^ | C_7_H_18_N^+^  116.23 |  |
|  | Vinylbenzyltrimethylammonium  [VBeTMA]^+^ | C_12_H_18_N^+^  176.28 |  |
|  | N-ethyl-N-(furan-2-ylmethyl)ethanaminium  [FurEt2NH]^+^ | C_9_H_16_NO^+^  154.23 |  |
|  | N-ethyl-N-(4-hydroxy-3-methoxybenzyl)ethanaminium  [VanEt2NH]^+^ | C_12_H_20_NO_2_^+^  210.30 |  |
|  | N-ethyl-N-(4-methoxybenzyl)ethanaminium  [p-AnisEt2NH]^+^ | C_12_H_20_NO^+^  194.30 |  |
|  | Benzyldimethyl(2-hydroxyethyl)ammonium  [BeDM(2OH)EA]^+^ | C_11_H_18_NO^+^  180.27 |  |
|  | 1-allyl-3-methylimidazolium  [AMIM]^+^ | C_7_H_11_N_2_^+^  123.18 |  |
|  | 1-benzyl-3-butylimidazolium  [BeBIM]^+^ | C_14_H_19_N_2_^+^  215.32 |  |
|  | 1-benzyl-3-ethoxymethylimidazolium  [BeEtoMIM]^+^ | C_13_H_17_N_2_O^+^  217.29 |  |
|  | 1-benzyl-3-methylimidazolium  [BeMIM]^+^ | C_11_H_13_N_2_^+^  173.24 |  |
|  | 1-butyl-2,3-dimethylimidazolium  [BDMIM]^+^ | C_9_H_17_N_2_^+^  153.25 |  |
|  | 1-butyl-3-methylimidazolium  [BMIM]^+^ | C_8_H_15_N_2_^+^  139.22 |  |
|  | 1-ethyl-2,3-dimethylimidazolium  [EDMIM]^+^ | C_7_H_13_N_2_^+^  125.19 |  |
|  | 1,2,3-trimethylimidazolium  [123TMIM]^+^ | C_6_H_11_N_2_^+^  111.17 |  |
|  | 1, 3-dimethylimidazolium  [13DMIM]^+^ | C_5_H_9_N_2_^+^  97.14 |  |
|  | 1-ethyl-3-methylimidazolium  [EMIM]^+^ | C_6_H_11_N_2_^+^  111.17 |  |
|  | 1-hexyl-3-methylimidazolium  [HMIM]^+^ | C_10_H_19_N_2_^+^  167.28 |  |
|  | 1-octyl-3-methylimidazolium  [OMIM]^+^ | C_12_H_23_N_2_^+^  195.33 |  |
|  | 1-(2-hydroxyethyl)-3-methylimidazolium  [12HyE3MIM]^+^ | C_6_H_11_N_2_O^+^  127.17 |  |
|  | 1-(3-((3-carboxypropanoyl)oxy)propyl)-3-methylimidazolium  [CPOPMIM]^+^ | C_11_H_17_N_2_O_4_^+^  241.27 |  |
|  | Benzyltriphenylphosphonium  [BeTPhP]^+^ | C_25_H_22_P^+^  353.42 |  |
|  | Tetrabutylphosphonium  [TBP]^+^ | C_16_H_36_P^+^  259.44 |  |
|  | Tetraethylphosphonium  [TEP]^+^ | C_8_H_20_P^+^  147.22 |  |
|  | (2-hydroxyethyl)trimethyl phosphonium  [TMEOHP]^+^ | C_5_H_14_OP^+^  121.14 |  |
|  | Triethylmethylphosphonium  [TEMP]^+^ | C_7_H_18_P^+^  133.19 |  |
|  | Tributylmethylphosphonium  [TBMP]^+^ | C_13_H_30_P^+^  217.36 |  |
|  | Trimethylbutylphosphonium  [TMBP]^+^ | C_7_H_18_P^+^  133.19 |  |
|  | Trimethylethylphosphonium  [TMEP]^+^ | C_5_H_14_P^+^  105.14 |  |
|  | Trihexyltetradecylphosphonium  [THTeDP]^+^ | C_32_H_68_P^+^  483.87 |  |
|  | 1-ethylurea-3-methylimidazolium  [EUrMIM]^+^ | C_6_H_11_N_4_O^+^  155.18 |  |
|  | 2-ethyl-1,1,3,3-tetramethylguanidinium  [2ETeG]^+^ | C_7_H_18_N_3_^+^  144.24 |  |
|  | 1,1,3,3-tetramethylguanidine  [TMGH]^+^ | C_5_H_14_N_3_^+^  116.19 |  |
|  | Guanidinium  [Gun]^+^ | CH_6_N_3_^+^  60.08 |  |
|  | Hexamethylguanidinium  [HMG]^+^ | C_7_H_18_N_3_^+^  144.24 |  |
|  | 1-allyl-3,4-dimethylpyridinium  [ADMPy]^+^ | C_10_H_14_N^+^  148.23 |  |
|  | 1-butyl-3-methylpyridinium  [B3MPy]^+^ | C_10_H_16_N^+^  150.24 |  |
|  | 1-butylpyridinium  [BPy]^+^ | C_9_H_14_N^+^  136.22 |  |
|  | 1-ethylpyridinium  [EPy]^+^ | C_7_H_10_N^+^  108.16 |  |
|  | 1,1-dipropylpyrrolidinium  [DPrPyrr]^+^ | C_10_H_22_N^+^  156.29 |  |
|  | 1-methylpyrrolidinium  [MPyrr]^+^ | C_5_H_12_N^+^  86.16 |  |
|  | 1-(2-methoxyethyl)-1-methylpyrrolidinium  [12MeoEMPyrr]^+^ | C_8_H_18_NO^+^  144.24 |  |
|  | 1-methyl-1-(2-(methylthio)ethyl)pyrrolidinium  [M12MThEPyr]^+^ | C_8_H_18_NS^+^  160.30 |  |
|  | 1-butyl-1-methylpyrrolidinium  [BMPyrr]^+^ | C_9_H_20_N^+^  142.27 |  |
|  | 1-ethyl-1-methylpyrrolidinium  [EMPyrr]^+^ | C_7_H_16_N^+^  114.21 |  |

**Table S2.** List of anions used in this work

| Sl. No. | Name of the anion and Acronym | Chem. formula & mol. wt. | Chemical structure |
| --- | --- | --- | --- |
|  | Alaninate  [Ala]^-^ | C_3_H_6_NO_2_^-^  88.09 |  |
|  | Argininate  [Arg]^-^ | C_6_H_13_N_4_O_2_^-^  173.20 |  |
|  | Asparaginate  [Aspg]^-^ | C_4_H_7_N_2_O_3_^-^  131.11 |  |
|  | Cysteinate  [Cyst]^-^ | C_3_H_6_NO_2_S^-^  120.15 |  |
|  | Glutaminate  [Glu]^-^ | C_5_H_9_N_2_O_3_^-^  145.14 |  |
|  | Glycinate  [Gly]^-^ | C_2_H_4_NO_2_^-^  74.06 |  |
|  | Histidinate  [His]^-^ | C_6_H_8_N_3_O_2_^-^  154.15 |  |
|  | Isoleucinate  [Isole]^-^ | C_6_H_12_NO_2_^-^  130.17 |  |
|  | Leucinate  [Leu]^-^ | C_6_H_12_NO_2_^-^  130.17 |  |
|  | Lysinate  [Lys]^-^ | C_6_H_13_N_2_O_2_^-^  145.18 |  |
|  | Methioninate  [Met]^-^ | C_5_H_10_NO_2_S^-^  148.20 |  |
|  | Phenylalaninate  [Phe]^-^ | C_9_H_10_NO_2_^-^  164.18 |  |
|  | Prolinate  [Pro]^-^ | C_5_H_8_NO_2_^-^  114.12 |  |
|  | Asparate monoanion  [AspMA]^-^ | C_4_H_6_NO_4_^-^  132.10 |  |
|  | Glutamate monoanion  [GluMA]^-^ | C_5_H_8_NO_4_^-^  146.12 |  |
|  | Serinate  [Ser]^-^ | C_3_H_6_NO_3_^-^  104.09 |  |
|  | Threoninate  [Thr]^-^ | C_4_H_8_NO_3_^-^  118.11 |  |
|  | Tryptophanate  [Try]^-^ | C_11_H_11_N_2_O_2_^-^  203.22 |  |
|  | Tyrosinate  [Tyro]^-^ | C_9_H_10_NO_3_^-^  180.18 |  |
|  | Valinate  [Val]^-^ | C_5_H_10_NO_2_^-^  116.14 |  |
|  | 2-Aminoimidazolate  [2AIm]^-^ | C_4_H_4_N_3_O_2_^-^  126.10 |  |
|  | 2-methylimidazolate  [2MIm]^-^ | C_5_H_5_N_2_O_2_^-^  125.11 |  |
|  | 5,6-dimethylbenzimidazolate  [56DMBeIm]^-^ | C_10_H_9_N_2_O_2_^-^  189.19 |  |
|  | Purin-7-ide  [Pur]^-^ | C5H3N4-  119.11 |  |
|  | 2-oxooxazolidin-3-ide  [2OZ]^-^ | C_3_H_4_NO_2_^-^  86.07 |  |
|  | Pyrazol-1-ide  [PyZ]^-^ | C_3_H_3_N_2_^-^  67.07 |  |
|  | 2-nitroimidazol-1-ide  [2NIm]- | C_3_H_2_N_3_O_2_^-^  112.07 |  |
|  | Benzimidazolate  [BeIm]^-^ | C_8_H_5_N_2_O_2_^-^  161.14 |  |
|  | 2-(chloromethyl)acrylate  [2ClMAcr]- | C_4_H_4_ClO_2_^-^  119.52 |  |
|  | 2-(Bromomethyl)acrylate  [2-BrMAcr]^-^ | C_4_H_4_BrO_2_^-^  163.98 |  |
|  | 2-(hydroxymethyl)acrylate  [2OHMAcr]- | C_4_H_5_O_3_^-^  101.08 |  |
|  | Chloroacetate  [2-ClAce]^-^ | C_2_H_2_ClO_2_^-^  93.49 |  |
|  | 3-hydroxy-2-methylenebutyrate  [3-(OH)2-MB]^-^ | C_5_H_7_O_3_^-^  115.11 |  |
|  | 3-acetoxy-2-methylenebutyrate  [3OAcMB]^-^ | C_7_H_9_O_4_^-^  157.15 |  |
|  | Abietate  [Abt]^-^ | C_20_H_29_O_2_^-^  301.45 |  |
|  | Acetate  [Ace]^-^ | C_2_H_3_O_2_^-^  59.04 |  |
|  | Acetylsalicylate  [AceSal]^-^ | C_9_H_7_O_4_^-^  179.15 |  |
|  | Acrylate  [Acryl]^-^ | C_3_H_3_O_2_^-^  71.06 |  |
|  | Benzoate  [Ben]^-^ | C_7_H_5_O_2_^-^  121.12 |  |
|  | Butyrate  [But]^-^ | C_4_H_7_O_2_^-^  87.10 |  |
|  | Valerate  [Valerate]^-^ | C_5_H_9_O_2_^-^  101.13 |  |
|  | Heptanoate  [Hep]^-^ | C_7_H_13_O_2_^-^  129.18 |  |
|  | Coumarine-3-carboxylate  [C3C]^-^ | C_10_H_5_O_4_^-^  189.15 |  |
|  | Crotonate  [Crotonate]^-^ | C_4_H_5_O_2_^-^  85.08 |  |
|  | Decanoate  [Dec]^-^ | C_10_H_19_O_2_^-^  171.26 |  |
|  | Formate  [For]^-^ | CHO_2_^-^  45.02 |  |
|  | Gentisate  [Gen]^-^ | C_7_H_5_O_4_^-^  153.11 |  |
|  | Glycolate  [Glyco]^-^ | C_2_H_3_O_3_^-^  75.04 |  |
|  | Hexanoate  [Hex]^-^ | C_6_H_11_O_2_^-^  115.15 |  |
|  | Imidazole-2-carboxylate  [Im2C]^-^ | C_4_H_3_N_2_O_2_^-^  111.08 |  |
|  | Indole-3-acetate  [I3A]^-^ | C_10_H_8_NO_2_^-^  174.18 |  |
|  | Lactate  [Lac]^-^ | C_3_H_5_O_3_^-^  89.07 |  |
|  | Methyl carbonate  [MCb]^-^ | C_2_H_3_O_3_^-^  75.04 |  |
|  | N-methylcarbamate  [N-MCb]^-^ | C_2_H_4_NO_2_^-^  74.06 |  |
|  | Octanoate  [Oct]^-^ | C_8_H_15_O_2_^-^  143.21 |  |
|  | Propionate  [Prop]^-^ | C_3_H_5_O_2_^-^  73.07 |  |
|  | Thiosalicylate  [ThioSal]^-^ | C_7_H_5_O_2_S^-^  153.18 |  |
|  | 2-(trifluoromethyl)acrylate  [2TFMAcr]- | C_4_H_2_F_3_O_2_^-^  139.05 |  |
|  | Phenolate  [Phenolate]^-^ | C_6_H_5_O^-^  93.11 |  |
|  | Dibutyl phosphate  [DBPO_4_]^-^ | C_8_H_18_O_4_P^-^  209.20 |  |
|  | Diethylphosphate  [DEPO_4_]^-^ | C_4_H_10_O_4_P^-^  153.09 |  |
|  | Diisopropyl phosphate  [DIsoPrPO_4_]^-^ | C_6_H_14_O_4_P^-^  181.15 |  |
|  | Dimethylphosphate  [DMPO_4_]^-^ | C_2_H_6_O_4_P^-^  125.04 |  |
|  | Ethyl sulfate  [EtSO_4_]^-^ | C_2_H_5_O_4_S^-^  125.12 |  |
|  | 4-(diethylamino)butane-1-sulfonate  [DEABSO_3_]^-^ | C_8_H_18_NO_3_S^-^  208.30 |  |
|  | 3-(isopropylamino)propane-1-sulfonate  [IsoPAPSO_3_]^-^ | C_6_H_14_NO_3_S^-^  180.24 |  |
|  | 3-(diethylamino)propane-1-sulfonate  [DEAPSO_3_]^-^ | C_7_H_16_NO_3_S^-^  194.27 |  |
|  | 4-(isopropylamino)butane-1-sulfonate  [IsoPABSO_3_]^-^ | C_7_H_16_NO_3_S^-^  194.27 |  |
|  | 4-(dimethylamino)butane-1-sulfonate  [DMABSO_3_]^-^ | C_6_H_14_NO_3_S^-^  180.24 |  |
|  | 3-(butylamino)propane-1-sulfonate  [BAPSO_3_]^-^ | C_7_H_16_NO_3_S^-^  194.27 |  |
|  | 3-(hexylamino)propane-1-sulfonate  [HAPSO_3_]^-^ | C_9_H_20_NO_3_S^-^  222.32 |  |
|  | Methyl sulfate  [MeSO_4_]^-^ | CH_3_O_4_S^-^  111.09 |  |
|  | Methylsulfonate  [MeSO_3_]^-^ | CH_3_O_3_S^-^  95.09 |  |
|  | Xylenesulfonate  [XylSO_3_]^-^ | C_7_H_7_O_3_S^-^  171.19 |  |
|  | Octylsulfate  [OctSO_4_]^-^ | C_8_H_17_O_4_S^-^  209.28 |  |
|  | Taurinate  [Tau]^-^ | C_2_H_6_NO_3_S^-^  124.13 |  |
|  | 2-ethylimidazol-1-ide  [2EIm]^-^ | C_5_H_7_N_2_^-^  95.13 |  |
|  | N,N-dimethyloxamate  [DMeO]^-^ | C_4_H_6_NO_3_^-^  116.10 |  |
|  | Dihydrogen phosphite  [H_2_PO_3_]^-^ | H_2_O_3_P^-^  80.99 |  |
|  | Dihydrogen phosphate  [H_2_PO_4_]^-^ | H_2_O_4_P^-^  96.99 |  |
|  | Ethyl methylphosphonate  [EMPO_3_]^-^ | C_3_H_8_O_3_P^-^  123.07 |  |
|  | Isopropyl methylphosphonate  [IsoPMPO_3_]^-^ | C4H10O3P-  137.10 |  |
|  | Indazolate  [INDz]^-^ | C_8_H_5_N_2_O_2_^-^  161.14 |  |
|  | 5-aminotetrazol-1-ide  [5ATeAz]^-^ | CH_2_N_5_^-^  84.06 |  |
|  | Benzo[d][1,2,3]triazol-2-ide  [Be123TAz]^-^ | C_6_H_4_N_3_^-^  118.12 |  |
|  | Tetrazol-1-ide  [TeAz]- | CHN_4_^-^  69.05 |  |
|  | 1,2,4-triazol-1-ide  [124TAz]^-^ | C_2_H_2_N_3_^-^  68.06 |  |
|  | 2,5-dioxopyrrolidin-1-ide  [DOPyrr]^-^ | C4H4NO2-  98.08 |  |
|  | N-methyloxamate  [NMO]^-^ | C_3_H_4_NO_3_^-^  102.07 |  |
|  | Imidazolate  [IMZ]^-^ | C_4_H_3_N_2_O_2_^-^  111.08 |  |

**Table S3:** COSMO-RS predicted logarithmic activity coefficients (ln(*γ*)) of lignin in different ILs at 363.15 K

| Anion /Cation | [TBA]+ | [TBP]+ | [DPrPyrr]+ | [TEP]+ | [TBMP]+ | [MTOA]+ | [TEA]+ | [BDMIM]^+^ | [BMPyrr]+ | [THTeDP]+ | [TEMP]+ | [EMPyrr]+ |
| --- | --- | --- | --- | --- | --- | --- | --- | --- | --- | --- | --- | --- |
| [Ace]- | -7.11 | -7.07 | -6.78 | -6.65 | -6.58 | -6.51 | -6.43 | -6.25 | -6.25 | -6.24 | -6.16 | -5.91 |
| [Prop]- | -6.88 | -6.83 | -6.61 | -6.48 | -6.39 | -6.26 | -6.29 | -6.12 | -6.12 | -5.95 | -6.02 | -5.81 |
| [But]- | -6.81 | -6.76 | -6.59 | -6.47 | -6.34 | -6.17 | -6.29 | -6.12 | -6.12 | -5.85 | -6.03 | -5.84 |
| [Valerate]- | -6.74 | -6.69 | -6.57 | -6.45 | -6.29 | -6.08 | -6.29 | -6.11 | -6.12 | -5.75 | -6.03 | -5.86 |
| [N-MCb]- | -6.74 | -6.71 | -6.39 | -6.27 | -6.22 | -6.16 | -6.04 | -5.87 | -5.87 | -5.91 | -5.79 | -5.52 |
| [Hexa]- | -6.70 | -6.64 | -6.57 | -6.46 | -6.27 | -6.02 | -6.31 | -6.13 | -6.14 | -5.67 | -6.05 | -5.91 |
| [Hep]^-^ | -6.65 | -6.58 | -6.56 | -6.46 | -6.23 | -5.95 | -6.32 | -6.14 | -6.15 | -5.59 | -6.06 | -5.94 |
| [Oct]- | -6.60 | -6.54 | -6.56 | -6.46 | -6.21 | -5.89 | -6.33 | -6.15 | -6.16 | -5.52 | -6.08 | -5.97 |
| [Gly]- | -6.53 | -6.50 | -6.15 | -6.04 | -6.02 | -5.98 | -5.81 | -5.66 | -5.64 | -5.73 | -5.56 | -5.28 |
| [Dec]- | -6.49 | -6.42 | -6.51 | -6.42 | -6.12 | -5.75 | -6.32 | -6.13 | -6.15 | -5.35 | -6.06 | -6.00 |
| [Crotonate]- | -6.35 | -6.31 | -6.07 | -5.95 | -5.89 | -5.76 | -5.76 | -5.60 | -5.60 | -5.46 | -5.52 | -5.29 |
| [Pro]- | -6.33 | -6.29 | -6.10 | -6.00 | -5.89 | -5.71 | -5.81 | -5.65 | -5.64 | -5.40 | -5.57 | -5.36 |
| [Ala]- | -6.30 | -6.27 | -6.00 | -5.88 | -5.84 | -5.73 | -5.68 | -5.54 | -5.52 | -5.44 | -5.44 | -5.20 |
| [Lys]- | -6.29 | -6.25 | -6.03 | -5.93 | -5.86 | -5.73 | -5.75 | -5.61 | -5.59 | -5.46 | -5.53 | -5.31 |
| [For]- | -6.14 | -6.11 | -5.73 | -5.61 | -5.63 | -5.60 | -5.37 | -5.23 | -5.20 | -5.34 | -5.13 | -4.82 |
| [Lac]- | -6.08 | -6.06 | -5.69 | -5.57 | -5.58 | -5.60 | -5.35 | -5.21 | -5.18 | -5.39 | -5.10 | -4.83 |
| [2OZ]- | -6.02 | -5.98 | -5.70 | -5.59 | -5.56 | -5.45 | -5.38 | -5.23 | -5.22 | -5.16 | -5.16 | -4.89 |
| [PyZ]- | -5.98 | -5.94 | -5.68 | -5.56 | -5.57 | -5.44 | -5.36 | -5.22 | -5.25 | -5.11 | -5.16 | -4.92 |
| [Glyco]- | -5.94 | -5.92 | -5.47 | -5.35 | -5.41 | -5.50 | -5.10 | -4.98 | -4.94 | -5.32 | -4.86 | -4.55 |
| [EMPO_3_]^-^ | -5.92 | -5.88 | -5.68 | -5.59 | -5.49 | -5.31 | -5.40 | -5.25 | -5.23 | -5.01 | -5.17 | -4.95 |
| [Isole]- | -5.84 | -5.79 | -5.69 | -5.59 | -5.46 | -5.22 | -5.44 | -5.30 | -5.29 | -4.86 | -5.22 | -5.05 |
| [Acryl]- | -5.83 | -5.80 | -5.51 | -5.40 | -5.39 | -5.28 | -5.19 | -5.06 | -5.05 | -4.98 | -4.97 | -4.71 |
| [IsoPMPO_3_]^-^ | -5.77 | -5.73 | -5.59 | -5.50 | -5.38 | -5.15 | -5.33 | -5.20 | -5.18 | -4.81 | -5.11 | -4.92 |
| [Arg]- | -5.74 | -5.74 | -5.29 | -5.17 | -5.29 | -5.42 | -4.95 | -4.87 | -4.84 | -5.27 | -4.76 | -4.48 |
| [Val]- | -5.60 | -5.56 | -5.41 | -5.31 | -5.22 | -5.01 | -5.14 | -5.02 | -5.01 | -4.66 | -4.93 | -4.74 |
| [Abt]- | -5.44 | -5.36 | -5.60 | -5.52 | -5.20 | -4.71 | -5.48 | -5.31 | -5.34 | -4.23 | -5.26 | -5.27 |
| [IMCrb]- | -5.34 | -5.32 | -4.92 | -4.80 | -4.90 | -4.92 | -4.57 | -4.46 | -4.46 | -4.69 | -4.38 | -4.09 |
| [Leu]- | -5.33 | -5.28 | -5.20 | -5.11 | -4.99 | -4.72 | -4.97 | -4.84 | -4.83 | -4.35 | -4.76 | -4.60 |
| [124TAz]- | -5.27 | -5.25 | -4.90 | -4.79 | -4.86 | -4.78 | -4.57 | -4.45 | -4.47 | -4.48 | -4.39 | -4.10 |
| [Met]- | -5.25 | -5.21 | -5.02 | -4.92 | -4.88 | -4.71 | -4.75 | -4.62 | -4.63 | -4.38 | -4.56 | -4.35 |
| [2AIm]- | -5.15 | -5.13 | -4.75 | -4.65 | -4.76 | -4.71 | -4.43 | -4.33 | -4.32 | -4.43 | -4.26 | -3.96 |
| [Ben]- | -5.00 | -4.96 | -4.76 | -4.66 | -4.65 | -4.47 | -4.49 | -4.38 | -4.39 | -4.12 | -4.31 | -4.10 |
| [DOPyrr]^-^ | -4.95 | -4.92 | -4.61 | -4.53 | -4.54 | -4.42 | -4.31 | -4.19 | -4.16 | -4.13 | -4.12 | -3.83 |
| [Glu]- | -4.93 | -4.92 | -4.48 | -4.39 | -4.50 | -4.58 | -4.16 | -4.10 | -4.04 | -4.38 | -3.98 | -3.68 |
| [MCb]^-^ | -4.85 | -4.82 | -4.48 | -4.40 | -4.42 | -4.33 | -4.17 | -4.07 | -4.02 | -4.04 | -3.98 | -3.67 |
| [NMO]- | -4.83 | -4.81 | -4.48 | -4.39 | -4.42 | -4.33 | -4.18 | -4.07 | -4.03 | -4.05 | -3.99 | -3.70 |
| [2MIm]- | -4.83 | -4.79 | -4.59 | -4.50 | -4.52 | -4.31 | -4.32 | -4.23 | -4.23 | -3.92 | -4.17 | -3.94 |
| [Cyst]- | -4.82 | -4.79 | -4.49 | -4.39 | -4.45 | -4.35 | -4.19 | -4.09 | -4.09 | -4.04 | -4.02 | -3.76 |
| [Phe]- | -4.75 | -4.70 | -4.57 | -4.48 | -4.43 | -4.20 | -4.33 | -4.22 | -4.22 | -3.84 | -4.16 | -3.98 |
| [IMZ]- | -4.74 | -4.71 | -4.43 | -4.33 | -4.40 | -4.25 | -4.13 | -4.05 | -4.05 | -3.90 | -3.98 | -3.71 |
| [Thr]- | -4.72 | -4.69 | -4.42 | -4.34 | -4.35 | -4.20 | -4.15 | -4.07 | -4.02 | -3.89 | -3.97 | -3.71 |
| [I3A]- | -4.70 | -4.68 | -4.43 | -4.32 | -4.38 | -4.31 | -4.16 | -4.08 | -4.09 | -4.00 | -4.00 | -3.81 |
| [His]- | -4.67 | -4.66 | -4.23 | -4.12 | -4.27 | -4.37 | -3.91 | -3.86 | -3.83 | -4.15 | -3.75 | -3.47 |
| [3OAcMB]^-^ | -4.61 | -4.57 | -4.38 | -4.30 | -4.27 | -4.08 | -4.13 | -4.03 | -4.00 | -3.74 | -3.96 | -3.73 |
| [Phenolate]- | -4.55 | -4.51 | -4.35 | -4.25 | -4.26 | -4.03 | -4.10 | -4.02 | -4.02 | -3.62 | -3.94 | -3.74 |
| [DEPO_4_]- | -4.51 | -4.47 | -4.31 | -4.25 | -4.17 | -3.92 | -4.08 | -3.97 | -3.93 | -3.58 | -3.90 | -3.68 |
| [Ser]- | -4.49 | -4.48 | -4.07 | -3.97 | -4.08 | -4.11 | -3.75 | -3.69 | -3.63 | -3.88 | -3.57 | -3.26 |
| [2EIm]- | -4.49 | -4.45 | -4.33 | -4.24 | -4.23 | -3.95 | -4.09 | -4.01 | -4.01 | -3.54 | -3.95 | -3.75 |
| [DMO]- | -4.47 | -4.45 | -4.13 | -4.06 | -4.08 | -3.97 | -3.84 | -3.74 | -3.69 | -3.69 | -3.67 | -3.37 |
| [H2PO_3_]- | -4.42 | -4.41 | -3.96 | -3.87 | -4.01 | -4.03 | -3.63 | -3.59 | -3.51 | -3.78 | -3.46 | -3.12 |
| [Aspg]- | -4.40 | -4.41 | -3.87 | -3.77 | -3.97 | -4.13 | -3.52 | -3.49 | -3.42 | -3.97 | -3.36 | -3.02 |
| [DIsoPrPO_4_]- | -4.37 | -4.32 | -4.27 | -4.21 | -4.07 | -3.75 | -4.07 | -3.96 | -3.93 | -3.37 | -3.90 | -3.72 |
| [DMPO_4_]^-^ | -4.33 | -4.31 | -4.03 | -3.96 | -3.97 | -3.82 | -3.76 | -3.67 | -3.62 | -3.51 | -3.59 | -3.31 |
| [DBPO_4_]^-^ | -4.26 | -4.21 | -4.24 | -4.19 | -4.00 | -3.61 | -4.08 | -3.96 | -3.93 | -3.21 | -3.91 | -3.77 |
| [3-(OH)2-MB]^-^ | -4.23 | -4.21 | -3.93 | -3.84 | -3.89 | -3.81 | -3.66 | -3.60 | -3.56 | -3.49 | -3.49 | -3.25 |
| [ClAce]- | -4.22 | -4.20 | -3.89 | -3.81 | -3.87 | -3.74 | -3.60 | -3.54 | -3.49 | -3.42 | -3.44 | -3.16 |
| [ThioSal]- | -4.19 | -4.16 | -3.93 | -3.83 | -3.88 | -3.73 | -3.66 | -3.57 | -3.59 | -3.38 | -3.52 | -3.30 |
| [INDz]- | -4.18 | -4.14 | -3.98 | -3.89 | -3.91 | -3.68 | -3.73 | -3.64 | -3.67 | -3.27 | -3.60 | -3.40 |
| [Try]- | -4.18 | -4.15 | -3.93 | -3.83 | -3.89 | -3.79 | -3.69 | -3.62 | -3.63 | -3.48 | -3.54 | -3.36 |
| [2ClMAcr]- | -4.12 | -4.09 | -3.93 | -3.84 | -3.83 | -3.60 | -3.69 | -3.61 | -3.59 | -3.20 | -3.53 | -3.32 |
| [2BrMAcr]- | -4.01 | -3.97 | -3.79 | -3.69 | -3.72 | -3.50 | -3.53 | -3.43 | -3.47 | -3.11 | -3.39 | -3.19 |
| [2OHMAcr]- | -4.01 | -3.98 | -3.73 | -3.65 | -3.69 | -3.51 | -3.47 | -3.40 | -3.36 | -3.15 | -3.31 | -3.06 |
| [5ATeAz]- | -3.90 | -3.91 | -3.34 | -3.24 | -3.49 | -3.60 | -2.97 | -2.91 | -2.89 | -3.40 | -2.83 | -2.45 |
| [C3C]- | -3.79 | -3.77 | -3.50 | -3.43 | -3.48 | -3.36 | -3.25 | -3.17 | -3.15 | -3.06 | -3.11 | -2.86 |
| [DEABSO_3_]- | -3.73 | -3.69 | -3.65 | -3.62 | -3.47 | -3.11 | -3.48 | -3.38 | -3.33 | -2.74 | -3.33 | -3.15 |
| [Be123TAz]- | -3.73 | -3.70 | -3.46 | -3.37 | -3.45 | -3.25 | -3.20 | -3.11 | -3.13 | -2.89 | -3.07 | -2.83 |
| [IsoPAPSO_3_]- | -3.72 | -3.69 | -3.55 | -3.52 | -3.43 | -3.16 | -3.35 | -3.26 | -3.20 | -2.82 | -3.21 | -2.98 |
| [AceSal]- | -3.72 | -3.69 | -3.50 | -3.43 | -3.45 | -3.24 | -3.27 | -3.20 | -3.18 | -2.88 | -3.14 | -2.92 |
| [DEAPSO_3_]- | -3.70 | -3.67 | -3.59 | -3.55 | -3.44 | -3.11 | -3.40 | -3.31 | -3.25 | -2.74 | -3.25 | -3.05 |
| [56DMBeIm]- | -3.68 | -3.64 | -3.58 | -3.51 | -3.49 | -3.16 | -3.39 | -3.31 | -3.33 | -2.71 | -3.28 | -3.11 |
| [TeAz]- | -3.67 | -3.66 | -3.23 | -3.14 | -3.31 | -3.25 | -2.90 | -2.82 | -2.82 | -2.95 | -2.77 | -2.43 |
| [IsoPABSO_3_]- | -3.66 | -3.63 | -3.45 | -3.41 | -3.36 | -3.12 | -3.24 | -3.16 | -3.09 | -2.79 | -3.09 | -2.85 |
| [DMABSO_3_]- | -3.64 | -3.61 | -3.46 | -3.42 | -3.35 | -3.07 | -3.25 | -3.16 | -3.10 | -2.73 | -3.10 | -2.87 |
| [BAPSO_3_]- | -3.61 | -3.58 | -3.46 | -3.42 | -3.33 | -3.04 | -3.26 | -3.18 | -3.12 | -2.69 | -3.12 | -2.90 |
| [2TFMAcr]- | -3.59 | -3.56 | -3.40 | -3.33 | -3.34 | -3.09 | -3.18 | -3.13 | -3.10 | -2.67 | -3.04 | -2.83 |
| [Tau]- | -3.58 | -3.59 | -3.12 | -3.05 | -3.20 | -3.21 | -2.80 | -2.77 | -2.68 | -2.99 | -2.66 | -2.30 |
| [MeSO_3_]- | -3.55 | -3.54 | -3.12 | -3.06 | -3.16 | -3.10 | -2.82 | -2.76 | -2.68 | -2.83 | -2.67 | -2.31 |
| [HAPSO_3_]- | -3.55 | -3.51 | -3.47 | -3.45 | -3.30 | -2.94 | -3.31 | -3.22 | -3.17 | -2.57 | -3.17 | -3.00 |
| [GluMA]- | -3.47 | -3.46 | -3.13 | -3.05 | -3.14 | -3.10 | -2.86 | -2.82 | -2.77 | -2.83 | -2.71 | -2.46 |
| [Pur]- | -3.42 | -3.40 | -3.16 | -3.07 | -3.17 | -2.96 | -2.89 | -2.82 | -2.85 | -2.58 | -2.79 | -2.54 |
| [BeIm]- | -3.35 | -3.32 | -3.16 | -3.08 | -3.15 | -2.88 | -2.93 | -2.88 | -2.89 | -2.45 | -2.84 | -2.62 |
| [Tyro]- | -3.28 | -3.26 | -3.00 | -2.91 | -3.00 | -2.93 | -2.76 | -2.72 | -2.69 | -2.63 | -2.62 | -2.41 |
| [AspMA]- | -3.25 | -3.24 | -2.86 | -2.77 | -2.91 | -2.92 | -2.57 | -2.54 | -2.48 | -2.65 | -2.43 | -2.14 |
| [H2PO_4_]- | -3.05 | -3.06 | -2.48 | -2.40 | -2.63 | -2.79 | -2.14 | -2.13 | -2.03 | -2.61 | -1.99 | -1.60 |
| [XylSO_3_]- | -2.53 | -2.50 | -2.34 | -2.30 | -2.31 | -2.04 | -2.14 | -2.09 | -2.05 | -1.65 | -2.04 | -1.81 |
| [Gen]- | -2.42 | -2.43 | -1.90 | -1.79 | -2.07 | -2.28 | -1.57 | -1.58 | -1.53 | -2.11 | -1.45 | -1.14 |
| [2NIm]- | -2.34 | -2.32 | -2.06 | -2.00 | -2.14 | -1.93 | -1.82 | -1.81 | -1.78 | -1.52 | -1.75 | -1.48 |
| [EtSO_4_]- | -1.89 | -1.88 | -1.57 | -1.54 | -1.66 | -1.47 | -1.34 | -1.34 | -1.25 | -1.10 | -1.26 | -0.94 |
| [MeSO_4_]- | -1.83 | -1.83 | -1.45 | -1.41 | -1.58 | -1.45 | -1.19 | -1.20 | -1.10 | -1.11 | -1.10 | -0.76 |
| [OMeSO_4_]- | -1.73 | -1.69 | -1.73 | -1.73 | -1.61 | -1.15 | -1.62 | -1.58 | -1.52 | -0.69 | -1.54 | -1.38 |

**Table S3 (continued…):**  COSMO-RS predicted logarithmic activity coefficients (ln(*γ*)) of lignin in different ILs at 363.15 K

| Anion /Cation | [TEMA]+ | [BeTPhP]+ | [EDMIM]+ | [HMG]+ | [ADMPy]+ | [TMBP]+ | [EDMPrA]+ | [DEM-2-MeoEA]^+^ | [BeBIM]+ | [B3MPy]+ |
| --- | --- | --- | --- | --- | --- | --- | --- | --- | --- | --- |
| [Ace]- | -5.89 | -5.79 | -5.74 | -5.73 | -5.68 | -5.59 | -5.56 | -5.56 | -5.48 | -5.41 |
| [Prop]- | -5.78 | -5.64 | -5.65 | -5.63 | -5.57 | -5.50 | -5.48 | -5.47 | -5.37 | -5.31 |
| [But]- | -5.81 | -5.61 | -5.68 | -5.65 | -5.58 | -5.51 | -5.50 | -5.50 | -5.36 | -5.32 |
| [Valerate]- | -5.83 | -5.59 | -5.70 | -5.67 | -5.59 | -5.52 | -5.53 | -5.52 | -5.36 | -5.33 |
| [N-MCb]- | -5.51 | -5.47 | -5.37 | -5.36 | -5.34 | -5.24 | -5.19 | -5.19 | -5.16 | -5.09 |
| [Hexa]- | -5.87 | -5.58 | -5.75 | -5.71 | -5.62 | -5.56 | -5.57 | -5.57 | -5.37 | -5.35 |
| [Hep]^-^ | -5.89 | -5.57 | -5.78 | -5.73 | -5.64 | -5.57 | -5.60 | -5.59 | -5.38 | -5.37 |
| [Oct]- | -5.92 | -5.56 | -5.81 | -5.76 | -5.65 | -5.60 | -5.64 | -5.63 | -5.38 | -5.38 |
| [Gly]- | -5.28 | -5.31 | -5.15 | -5.14 | -5.14 | -5.03 | -4.98 | -4.97 | -4.99 | -4.90 |
| [Dec]- | -5.94 | -5.51 | -5.83 | -5.77 | -5.65 | -5.60 | -5.66 | -5.65 | -5.36 | -5.38 |
| [Crotonate]- | -5.27 | -5.15 | -5.14 | -5.13 | -5.09 | -5.02 | -4.99 | -4.98 | -4.91 | -4.86 |
| [Pro]- | -5.34 | -5.22 | -5.22 | -5.20 | -5.17 | -5.07 | -5.05 | -5.04 | -4.96 | -4.92 |
| [Ala]- | -5.19 | -5.16 | -5.07 | -5.06 | -5.04 | -4.94 | -4.90 | -4.90 | -4.88 | -4.81 |
| [Lys]- | -5.29 | -5.26 | -5.19 | -5.17 | -5.16 | -5.05 | -5.02 | -5.01 | -4.99 | -4.92 |
| [For]- | -4.83 | -4.89 | -4.70 | -4.70 | -4.71 | -4.60 | -4.53 | -4.53 | -4.57 | -4.49 |
| [Lac]- | -4.82 | -4.92 | -4.71 | -4.71 | -4.70 | -4.58 | -4.53 | -4.55 | -4.57 | -4.47 |
| [2OZ]- | -4.89 | -4.86 | -4.76 | -4.76 | -4.75 | -4.66 | -4.61 | -4.60 | -4.59 | -4.54 |
| [PyZ]- | -4.91 | -4.82 | -4.76 | -4.77 | -4.73 | -4.72 | -4.66 | -4.64 | -4.62 | -4.54 |
| [Glyco]- | -4.56 | -4.74 | -4.44 | -4.45 | -4.46 | -4.33 | -4.26 | -4.29 | -4.35 | -4.23 |
| [EMPO_3_]^-^ | -4.93 | -4.84 | -4.83 | -4.81 | -4.79 | -4.68 | -4.65 | -4.65 | -4.59 | -4.56 |
| [Isole]- | -5.03 | -4.86 | -4.93 | -4.90 | -4.85 | -4.78 | -4.77 | -4.76 | -4.67 | -4.63 |
| [Acryl]- | -4.71 | -4.68 | -4.58 | -4.58 | -4.57 | -4.49 | -4.44 | -4.43 | -4.43 | -4.36 |
| [IsoPMPO_3_]^-^ | -4.90 | -4.77 | -4.81 | -4.78 | -4.75 | -4.65 | -4.64 | -4.63 | -4.55 | -4.52 |
| [Arg]- | -4.49 | -4.74 | -4.39 | -4.41 | -4.43 | -4.34 | -4.25 | -4.25 | -4.40 | -4.25 |
| [Val]- | -4.73 | -4.62 | -4.63 | -4.61 | -4.58 | -4.50 | -4.48 | -4.47 | -4.42 | -4.37 |
| [Abt]- | -5.20 | -4.68 | -5.12 | -5.06 | -4.92 | -4.91 | -4.98 | -4.97 | -4.65 | -4.68 |
| [IMCrb]- | -4.10 | -4.21 | -3.97 | -4.00 | -4.01 | -3.94 | -3.86 | -3.85 | -3.94 | -3.84 |
| [Leu]- | -4.58 | -4.42 | -4.49 | -4.47 | -4.43 | -4.36 | -4.35 | -4.33 | -4.26 | -4.22 |
| [124TAz]- | -4.11 | -4.16 | -3.97 | -3.99 | -4.00 | -3.97 | -3.88 | -3.86 | -3.94 | -3.83 |
| [Met]- | -4.34 | -4.25 | -4.23 | -4.23 | -4.21 | -4.15 | -4.11 | -4.09 | -4.07 | -4.03 |
| [2AIm]- | -3.98 | -4.12 | -3.86 | -3.88 | -3.92 | -3.86 | -3.76 | -3.74 | -3.88 | -3.76 |
| [Ben]- | -4.10 | -3.99 | -3.98 | -3.98 | -3.96 | -3.93 | -3.88 | -3.86 | -3.84 | -3.80 |
| [DOPyrr]^-^ | -3.85 | -3.91 | -3.74 | -3.75 | -3.78 | -3.67 | -3.60 | -3.59 | -3.66 | -3.60 |
| [Glu]- | -3.71 | -3.97 | -3.62 | -3.64 | -3.69 | -3.57 | -3.48 | -3.48 | -3.65 | -3.53 |
| [MCb]^-^ | -3.70 | -3.81 | -3.60 | -3.60 | -3.65 | -3.52 | -3.44 | -3.44 | -3.53 | -3.47 |
| [NMO]- | -3.72 | -3.79 | -3.61 | -3.63 | -3.66 | -3.54 | -3.47 | -3.46 | -3.54 | -3.49 |
| [2MIm]- | -3.95 | -3.92 | -3.84 | -3.85 | -3.85 | -3.82 | -3.75 | -3.73 | -3.78 | -3.70 |
| [Cyst]- | -3.77 | -3.81 | -3.65 | -3.68 | -3.68 | -3.63 | -3.55 | -3.54 | -3.61 | -3.53 |
| [Phe]- | -3.97 | -3.85 | -3.87 | -3.86 | -3.84 | -3.80 | -3.76 | -3.74 | -3.72 | -3.68 |
| [IMZ]- | -3.73 | -3.78 | -3.61 | -3.64 | -3.65 | -3.62 | -3.53 | -3.52 | -3.61 | -3.52 |
| [Thr]- | -3.73 | -3.82 | -3.65 | -3.65 | -3.69 | -3.57 | -3.50 | -3.49 | -3.58 | -3.52 |
| [I3A]- | -3.81 | -3.80 | -3.70 | -3.72 | -3.69 | -3.68 | -3.62 | -3.61 | -3.65 | -3.56 |
| [His]- | -3.49 | -3.72 | -3.39 | -3.43 | -3.45 | -3.38 | -3.29 | -3.29 | -3.45 | -3.31 |
| [3OAcMB]^-^ | -3.74 | -3.70 | -3.65 | -3.65 | -3.66 | -3.57 | -3.52 | -3.51 | -3.52 | -3.50 |
| [Phenolate]- | -3.75 | -3.66 | -3.64 | -3.65 | -3.63 | -3.61 | -3.56 | -3.54 | -3.56 | -3.50 |
| [DEPO_4_]- | -3.68 | -3.64 | -3.61 | -3.59 | -3.61 | -3.49 | -3.45 | -3.44 | -3.45 | -3.44 |
| [Ser]- | -3.29 | -3.57 | -3.21 | -3.23 | -3.29 | -3.15 | -3.06 | -3.08 | -3.25 | -3.12 |
| [2EIm]- | -3.76 | -3.68 | -3.66 | -3.66 | -3.66 | -3.64 | -3.58 | -3.56 | -3.58 | -3.52 |
| [DMO]- | -3.39 | -3.49 | -3.31 | -3.31 | -3.37 | -3.23 | -3.16 | -3.14 | -3.24 | -3.21 |
| [H2PO_3_]- | -3.16 | -3.49 | -3.09 | -3.11 | -3.20 | -3.04 | -2.93 | -2.95 | -3.17 | -3.05 |
| [Aspg]- | -3.06 | -3.46 | -2.98 | -3.02 | -3.10 | -2.97 | -2.85 | -2.85 | -3.11 | -2.96 |
| [DIsoPrPO_4_]- | -3.72 | -3.59 | -3.65 | -3.62 | -3.62 | -3.51 | -3.50 | -3.49 | -3.44 | -3.44 |
| [DMPO_4_]^-^ | -3.33 | -3.42 | -3.26 | -3.25 | -3.31 | -3.17 | -3.11 | -3.10 | -3.19 | -3.15 |
| [DBPO_4_]^-^ | -3.76 | -3.55 | -3.70 | -3.66 | -3.64 | -3.54 | -3.55 | -3.53 | -3.43 | -3.46 |
| [3-(OH)2-MB]^-^ | -3.27 | -3.38 | -3.19 | -3.20 | -3.22 | -3.14 | -3.07 | -3.07 | -3.17 | -3.07 |
| [ClAce]- | -3.18 | -3.32 | -3.11 | -3.11 | -3.17 | -3.05 | -2.97 | -2.96 | -3.10 | -3.03 |
| [ThioSal]- | -3.31 | -3.24 | -3.18 | -3.21 | -3.19 | -3.20 | -3.13 | -3.10 | -3.14 | -3.09 |
| [INDz]- | -3.41 | -3.32 | -3.29 | -3.31 | -3.29 | -3.31 | -3.24 | -3.21 | -3.25 | -3.19 |
| [Try]- | -3.36 | -3.38 | -3.27 | -3.29 | -3.27 | -3.26 | -3.20 | -3.18 | -3.24 | -3.16 |
| [2ClMAcr]- | -3.33 | -3.30 | -3.25 | -3.25 | -3.26 | -3.20 | -3.15 | -3.13 | -3.17 | -3.12 |
| [2BrMAcr]- | -3.19 | -3.06 | -3.05 | -3.09 | -3.05 | -3.08 | -3.01 | -2.99 | -2.99 | -2.95 |
| [2OHMAcr]- | -3.08 | -3.15 | -3.00 | -3.01 | -3.05 | -2.96 | -2.88 | -2.87 | -2.97 | -2.92 |
| [5ATeAz]- | -2.50 | -2.88 | -2.37 | -2.44 | -2.52 | -2.45 | -2.29 | -2.28 | -2.58 | -2.41 |
| [C3C]- | -2.88 | -2.91 | -2.79 | -2.80 | -2.83 | -2.77 | -2.70 | -2.68 | -2.75 | -2.73 |
| [DEABSO_3_]- | -3.15 | -3.06 | -3.11 | -3.080 | -3.10 | -2.97 | -2.96 | -2.94 | -2.92 | -2.94 |
| [Be123TAz]- | -2.85 | -2.85 | -2.73 | -2.76 | -2.77 | -2.78 | -2.68 | -2.65 | -2.75 | -2.68 |
| [IsoPAPSO_3_]- | -3.00 | -3.01 | -2.95 | -2.930 | -2.98 | -2.83 | -2.80 | -2.78 | -2.82 | -2.82 |
| [AceSal]- | -2.93 | -2.91 | -2.85 | -2.86 | -2.88 | -2.82 | -2.76 | -2.74 | -2.79 | -2.77 |
| [DEAPSO_3_]- | -3.06 | -3.01 | -3.01 | -2.991 | -3.02 | -2.89 | -2.86 | -2.84 | -2.86 | -2.87 |
| [56DMBeIm]- | -3.12 | -2.99 | -3.02 | -3.03 | -3.02 | -3.03 | -2.98 | -2.95 | -2.96 | -2.93 |
| [TeAz]- | -2.47 | -2.69 | -2.34 | -2.40 | -2.46 | -2.41 | -2.28 | -2.26 | -2.47 | -2.36 |
| [IsoPABSO_3_]- | -2.87 | -2.93 | -2.82 | -2.809 | -2.87 | -2.72 | -2.67 | -2.65 | -2.73 | -2.72 |
| [DMABSO_3_]- | -2.89 | -2.90 | -2.84 | -2.820 | -2.87 | -2.73 | -2.68 | -2.67 | -2.72 | -2.72 |
| [BAPSO_3_]- | -2.92 | -2.92 | -2.87 | -2.852 | -2.90 | -2.75 | -2.72 | -2.70 | -2.74 | -2.75 |
| [2TFMAcr]- | -2.85 | -2.86 | -2.79 | -2.79 | -2.82 | -2.74 | -2.69 | -2.66 | -2.75 | -2.71 |
| [Tau]- | -2.35 | -2.72 | -2.29 | -2.319 | -2.44 | -2.26 | -2.15 | -2.14 | -2.41 | -2.31 |
| [MeSO_3_]- | -2.36 | -2.64 | -2.29 | -2.314 | -2.42 | -2.25 | -2.14 | -2.14 | -2.36 | -2.30 |
| [HAPSO_3_]- | -3.00 | -2.92 | -2.96 | -2.932 | -2.96 | -2.82 | -2.81 | -2.79 | -2.78 | -2.80 |
| [GluMA]- | -2.48 | -2.71 | -2.42 | -2.43 | -2.48 | -2.37 | -2.29 | -2.31 | -2.46 | -2.35 |
| [Pur]- | -2.56 | -2.60 | -2.45 | -2.49 | -2.51 | -2.52 | -2.41 | -2.38 | -2.51 | -2.43 |
| [BeIm]- | -2.65 | -2.65 | -2.55 | -2.58 | -2.60 | -2.60 | -2.52 | -2.49 | -2.60 | -2.53 |
| [Tyro]- | -2.43 | -2.56 | -2.35 | -2.37 | -2.39 | -2.34 | -2.27 | -2.28 | -2.39 | -2.28 |
| [AspMA]- | -2.17 | -2.48 | -2.11 | -2.14 | -2.21 | -2.09 | -1.99 | -2.01 | -2.22 | -2.09 |
| [H2PO_4_]- | -1.66 | -2.20 | -1.61 | -1.65 | -1.78 | -1.59 | -1.46 | -1.50 | -1.82 | -1.65 |
| [XylSO_3_]- | -1.84 | -1.88 | -1.78 | -1.796 | -1.85 | -1.77 | -1.70 | -1.67 | -1.78 | -1.77 |
| [Gen]- | -1.18 | -1.66 | -1.11 | -1.17 | -1.24 | -1.16 | -1.03 | -1.07 | -1.35 | -1.16 |
| [2NIm]- | -1.52 | -1.70 | -1.46 | -1.49 | -1.58 | -1.53 | -1.41 | -1.38 | -1.61 | -1.54 |
| [EtSO_4_]- | -1.00 | -1.28 | -0.97 | -0.996 | -1.13 | -0.98 | -0.87 | -0.85 | -1.12 | -1.08 |
| [MeSO_4_]- | -0.83 | -1.19 | -0.80 | -0.828 | -0.98 | -0.82 | -0.69 | -0.67 | -1.00 | -0.94 |
| [OMeSO_4_]- | -1.40 | -1.36 | -1.39 | -1.372 | -1.43 | -1.31 | -1.28 | -1.25 | -1.31 | -1.35 |

**Table S3 (continued…):**  COSMO-RS predicted logarithmic activity coefficients (ln(*γ*)) of lignin in different ILs at 363.15 K

| Anion /Cation | [12MeoEMPyrr]+ | [123TMIM]+ | [TMEP]+ | [TB(3OH)PrA]+ | [M12MThEPyr]+ | [VBeTMA]+ | [OMIM]+ | [BeMIM]+ | [DMA-TMA]+ |
| --- | --- | --- | --- | --- | --- | --- | --- | --- | --- |
| [Ace]- | -5.42 | -5.34 | -5.26 | -5.19 | -5.10 | -5.00 | -4.97 | -4.93 | -4.89 |
| [Prop]- | -5.35 | -5.28 | -5.20 | -5.01 | -5.05 | -4.94 | -4.86 | -4.87 | -4.85 |
| [But]- | -5.39 | -5.33 | -5.24 | -4.99 | -5.09 | -4.97 | -4.85 | -4.91 | -4.89 |
| [Valerate]- | -5.42 | -5.38 | -5.28 | -4.96 | -5.14 | -5.01 | -4.85 | -4.94 | -4.93 |
| [N-MCb]- | -5.05 | -4.97 | -4.91 | -4.92 | -4.73 | -4.66 | -4.67 | -4.60 | -4.54 |
| [Hexa]- | -5.47 | -5.44 | -5.34 | -4.95 | -5.20 | -5.05 | -4.86 | -4.99 | -4.99 |
| [Hep]^-^ | -5.51 | -5.49 | -5.38 | -4.94 | -5.25 | -5.09 | -4.85 | -5.03 | -5.03 |
| [Oct]- | -5.55 | -5.54 | -5.42 | -4.92 | -5.30 | -5.12 | -4.86 | -5.07 | -5.08 |
| [Gly]- | -4.83 | -4.75 | -4.69 | -4.74 | -4.53 | -4.48 | -4.52 | -4.41 | -4.34 |
| [Dec]- | -5.58 | -5.59 | -5.46 | -4.87 | -5.35 | -5.15 | -4.83 | -5.10 | -5.12 |
| [Crotonate]- | -4.86 | -4.78 | -4.72 | -4.62 | -4.56 | -4.48 | -4.45 | -4.41 | -4.39 |
| [Pro]- | -4.93 | -4.89 | -4.80 | -4.65 | -4.65 | -4.56 | -4.49 | -4.51 | -4.46 |
| [Ala]- | -4.77 | -4.70 | -4.63 | -4.58 | -4.49 | -4.42 | -4.42 | -4.37 | -4.31 |
| [Lys]- | -4.90 | -4.87 | -4.79 | -4.67 | -4.65 | -4.57 | -4.53 | -4.53 | -4.47 |
| [For]- | -4.37 | -4.28 | -4.23 | -4.40 | -4.05 | -4.03 | -4.14 | -3.97 | -3.89 |
| [Lac]- | -4.40 | -4.32 | -4.24 | -4.37 | -4.11 | -4.06 | -4.11 | -4.00 | -3.92 |
| [2OZ]- | -4.47 | -4.40 | -4.35 | -4.34 | -4.18 | -4.13 | -4.16 | -4.07 | -4.02 |
| [PyZ]- | -4.55 | -4.41 | -4.40 | -4.16 | -4.28 | -4.21 | -4.18 | -4.10 | -4.13 |
| [Glyco]- | -4.13 | -4.02 | -3.96 | -4.21 | -3.82 | -3.80 | -3.90 | -3.74 | -3.63 |
| [EMPO_3_]^-^ | -4.52 | -4.49 | -4.41 | -4.37 | -4.24 | -4.17 | -4.15 | -4.13 | -4.07 |
| [Isole]- | -4.67 | -4.64 | -4.55 | -4.27 | -4.43 | -4.33 | -4.22 | -4.29 | -4.26 |
| [Acryl]- | -4.31 | -4.22 | -4.17 | -4.19 | -4.02 | -3.97 | -4.02 | -3.90 | -3.87 |
| [IsoPMPO_3_]^-^ | -4.52 | -4.50 | -4.41 | -4.25 | -4.26 | -4.19 | -4.12 | -4.15 | -4.10 |
| [Arg]- | -4.13 | -4.03 | -3.99 | -4.20 | -3.91 | -3.89 | -4.01 | -3.84 | -3.74 |
| [Val]- | -4.36 | -4.33 | -4.25 | -4.07 | -4.12 | -4.06 | -4.00 | -4.01 | -3.97 |
| [Abt]- | -4.94 | -4.96 | -4.84 | -4.03 | -4.77 | -4.56 | -4.17 | -4.52 | -4.58 |
| [IMCrb]- | -3.72 | -3.60 | -3.59 | -3.82 | -3.45 | -3.44 | -3.60 | -3.37 | -3.33 |
| [Leu]- | -4.24 | -4.22 | -4.14 | -3.87 | -4.02 | -3.94 | -3.84 | -3.89 | -3.87 |
| [124TAz]- | -3.75 | -3.61 | -3.62 | -3.68 | -3.49 | -3.47 | -3.57 | -3.38 | -3.37 |
| [Met]- | -4.00 | -3.92 | -3.89 | -3.83 | -3.77 | -3.70 | -3.71 | -3.64 | -3.63 |
| [2AIm]- | -3.63 | -3.50 | -3.50 | -3.55 | -3.38 | -3.39 | -3.53 | -3.32 | -3.27 |
| [Ben]- | -3.76 | -3.67 | -3.65 | -3.58 | -3.51 | -3.47 | -3.51 | -3.40 | -3.42 |
| [DOPyrr]^-^ | -3.45 | -3.38 | -3.35 | -3.54 | -3.18 | -3.18 | -3.31 | -3.14 | -3.07 |
| [Glu]- | -3.34 | -3.26 | -3.22 | -3.50 | -3.11 | -3.13 | -3.31 | -3.09 | -2.98 |
| [MCb]^-^ | -3.29 | -3.23 | -3.18 | -3.46 | -3.00 | -3.03 | -3.19 | -2.99 | -2.90 |
| [NMO]- | -3.32 | -3.25 | -3.22 | -3.46 | -3.04 | -3.06 | -3.23 | -3.01 | -2.94 |
| [2MIm]- | -3.64 | -3.54 | -3.53 | -3.30 | -3.42 | -3.41 | -3.44 | -3.33 | -3.33 |
| [Cyst]- | -3.43 | -3.32 | -3.31 | -3.43 | -3.20 | -3.17 | -3.29 | -3.11 | -3.08 |
| [Phe]- | -3.66 | -3.60 | -3.56 | -3.45 | -3.44 | -3.39 | -3.39 | -3.34 | -3.33 |
| [IMZ]- | -3.41 | -3.29 | -3.30 | -3.21 | -3.18 | -3.19 | -3.29 | -3.11 | -3.09 |
| [Thr]- | -3.36 | -3.32 | -3.27 | -3.36 | -3.13 | -3.14 | -3.25 | -3.11 | -3.02 |
| [I3A]- | -3.53 | -3.42 | -3.40 | -3.34 | -3.33 | -3.29 | -3.34 | -3.22 | -3.23 |
| [His]- | -3.17 | -3.05 | -3.03 | -3.26 | -2.96 | -2.97 | -3.13 | -2.91 | -2.84 |
| [3OAcMB]^-^ | -3.40 | -3.35 | -3.31 | -3.32 | -3.15 | -3.14 | -3.21 | -3.10 | -3.06 |
| [Phenolate]- | -3.46 | -3.36 | -3.34 | -3.10 | -3.25 | -3.22 | -3.24 | -3.15 | -3.16 |
| [DEPO_4_]- | -3.32 | -3.31 | -3.25 | -3.31 | -3.07 | -3.06 | -3.12 | -3.05 | -2.97 |
| [Ser]- | -2.93 | -2.84 | -2.80 | -3.07 | -2.70 | -2.73 | -2.90 | -2.69 | -2.56 |
| [2EIm]- | -3.48 | -3.40 | -3.39 | -3.09 | -3.28 | -3.26 | -3.27 | -3.20 | -3.20 |
| [DMO]- | -3.00 | -2.95 | -2.92 | -3.21 | -2.72 | -2.76 | -2.95 | -2.73 | -2.64 |
| [H2PO_3_]- | -2.79 | -2.71 | -2.66 | -2.99 | -2.54 | -2.61 | -2.85 | -2.58 | -2.43 |
| [Aspg]- | -2.70 | -2.60 | -2.57 | -3.02 | -2.48 | -2.55 | -2.82 | -2.50 | -2.37 |
| [DIsoPrPO_4_]- | -3.39 | -3.40 | -3.32 | -3.23 | -3.17 | -3.13 | -3.11 | -3.12 | -3.06 |
| [DMPO_4_]^-^ | -2.96 | -2.92 | -2.87 | -3.11 | -2.69 | -2.73 | -2.89 | -2.71 | -2.61 |
| [DBPO_4_]^-^ | -3.44 | -3.48 | -3.39 | -3.21 | -3.24 | -3.18 | -3.09 | -3.17 | -3.12 |
| [3-(OH)2-MB]^-^ | -2.96 | -2.88 | -2.83 | -2.91 | -2.75 | -2.76 | -2.85 | -2.70 | -2.64 |
| [ClAce]- | -2.83 | -2.77 | -2.73 | -2.95 | -2.58 | -2.64 | -2.81 | -2.60 | -2.51 |
| [ThioSal]- | -3.02 | -2.89 | -2.91 | -2.97 | -2.80 | -2.77 | -2.89 | -2.70 | -2.73 |
| [INDz]- | -3.14 | -3.02 | -3.05 | -2.87 | -2.95 | -2.93 | -2.97 | -2.84 | -2.88 |
| [Try]- | -3.10 | -3.01 | -2.99 | -2.98 | -2.93 | -2.91 | -2.97 | -2.84 | -2.84 |
| [2ClMAcr]- | -3.03 | -2.97 | -2.93 | -2.91 | -2.81 | -2.82 | -2.88 | -2.77 | -2.74 |
| [2BrMAcr]- | -2.91 | -2.78 | -2.80 | -2.85 | -2.71 | -2.65 | -2.75 | -2.57 | -2.63 |
| [2OHMAcr]- | -2.75 | -2.69 | -2.66 | -2.80 | -2.52 | -2.56 | -2.70 | -2.51 | -2.45 |
| [5ATeAz]- | -2.15 | -1.98 | -2.03 | -2.63 | -1.91 | -1.99 | -2.32 | -1.92 | -1.83 |
| [C3C]- | -2.57 | -2.49 | -2.48 | -2.72 | -2.32 | -2.35 | -2.54 | -2.30 | -2.28 |
| [DEABSO_3_]- | -2.83 | -2.87 | -2.80 | -2.80 | -2.63 | -2.62 | -2.63 | -2.62 | -2.54 |
| [Be123TAz]- | -2.57 | -2.44 | -2.49 | -2.61 | -2.37 | -2.38 | -2.51 | -2.30 | -2.32 |
| [IsoPAPSO_3_]- | -2.66 | -2.68 | -2.62 | -2.77 | -2.45 | -2.47 | -2.55 | -2.46 | -2.37 |
| [AceSal]- | -2.64 | -2.58 | -2.56 | -2.64 | -2.42 | -2.44 | -2.57 | -2.39 | -2.38 |
| [DEAPSO_3_]- | -2.73 | -2.76 | -2.69 | -2.76 | -2.52 | -2.53 | -2.58 | -2.53 | -2.44 |
| [56DMBeIm]- | -2.89 | -2.80 | -2.82 | -2.55 | -2.72 | -2.71 | -2.72 | -2.64 | -2.68 |
| [TeAz]- | -2.14 | -1.98 | -2.04 | -2.51 | -1.90 | -1.96 | -2.23 | -1.89 | -1.84 |
| [IsoPABSO_3_]- | -2.53 | -2.54 | -2.49 | -2.70 | -2.31 | -2.34 | -2.46 | -2.34 | -2.24 |
| [DMABSO_3_]- | -2.54 | -2.56 | -2.50 | -2.70 | -2.32 | -2.35 | -2.45 | -2.34 | -2.25 |
| [BAPSO_3_]- | -2.59 | -2.61 | -2.55 | -2.69 | -2.38 | -2.40 | -2.47 | -2.40 | -2.30 |
| [2TFMAcr]- | -2.56 | -2.52 | -2.48 | -2.52 | -2.34 | -2.40 | -2.51 | -2.37 | -2.32 |
| [Tau]- | -1.98 | -1.92 | -1.90 | -2.47 | -1.74 | -1.85 | -2.17 | -1.83 | -1.67 |
| [MeSO_3_]- | -1.97 | -1.92 | -1.89 | -2.46 | -1.70 | -1.81 | -2.12 | -1.79 | -1.65 |
| [HAPSO_3_]- | -2.69 | -2.73 | -2.66 | -2.69 | -2.50 | -2.49 | -2.50 | -2.50 | -2.42 |
| [GluMA]- | -2.19 | -2.11 | -2.07 | -2.28 | -2.00 | -2.03 | -2.17 | -1.99 | -1.89 |
| [Pur]- | -2.31 | -2.17 | -2.22 | -2.39 | -2.11 | -2.14 | -2.31 | -2.07 | -2.08 |
| [BeIm]- | -2.42 | -2.31 | -2.34 | -2.27 | -2.25 | -2.28 | -2.40 | -2.21 | -2.23 |
| [Tyro]- | -2.18 | -2.09 | -2.06 | -2.18 | -2.01 | -2.03 | -2.13 | -1.97 | -1.92 |
| [AspMA]- | -1.88 | -1.78 | -1.76 | -2.09 | -1.69 | -1.75 | -1.95 | -1.70 | -1.59 |
| [H2PO_4_]- | -1.32 | -1.21 | -1.18 | -1.85 | -1.09 | -1.21 | -1.56 | -1.18 | -0.99 |
| [XylSO_3_]- | -1.57 | -1.53 | -1.53 | -1.83 | -1.38 | -1.44 | -1.63 | -1.42 | -1.37 |
| [Gen]- | -0.94 | -0.77 | -0.77 | -1.32 | -0.75 | -0.84 | -1.13 | -0.78 | -0.67 |
| [2NIm]- | -1.28 | -1.19 | -1.22 | -1.56 | -1.11 | -1.22 | -1.50 | -1.17 | -1.13 |
| [EtSO_4_]- | -0.71 | -0.67 | -0.67 | -1.27 | -0.51 | -0.67 | -1.02 | -0.66 | -0.53 |
| [MeSO_4_]- | -0.52 | -0.47 | -0.48 | -1.19 | -0.32 | -0.50 | -0.91 | -0.49 | -0.35 |
| [OMeSO_4_]- | -1.17 | -1.22 | -1.17 | -1.33 | -1.03 | -1.08 | -1.17 | -1.10 | -1.02 |

**Table S3 (continued…):**  COSMO-RS predicted logarithmic activity coefficients (ln(*γ*)) of lignin in different ILs at 363.15 K

| Anion /Cation | [BeEtoMIM]+ | [HMIM]+ | [TMGH]+ | [BPy]+ | [BMIM]+ | [TEM]+ | [BeTMA]+ | [2ETeG]+ | [p-AnisEt2NH]+ | [EMIM]+ | [EPy]+ | [AMIM]+ |
| --- | --- | --- | --- | --- | --- | --- | --- | --- | --- | --- | --- | --- |
| [Ace]- | -4.83 | -4.82 | -4.83 | -4.68 | -4.61 | -4.60 | -4.55 | -4.47 | -4.42 | -4.28 | -4.27 | -4.25 |
| [Prop]- | -4.76 | -4.74 | -4.78 | -4.63 | -4.55 | -4.51 | -4.53 | -4.34 | -4.32 | -4.25 | -4.26 | -4.22 |
| [But]- | -4.78 | -4.75 | -4.84 | -4.67 | -4.58 | -4.56 | -4.59 | -4.36 | -4.35 | -4.31 | -4.33 | -4.28 |
| [Valerate]- | -4.80 | -4.76 | -4.90 | -4.70 | -4.61 | -4.59 | -4.64 | -4.38 | -4.38 | -4.36 | -4.39 | -4.34 |
| [N-MCb]- | -4.51 | -4.53 | -4.49 | -4.36 | -4.32 | -4.30 | -4.22 | -4.20 | -4.13 | -3.97 | -3.95 | -3.94 |
| [Hexa]- | -4.83 | -4.78 | -4.97 | -4.75 | -4.65 | -4.64 | -4.71 | -4.41 | -4.42 | -4.43 | -4.47 | -4.40 |
| [Hep]^-^ | -4.85 | -4.79 | -5.03 | -4.78 | -4.68 | -4.68 | -4.76 | -4.43 | -4.44 | -4.49 | -4.53 | -4.46 |
| [Oct]- | -4.88 | -4.81 | -5.08 | -4.82 | -4.71 | -4.72 | -4.81 | -4.45 | -4.47 | -4.54 | -4.59 | -4.51 |
| [Gly]- | -4.35 | -4.37 | -4.28 | -4.19 | -4.14 | -4.11 | -4.03 | -4.02 | -3.97 | -3.79 | -3.76 | -3.77 |
| [Dec]- | -4.89 | -4.80 | -5.15 | -4.85 | -4.73 | -4.75 | -4.87 | -4.46 | -4.49 | -4.60 | -4.67 | -4.57 |
| [Crotonate]- | -4.32 | -4.33 | -4.31 | -4.20 | -4.14 | -4.10 | -4.08 | -3.96 | -3.92 | -3.84 | -3.83 | -3.81 |
| [Pro]- | -4.40 | -4.39 | -4.44 | -4.28 | -4.22 | -4.20 | -4.17 | -4.05 | -4.02 | -3.95 | -3.95 | -3.92 |
| [Ala]- | -4.29 | -4.29 | -4.25 | -4.14 | -4.09 | -4.04 | -4.01 | -3.92 | -3.90 | -3.77 | -3.76 | -3.75 |
| [Lys]- | -4.43 | -4.42 | -4.46 | -4.31 | -4.26 | -4.23 | -4.20 | -4.11 | -4.07 | -3.98 | -3.98 | -3.96 |
| [For]- | -3.93 | -3.97 | -3.81 | -3.76 | -3.73 | -3.69 | -3.57 | -3.64 | -3.56 | -3.35 | -3.31 | -3.32 |
| [Lac]- | -3.96 | -3.95 | -3.85 | -3.76 | -3.72 | -3.68 | -3.61 | -3.62 | -3.58 | -3.37 | -3.34 | -3.35 |
| [2OZ]- | -4.00 | -4.03 | -3.95 | -3.87 | -3.83 | -3.78 | -3.72 | -3.66 | -3.63 | -3.50 | -3.48 | -3.47 |
| [PyZ]- | -4.06 | -4.04 | -3.94 | -3.91 | -3.84 | -3.64 | -3.83 | -3.40 | -3.52 | -3.50 | -3.51 | -3.47 |
| [Glyco]- | -3.72 | -3.72 | -3.55 | -3.50 | -3.47 | -3.42 | -3.33 | -3.39 | -3.35 | -3.07 | -3.03 | -3.06 |
| [EMPO_3_]^-^ | -4.03 | -4.05 | -4.07 | -3.93 | -3.89 | -3.89 | -3.79 | -3.79 | -3.71 | -3.61 | -3.59 | -3.58 |
| [Isole]- | -4.17 | -4.14 | -4.22 | -4.08 | -4.00 | -3.93 | -4.00 | -3.74 | -3.76 | -3.76 | -3.79 | -3.74 |
| [Acryl]- | -3.85 | -3.88 | -3.76 | -3.72 | -3.67 | -3.59 | -3.57 | -3.49 | -3.46 | -3.33 | -3.32 | -3.31 |
| [IsoPMPO_3_]^-^ | -4.04 | -4.03 | -4.08 | -3.95 | -3.88 | -3.85 | -3.84 | -3.72 | -3.68 | -3.64 | -3.65 | -3.61 |
| [Arg]- | -3.83 | -3.84 | -3.67 | -3.62 | -3.60 | -3.52 | -3.48 | -3.49 | -3.48 | -3.24 | -3.20 | -3.24 |
| [Val]- | -3.92 | -3.90 | -3.91 | -3.81 | -3.74 | -3.66 | -3.71 | -3.51 | -3.52 | -3.49 | -3.50 | -3.47 |
| [Abt]- | -4.30 | -4.18 | -4.56 | -4.30 | -4.16 | -4.08 | -4.37 | -3.71 | -3.84 | -4.10 | -4.20 | -4.06 |
| [IMCrb]- | -3.36 | -3.42 | -3.20 | -3.20 | -3.18 | -3.10 | -3.03 | -3.06 | -3.02 | -2.81 | -2.76 | -2.79 |
| [Leu]- | -3.79 | -3.76 | -3.82 | -3.70 | -3.63 | -3.54 | -3.63 | -3.36 | -3.38 | -3.40 | -3.43 | -3.38 |
| [124TAz]- | -3.38 | -3.41 | -3.21 | -3.21 | -3.18 | -3.05 | -3.09 | -2.92 | -2.97 | -2.81 | -2.78 | -2.79 |
| [Met]- | -3.58 | -3.60 | -3.56 | -3.48 | -3.44 | -3.36 | -3.36 | -3.23 | -3.22 | -3.16 | -3.15 | -3.13 |
| [2AIm]- | -3.33 | -3.36 | -3.12 | -3.15 | -3.13 | -2.96 | -3.00 | -2.84 | -2.91 | -2.75 | -2.71 | -2.73 |
| [Ben]- | -3.35 | -3.39 | -3.28 | -3.27 | -3.21 | -3.10 | -3.15 | -2.96 | -2.97 | -2.92 | -2.93 | -2.90 |
| [DOPyrr]^-^ | -3.11 | -3.18 | -3.00 | -2.98 | -2.97 | -2.92 | -2.79 | -2.88 | -2.80 | -2.64 | -2.59 | -2.61 |
| [Glu]- | -3.10 | -3.14 | -2.89 | -2.92 | -2.90 | -2.79 | -2.73 | -2.80 | -2.76 | -2.54 | -2.49 | -2.53 |
| [MCb]^-^ | -2.97 | -3.05 | -2.83 | -2.84 | -2.83 | -2.78 | -2.62 | -2.78 | -2.68 | -2.48 | -2.42 | -2.46 |
| [NMO]- | -3.00 | -3.08 | -2.87 | -2.88 | -2.87 | -2.81 | -2.66 | -2.80 | -2.70 | -2.53 | -2.48 | -2.50 |
| [2MIm]- | -3.31 | -3.32 | -3.16 | -3.19 | -3.14 | -2.93 | -3.09 | -2.71 | -2.84 | -2.83 | -2.83 | -2.81 |
| [Cyst]- | -3.10 | -3.15 | -2.97 | -2.97 | -2.94 | -2.84 | -2.81 | -2.77 | -2.75 | -2.61 | -2.57 | -2.59 |
| [Phe]- | -3.27 | -3.29 | -3.24 | -3.20 | -3.15 | -3.05 | -3.10 | -2.91 | -2.92 | -2.90 | -2.90 | -2.88 |
| [IMZ]- | -3.11 | -3.14 | -2.90 | -2.97 | -2.93 | -2.71 | -2.84 | -2.53 | -2.65 | -2.58 | -2.57 | -2.56 |
| [Thr]- | -3.08 | -3.12 | -2.95 | -2.96 | -2.93 | -2.83 | -2.77 | -2.79 | -2.75 | -2.62 | -2.59 | -2.60 |
| [I3A]- | -3.20 | -3.21 | -3.05 | -3.08 | -3.02 | -2.84 | -3.00 | -2.70 | -2.77 | -2.73 | -2.74 | -2.71 |
| [His]- | -2.94 | -2.96 | -2.70 | -2.74 | -2.71 | -2.56 | -2.60 | -2.53 | -2.56 | -2.35 | -2.32 | -2.35 |
| [3OAcMB]^-^ | -3.05 | -3.10 | -2.98 | -2.98 | -2.94 | -2.85 | -2.81 | -2.75 | -2.72 | -2.67 | -2.65 | -2.64 |
| [Phenolate]- | -3.12 | -3.13 | -2.95 | -3.03 | -2.95 | -2.69 | -2.93 | -2.47 | -2.61 | -2.67 | -2.70 | -2.64 |
| [DEPO_4_]- | -2.98 | -3.03 | -2.96 | -2.91 | -2.88 | -2.86 | -2.73 | -2.81 | -2.72 | -2.63 | -2.60 | -2.61 |
| [Ser]- | -2.72 | -2.73 | -2.46 | -2.51 | -2.49 | -2.35 | -2.32 | -2.35 | -2.35 | -2.12 | -2.08 | -2.12 |
| [2EIm]- | -3.16 | -3.17 | -3.06 | -3.06 | -3.01 | -2.81 | -2.98 | -2.58 | -2.72 | -2.74 | -2.75 | -2.72 |
| [DMO]- | -2.71 | -2.81 | -2.59 | -2.61 | -2.62 | -2.57 | -2.37 | -2.59 | -2.46 | -2.28 | -2.22 | -2.25 |
| [H2PO_3_]- | -2.63 | -2.67 | -2.30 | -2.43 | -2.41 | -2.22 | -2.18 | -2.25 | -2.24 | -2.00 | -1.96 | -2.00 |
| [Aspg]- | -2.56 | -2.62 | -2.25 | -2.35 | -2.35 | -2.19 | -2.12 | -2.26 | -2.22 | -1.94 | -1.87 | -1.94 |
| [DIsoPrPO_4_]- | -3.02 | -3.04 | -3.07 | -2.97 | -2.93 | -2.91 | -2.84 | -2.82 | -2.76 | -2.73 | -2.72 | -2.71 |
| [DMPO_4_]^-^ | -2.69 | -2.76 | -2.56 | -2.58 | -2.57 | -2.51 | -2.36 | -2.52 | -2.42 | -2.26 | -2.21 | -2.24 |
| [DBPO_4_]^-^ | -3.05 | -3.06 | -3.16 | -3.03 | -2.98 | -2.98 | -2.92 | -2.86 | -2.80 | -2.83 | -2.83 | -2.80 |
| [3-(OH)2-MB]^-^ | -2.70 | -2.71 | -2.50 | -2.55 | -2.51 | -2.33 | -2.42 | -2.25 | -2.31 | -2.19 | -2.19 | -2.19 |
| [ClAce]- | -2.60 | -2.67 | -2.39 | -2.48 | -2.45 | -2.32 | -2.27 | -2.30 | -2.26 | -2.11 | -2.08 | -2.10 |
| [ThioSal]- | -2.69 | -2.76 | -2.56 | -2.60 | -2.57 | -2.43 | -2.48 | -2.32 | -2.34 | -2.27 | -2.25 | -2.24 |
| [INDz]- | -2.83 | -2.86 | -2.68 | -2.74 | -2.69 | -2.47 | -2.66 | -2.26 | -2.39 | -2.41 | -2.41 | -2.38 |
| [Try]- | -2.83 | -2.85 | -2.69 | -2.72 | -2.67 | -2.50 | -2.63 | -2.39 | -2.45 | -2.40 | -2.40 | -2.39 |
| [2ClMAcr]- | -2.74 | -2.78 | -2.61 | -2.66 | -2.61 | -2.45 | -2.53 | -2.34 | -2.37 | -2.34 | -2.34 | -2.32 |
| [2BrMAcr]- | -2.56 | -2.63 | -2.47 | -2.48 | -2.45 | -2.34 | -2.38 | -2.23 | -2.24 | -2.16 | -2.14 | -2.14 |
| [2OHMAcr]- | -2.51 | -2.58 | -2.33 | -2.41 | -2.39 | -2.25 | -2.22 | -2.21 | -2.19 | -2.07 | -2.04 | -2.06 |
| [5ATeAz]- | -2.02 | -2.11 | -1.68 | -1.77 | -1.82 | -1.70 | -1.57 | -1.78 | -1.73 | -1.37 | -1.26 | -1.36 |
| [C3C]- | -2.31 | -2.41 | -2.17 | -2.23 | -2.23 | -2.15 | -2.04 | -2.13 | -2.05 | -1.93 | -1.89 | -1.90 |
| [DEABSO_3_]- | -2.53 | -2.58 | -2.589 | -2.50 | -2.49 | -2.50 | -2.35 | -2.467 | -2.35 | -2.31 | -2.28 | -2.28 |
| [Be123TAz]- | -2.32 | -2.39 | -2.16 | -2.20 | -2.20 | -2.07 | -2.09 | -1.98 | -2.00 | -1.89 | -1.84 | -1.86 |
| [IsoPAPSO_3_]- | -2.41 | -2.47 | -2.396 | -2.35 | -2.35 | -2.35 | -2.16 | -2.358 | -2.23 | -2.12 | -2.07 | -2.10 |
| [AceSal]- | -2.38 | -2.46 | -2.25 | -2.32 | -2.30 | -2.17 | -2.15 | -2.09 | -2.08 | -2.02 | -2.00 | -2.00 |
| [DEAPSO_3_]- | -2.46 | -2.51 | -2.475 | -2.41 | -2.41 | -2.41 | -2.24 | -2.390 | -2.27 | -2.20 | -2.16 | -2.17 |
| [56DMBeIm]- | -2.62 | -2.64 | -2.52 | -2.55 | -2.51 | -2.31 | -2.48 | -2.10 | -2.22 | -2.28 | -2.28 | -2.25 |
| [TeAz]- | -1.96 | -2.06 | -1.69 | -1.77 | -1.81 | -1.71 | -1.58 | -1.74 | -1.69 | -1.41 | -1.31 | -1.39 |
| [IsoPABSO_3_]- | -2.30 | -2.37 | -2.251 | -2.23 | -2.24 | -2.22 | -2.03 | -2.248 | -2.12 | -1.99 | -1.93 | -1.96 |
| [DMABSO_3_]- | -2.29 | -2.37 | -2.270 | -2.24 | -2.24 | -2.24 | -2.04 | -2.258 | -2.12 | -2.01 | -1.95 | -1.98 |
| [BAPSO_3_]- | -2.34 | -2.40 | -2.335 | -2.28 | -2.28 | -2.28 | -2.10 | -2.289 | -2.16 | -2.07 | -2.02 | -2.04 |
| [2TFMAcr]- | -2.35 | -2.40 | -2.19 | -2.28 | -2.24 | -2.07 | -2.13 | -2.00 | -2.01 | -1.97 | -1.96 | -1.96 |
| [Tau]- | -1.89 | -2.00 | -1.602 | -1.72 | -1.75 | -1.66 | -1.44 | -1.795 | -1.66 | -1.36 | -1.27 | -1.35 |
| [MeSO_3_]- | -1.83 | -1.96 | -1.578 | -1.69 | -1.73 | -1.65 | -1.40 | -1.778 | -1.62 | -1.34 | -1.25 | -1.32 |
| [HAPSO_3_]- | -2.41 | -2.46 | -2.472 | -2.38 | -2.37 | -2.39 | -2.23 | -2.358 | -2.24 | -2.20 | -2.17 | -2.17 |
| [GluMA]- | -2.02 | -2.02 | -1.76 | -1.84 | -1.81 | -1.64 | -1.68 | -1.62 | -1.66 | -1.49 | -1.47 | -1.50 |
| [Pur]- | -2.11 | -2.18 | -1.92 | -1.98 | -1.99 | -1.86 | -1.86 | -1.80 | -1.82 | -1.67 | -1.61 | -1.65 |
| [BeIm]- | -2.24 | -2.29 | -2.03 | -2.14 | -2.12 | -1.90 | -2.03 | -1.75 | -1.86 | -1.84 | -1.82 | -1.82 |
| [Tyro]- | -1.99 | -1.99 | -1.77 | -1.84 | -1.81 | -1.63 | -1.74 | -1.56 | -1.64 | -1.52 | -1.51 | -1.52 |
| [AspMA]- | -1.77 | -1.79 | -1.44 | -1.57 | -1.56 | -1.37 | -1.39 | -1.39 | -1.42 | -1.20 | -1.17 | -1.21 |
| [H2PO_4_]- | -1.30 | -1.34 | -0.85 | -1.04 | -1.05 | -0.88 | -0.77 | -1.03 | -0.99 | -0.61 | -0.53 | -0.63 |
| [XylSO_3_]- | -1.43 | -1.54 | -1.297 | -1.38 | -1.39 | -1.33 | -1.19 | -1.365 | -1.26 | -1.14 | -1.09 | -1.12 |
| [Gen]- | -0.91 | -0.92 | -0.45 | -0.64 | -0.64 | -0.42 | -0.48 | -0.48 | -0.56 | -0.24 | -0.19 | -0.25 |
| [2NIm]- | -1.25 | -1.37 | -0.95 | -1.15 | -1.17 | -0.99 | -0.95 | -1.03 | -1.01 | -0.84 | -0.78 | -0.83 |
| [EtSO_4_]- | -0.73 | -0.88 | -0.427 | -0.65 | -0.68 | -0.56 | -0.35 | -0.730 | -0.58 | -0.35 | -0.27 | -0.34 |
| [MeSO_4_]- | -0.59 | -0.75 | -0.213 | -0.48 | -0.53 | -0.38 | -0.16 | -0.583 | -0.43 | -0.15 | -0.06 | -0.15 |
| [OMeSO_4_]- | -1.06 | -1.14 | -1.026 | -1.06 | -1.07 | -1.03 | -0.90 | -1.064 | -0.95 | -0.92 | -0.88 | -0.90 |

**Table S3 (continued…):**  COSMO-RS predicted logarithmic activity coefficients (ln(*γ*)) of lignin in different ILs at 363.15 K

| Anion /Cation | [TMA]+ | [13DMIM]+ | [VanEt2NH]+ | [FurEt2NH]+ | [DEMA]+ | [DMOA]+ | [(3OH)PrTMA]+ | [TMEOHP]+ | [BeDM(2OH)EA]+ | [ACh]+ |
| --- | --- | --- | --- | --- | --- | --- | --- | --- | --- | --- |
| [Ace]- | -4.08 | -3.88 | -3.66 | -3.62 | -3.50 | -3.14 | -2.94 | -2.84 | -2.76 | -2.76 |
| [Prop]- | -4.11 | -3.88 | -3.59 | -3.57 | -3.45 | -3.05 | -2.96 | -2.84 | -2.76 | -2.86 |
| [But]- | -4.21 | -3.97 | -3.62 | -3.62 | -3.53 | -3.08 | -3.07 | -2.95 | -2.84 | -2.99 |
| [Valerate]- | -4.30 | -4.05 | -3.66 | -3.67 | -3.60 | -3.09 | -3.17 | -3.04 | -2.92 | -3.12 |
| [N-MCb]- | -3.73 | -3.57 | -3.42 | -3.36 | -3.23 | -2.93 | -2.66 | -2.57 | -2.52 | -2.44 |
| [Hexa]- | -4.41 | -4.14 | -3.70 | -3.73 | -3.67 | -3.13 | -3.28 | -3.14 | -3.00 | -3.26 |
| [Hep]^-^ | -4.49 | -4.21 | -3.73 | -3.77 | -3.73 | -3.14 | -3.37 | -3.22 | -3.07 | -3.38 |
| [Oct]- | -4.58 | -4.28 | -3.76 | -3.81 | -3.79 | -3.16 | -3.45 | -3.30 | -3.14 | -3.49 |
| [Gly]- | -3.51 | -3.38 | -3.27 | -3.21 | -3.06 | -2.81 | -2.46 | -2.38 | -2.35 | -2.24 |
| [Dec]- | -4.70 | -4.38 | -3.79 | -3.86 | -3.87 | -3.17 | -3.58 | -3.42 | -3.23 | -3.67 |
| [Crotonate]- | -3.66 | -3.48 | -3.24 | -3.21 | -3.10 | -2.76 | -2.61 | -2.51 | -2.44 | -2.46 |
| [Pro]- | -3.78 | -3.61 | -3.34 | -3.33 | -3.23 | -2.84 | -2.73 | -2.63 | -2.55 | -2.60 |
| [Ala]- | -3.55 | -3.40 | -3.21 | -3.18 | -3.04 | -2.74 | -2.50 | -2.41 | -2.37 | -2.34 |
| [Lys]- | -3.79 | -3.65 | -3.41 | -3.41 | -3.31 | -2.96 | -2.78 | -2.70 | -2.63 | -2.64 |
| [For]- | -3.01 | -2.93 | -2.89 | -2.80 | -2.63 | -2.46 | -2.03 | -1.96 | -1.96 | -1.73 |
| [Lac]- | -3.09 | -2.97 | -2.91 | -2.82 | -2.65 | -2.43 | -2.10 | -2.02 | -2.01 | -1.89 |
| [2OZ]- | -3.25 | -3.12 | -2.97 | -2.92 | -2.78 | -2.52 | -2.23 | -2.15 | -2.11 | -2.05 |
| [PyZ]- | -3.36 | -3.13 | -2.81 | -2.81 | -2.61 | -2.32 | -2.19 | -2.06 | -2.05 | -2.22 |
| [Glyco]- | -2.74 | -2.65 | -2.69 | -2.55 | -2.35 | -2.22 | -1.78 | -1.72 | -1.74 | -1.52 |
| [EMPO_3_]^-^ | -3.39 | -3.27 | -3.09 | -3.05 | -2.96 | -2.63 | -2.46 | -2.38 | -2.31 | -2.23 |
| [Isole]- | -3.67 | -3.47 | -3.11 | -3.14 | -3.04 | -2.62 | -2.63 | -2.52 | -2.44 | -2.59 |
| [Acryl]- | -3.10 | -2.96 | -2.81 | -2.76 | -2.61 | -2.39 | -2.11 | -2.03 | -2.01 | -1.92 |
| [IsoPMPO_3_]^-^ | -3.48 | -3.33 | -3.06 | -3.06 | -2.97 | -2.59 | -2.51 | -2.42 | -2.35 | -2.38 |
| [Arg]- | -2.94 | -2.87 | -2.86 | -2.80 | -2.61 | -2.52 | -2.05 | -1.99 | -2.03 | -1.83 |
| [Val]- | -3.33 | -3.18 | -2.90 | -2.90 | -2.78 | -2.44 | -2.34 | -2.24 | -2.20 | -2.26 |
| [Abt]- | -4.30 | -3.95 | -3.21 | -3.33 | -3.33 | -2.59 | -3.23 | -3.04 | -2.85 | -3.51 |
| [IMCrb]- | -2.50 | -2.42 | -2.43 | -2.34 | -2.16 | -2.10 | -1.63 | -1.57 | -1.60 | -1.36 |
| [Leu]- | -3.30 | -3.12 | -2.78 | -2.80 | -2.70 | -2.31 | -2.32 | -2.21 | -2.16 | -2.28 |
| [124TAz]- | -2.56 | -2.42 | -2.35 | -2.30 | -2.09 | -1.98 | -1.58 | -1.51 | -1.54 | -1.43 |
| [Met]- | -2.98 | -2.84 | -2.65 | -2.63 | -2.52 | -2.26 | -2.08 | -2.00 | -1.96 | -1.93 |
| [2AIm]- | -2.44 | -2.36 | -2.28 | -2.25 | -2.02 | -1.94 | -1.43 | -1.36 | -1.41 | -1.34 |
| [Ben]- | -2.76 | -2.61 | -2.41 | -2.38 | -2.25 | -2.04 | -1.86 | -1.76 | -1.75 | -1.73 |
| [DOPyrr]^-^ | -2.30 | -2.27 | -2.25 | -2.16 | -2.02 | -1.90 | -1.47 | -1.43 | -1.43 | -1.17 |
| [Glu]- | -2.17 | -2.16 | -2.21 | -2.11 | -1.91 | -1.89 | -1.36 | -1.31 | -1.38 | -1.09 |
| [MCb]^-^ | -2.09 | -2.09 | -2.14 | -2.03 | -1.88 | -1.80 | -1.31 | -1.28 | -1.31 | -0.95 |
| [NMO]- | -2.17 | -2.15 | -2.16 | -2.07 | -1.93 | -1.85 | -1.38 | -1.33 | -1.35 | -1.05 |
| [2MIm]- | -2.65 | -2.50 | -2.25 | -2.26 | -2.06 | -1.86 | -1.59 | -1.48 | -1.51 | -1.66 |
| [Cyst]- | -2.34 | -2.26 | -2.21 | -2.16 | -1.99 | -1.90 | -1.50 | -1.44 | -1.46 | -1.29 |
| [Phe]- | -2.75 | -2.62 | -2.39 | -2.39 | -2.28 | -2.03 | -1.90 | -1.82 | -1.79 | -1.79 |
| [IMZ]- | -2.34 | -2.22 | -2.06 | -2.04 | -1.81 | -1.70 | -1.32 | -1.23 | -1.28 | -1.31 |
| [Thr]- | -2.30 | -2.28 | -2.21 | -2.17 | -2.00 | -1.87 | -1.47 | -1.41 | -1.44 | -1.25 |
| [I3A]- | -2.60 | -2.43 | -2.23 | -2.21 | -2.04 | -1.89 | -1.70 | -1.60 | -1.63 | -1.67 |
| [His]- | -2.06 | -1.99 | -2.01 | -1.93 | -1.71 | -1.71 | -1.24 | -1.18 | -1.26 | -1.06 |
| [3OAcMB]^-^ | -2.42 | -2.36 | -2.21 | -2.17 | -2.05 | -1.85 | -1.61 | -1.54 | -1.52 | -1.42 |
| [Phenolate]- | -2.53 | -2.36 | -2.05 | -2.06 | -1.86 | -1.66 | -1.53 | -1.42 | -1.45 | -1.58 |
| [DEPO_4_]- | -2.35 | -2.33 | -2.23 | -2.17 | -2.09 | -1.87 | -1.62 | -1.57 | -1.54 | -1.34 |
| [Ser]- | -1.74 | -1.73 | -1.81 | -1.70 | -1.46 | -1.45 | -0.92 | -0.87 | -0.97 | -0.69 |
| [2EIm]- | -2.59 | -2.45 | -2.16 | -2.19 | -2.01 | -1.79 | -1.58 | -1.46 | -1.47 | -1.67 |
| [DMO]- | -1.88 | -1.92 | -1.97 | -1.86 | -1.73 | -1.68 | -1.17 | -1.15 | -1.16 | -0.79 |
| [H2PO_3_]- | -1.55 | -1.60 | -1.72 | -1.59 | -1.31 | -1.37 | -0.73 | -0.69 | -0.82 | -0.46 |
| [Aspg]- | -1.49 | -1.53 | -1.70 | -1.58 | -1.32 | -1.45 | -0.74 | -0.71 | -0.84 | -0.42 |
| [DIsoPrPO_4_]- | -2.53 | -2.47 | -2.27 | -2.25 | -2.19 | -1.90 | -1.79 | -1.72 | -1.67 | -1.60 |
| [DMPO_4_]^-^ | -1.89 | -1.91 | -1.93 | -1.85 | -1.71 | -1.63 | -1.18 | -1.15 | -1.17 | -0.84 |
| [DBPO_4_]^-^ | -2.68 | -2.61 | -2.33 | -2.33 | -2.31 | -1.94 | -1.96 | -1.89 | -1.80 | -1.81 |
| [3-(OH)2-MB]^-^ | -1.94 | -1.87 | -1.79 | -1.73 | -1.52 | -1.43 | -1.11 | -1.04 | -1.10 | -0.99 |
| [ClAce]- | -1.76 | -1.76 | -1.77 | -1.69 | -1.50 | -1.47 | -1.00 | -0.96 | -1.04 | -0.72 |
| [ThioSal]- | -2.08 | -1.96 | -1.86 | -1.82 | -1.67 | -1.60 | -1.31 | -1.24 | -1.25 | -1.14 |
| [INDz]- | -2.27 | -2.11 | -1.87 | -1.88 | -1.70 | -1.55 | -1.36 | -1.26 | -1.29 | -1.37 |
| [Try]- | -2.26 | -2.13 | -1.97 | -1.95 | -1.79 | -1.68 | -1.46 | -1.38 | -1.42 | -1.39 |
| [2ClMAcr]- | -2.13 | -2.04 | -1.88 | -1.86 | -1.70 | -1.56 | -1.32 | -1.25 | -1.29 | -1.19 |
| [2BrMAcr]- | -2.00 | -1.87 | -1.77 | -1.73 | -1.61 | -1.52 | -1.25 | -1.18 | -1.19 | -1.08 |
| [2OHMAcr]- | -1.75 | -1.74 | -1.72 | -1.66 | -1.48 | -1.43 | -1.01 | -0.96 | -1.02 | -0.77 |
| [5ATeAz]- | -0.88 | -0.93 | -1.24 | -1.09 | -0.83 | -1.08 | -0.23 | -0.23 | -0.39 | 0.20 |
| [C3C]- | -1.63 | -1.62 | -1.64 | -1.56 | -1.43 | -1.42 | -1.00 | -0.96 | -0.98 | -0.69 |
| [DEABSO_3_]- | -2.05 | -2.07 | -1.94 | -1.91 | -1.87 | -1.63 | -1.44 | -1.40 | -1.35 | -1.18 |
| [Be123TAz]- | -1.65 | -1.57 | -1.55 | -1.50 | -1.34 | -1.34 | -0.94 | -0.90 | -0.95 | -0.73 |
| [IsoPAPSO_3_]- | -1.80 | -1.85 | -1.82 | -1.76 | -1.69 | -1.54 | -1.22 | -1.20 | -1.19 | -0.88 |
| [AceSal]- | -1.77 | -1.73 | -1.65 | -1.60 | -1.47 | -1.39 | -1.08 | -1.02 | -1.04 | -0.88 |
| [DEAPSO_3_]- | -1.92 | -1.94 | -1.86 | -1.81 | -1.76 | -1.56 | -1.30 | -1.27 | -1.24 | -1.02 |
| [56DMBeIm]- | -2.16 | -2.03 | -1.76 | -1.80 | -1.65 | -1.47 | -1.31 | -1.21 | -1.22 | -1.37 |
| [TeAz]- | -0.98 | -1.01 | -1.23 | -1.10 | -0.89 | -1.06 | -0.34 | -0.34 | -0.46 | 0.06 |
| [IsoPABSO_3_]- | -1.63 | -1.70 | -1.71 | -1.64 | -1.55 | -1.45 | -1.06 | -1.04 | -1.05 | -0.69 |
| [DMABSO_3_]- | -1.66 | -1.72 | -1.72 | -1.64 | -1.57 | -1.44 | -1.10 | -1.09 | -1.08 | -0.73 |
| [BAPSO_3_]- | -1.75 | -1.80 | -1.76 | -1.71 | -1.64 | -1.49 | -1.19 | -1.17 | -1.15 | -0.85 |
| [2TFMAcr]- | -1.72 | -1.69 | -1.57 | -1.54 | -1.39 | -1.32 | -1.00 | -0.94 | -1.00 | -0.83 |
| [Tau]- | -0.82 | -0.97 | -1.24 | -1.09 | -0.87 | -1.05 | -0.25 | -0.26 | -0.39 | 0.22 |
| [MeSO_3_]- | -0.81 | -0.95 | -1.21 | -1.04 | -0.85 | -0.99 | -0.25 | -0.27 | -0.38 | 0.26 |
| [HAPSO_3_]- | -1.95 | -1.97 | -1.85 | -1.82 | -1.79 | -1.55 | -1.39 | -1.36 | -1.31 | -1.10 |
| [GluMA]- | -1.18 | -1.17 | -1.21 | -1.11 | -0.88 | -0.86 | -0.46 | -0.41 | -0.51 | -0.30 |
| [Pur]- | -1.41 | -1.36 | -1.39 | -1.36 | -1.19 | -1.25 | -0.74 | -0.70 | -0.78 | -0.52 |
| [BeIm]- | -1.63 | -1.56 | -1.43 | -1.44 | -1.24 | -1.22 | -0.84 | -0.77 | -0.85 | -0.81 |
| [Tyro]- | -1.30 | -1.23 | -1.20 | -1.13 | -0.93 | -0.90 | -0.60 | -0.53 | -0.62 | -0.50 |
| [AspMA]- | -0.83 | -0.86 | -0.99 | -0.86 | -0.61 | -0.69 | -0.16 | -0.12 | -0.26 | 0.06 |
| [H2PO_4_]- | -0.05 | -0.19 | -0.57 | -0.35 | -0.03 | -0.29 | 0.53 | 0.53 | 0.31 | 0.93 |
| [XylSO_3_]- | -0.81 | -0.88 | -0.96 | -0.88 | -0.78 | -0.84 | -0.38 | -0.38 | -0.44 | -0.01 |
| [Gen]- | 0.15 | 0.13 | -0.16 | 0.03 | 0.34 | 0.09 | 0.73 | 0.77 | 0.56 | 0.95 |
| [2NIm]- | -0.46 | -0.54 | -0.71 | -0.63 | -0.42 | -0.65 | 0.04 | 0.04 | -0.11 | 0.34 |
| [EtSO_4_]- | 0.17 | -0.03 | -0.34 | -0.20 | 0.00 | -0.29 | 0.49 | 0.44 | 0.27 | 1.02 |
| [MeSO_4_]- | 0.43 | 0.20 | -0.20 | -0.03 | 0.20 | -0.17 | 0.73 | 0.68 | 0.46 | 1.30 |
| [OMeSO_4_]- | -0.63 | -0.73 | -0.74 | -0.68 | -0.63 | -0.59 | -0.32 | -0.33 | -0.38 | 0.03 |

**Table S3 (continued…):**  COSMO-RS predicted logarithmic activity coefficients (ln(*γ*)) of lignin in different ILs at 363.15 K

| Anion /Cation | [DMBA]+ | [MPyrr]+ | [EUrMIM]+ | [12HyE3MIM]+ | [Ch]+ | [CPOPMIM]+ | [D(OH)EDMA]+ | [Tris(2OH)EMA]+ | [DMUr]+ | [Gun]+ |
| --- | --- | --- | --- | --- | --- | --- | --- | --- | --- | --- |
| [Ace]- | -2.70 | -2.67 | -2.43 | -2.16 | -2.21 | -2.09 | -0.62 | 0.73 | 0.98 | 4.37 |
| [Prop]- | -2.66 | -2.65 | -2.49 | -2.20 | -2.26 | -2.09 | -0.71 | 0.60 | 0.76 | 3.98 |
| [But]- | -2.74 | -2.75 | -2.60 | -2.31 | -2.39 | -2.18 | -0.86 | 0.43 | 0.56 | 3.73 |
| [Valerate]- | -2.80 | -2.83 | -2.70 | -2.41 | -2.51 | -2.27 | -1.00 | 0.28 | 0.38 | 3.50 |
| [N-MCb]- | -2.48 | -2.44 | -2.20 | -1.94 | -1.94 | -1.88 | -0.43 | 0.87 | 1.12 | 4.37 |
| [Hexa]- | -2.87 | -2.93 | -2.81 | -2.51 | -2.63 | -2.36 | -1.14 | 0.12 | 0.19 | 3.28 |
| [Hep]^-^ | -2.93 | -3.00 | -2.90 | -2.60 | -2.74 | -2.43 | -1.27 | -0.01 | 0.04 | 3.09 |
| [Oct]- | -2.98 | -3.07 | -2.99 | -2.69 | -2.84 | -2.51 | -1.38 | -0.14 | -0.11 | 2.91 |
| [Gly]- | -2.34 | -2.28 | -2.07 | -1.77 | -1.75 | -1.71 | -0.26 | 1.01 | 1.20 | 4.32 |
| [Dec]- | -3.05 | -3.17 | -3.12 | -2.82 | -3.00 | -2.62 | -1.57 | -0.35 | -0.35 | 2.61 |
| [Crotonate]- | -2.37 | -2.35 | -2.18 | -1.91 | -1.94 | -1.80 | -0.50 | 0.73 | 0.91 | 3.92 |
| [Pro]- | -2.50 | -2.50 | -2.34 | -2.05 | -2.08 | -1.93 | -0.64 | 0.59 | 0.70 | 3.71 |
| [Ala]- | -2.33 | -2.29 | -2.14 | -1.83 | -1.82 | -1.73 | -0.37 | 0.86 | 0.97 | 3.95 |
| [Lys]- | -2.62 | -2.61 | -2.47 | -2.15 | -2.16 | -2.01 | -0.76 | 0.44 | 0.46 | 3.29 |
| [For]- | -1.94 | -1.85 | -1.60 | -1.37 | -1.31 | -1.36 | 0.13 | 1.36 | 1.68 | 4.84 |
| [Lac]- | -1.95 | -1.88 | -1.67 | -1.43 | -1.41 | -1.46 | 0.03 | 1.28 | 1.57 | 4.84 |
| [2OZ]- | -2.09 | -2.05 | -1.88 | -1.58 | -1.55 | -1.48 | -0.14 | 1.06 | 1.23 | 4.21 |
| [PyZ]- | -1.89 | -1.87 | -1.98 | -1.47 | -1.48 | -1.29 | -0.01 | 1.17 | 1.15 | 3.72 |
| [Glyco]- | -1.67 | -1.57 | -1.35 | -1.12 | -1.07 | -1.20 | 0.37 | 1.61 | 1.99 | 5.31 |
| [EMPO_3_]^-^ | -2.29 | -2.27 | -2.04 | -1.84 | -1.83 | -1.77 | -0.50 | 0.67 | 0.86 | 3.79 |
| [Isole]- | -2.34 | -2.36 | -2.32 | -1.98 | -2.03 | -1.82 | -0.68 | 0.47 | 0.46 | 3.12 |
| [Acryl]- | -1.95 | -1.89 | -1.73 | -1.46 | -1.45 | -1.39 | -0.08 | 1.09 | 1.29 | 4.12 |
| [IsoPMPO_3_]^-^ | -2.29 | -2.29 | -2.16 | -1.90 | -1.92 | -1.79 | -0.59 | 0.55 | 0.61 | 3.32 |
| [Arg]- | -2.03 | -1.94 | -1.82 | -1.48 | -1.42 | -1.42 | -0.12 | 1.03 | 1.11 | 3.81 |
| [Val]- | -2.12 | -2.11 | -2.06 | -1.73 | -1.75 | -1.59 | -0.42 | 0.70 | 0.70 | 3.30 |
| [Abt]- | -2.57 | -2.72 | -2.93 | -2.52 | -2.72 | -2.25 | -1.44 | -0.39 | -0.58 | 1.71 |
| [IMCrb]- | -1.59 | -1.49 | -1.31 | -1.04 | -0.99 | -1.00 | 0.29 | 1.38 | 1.61 | 4.30 |
| [Leu]- | -2.04 | -2.05 | -2.05 | -1.72 | -1.75 | -1.56 | -0.48 | 0.59 | 0.59 | 3.04 |
| [124TAz]- | -1.50 | -1.42 | -1.40 | -0.98 | -0.93 | -0.87 | 0.39 | 1.47 | 1.51 | 3.89 |
| [Met]- | -1.92 | -1.89 | -1.82 | -1.50 | -1.50 | -1.37 | -0.27 | 0.76 | 0.81 | 3.16 |
| [2AIm]- | -1.43 | -1.34 | -1.39 | -0.87 | -0.76 | -0.73 | 0.56 | 1.64 | 1.54 | 3.89 |
| [Ben]- | -1.67 | -1.63 | -1.57 | -1.28 | -1.28 | -1.14 | -0.07 | 0.93 | 1.01 | 3.31 |
| [DOPyrr]^-^ | -1.46 | -1.38 | -1.18 | -0.95 | -0.85 | -0.90 | 0.36 | 1.40 | 1.64 | 4.28 |
| [Glu]- | -1.38 | -1.26 | -1.14 | -0.86 | -0.75 | -0.83 | 0.47 | 1.52 | 1.70 | 4.27 |
| [MCb]^-^ | -1.33 | -1.23 | -0.98 | -0.81 | -0.70 | -0.82 | 0.50 | 1.54 | 1.84 | 4.50 |
| [NMO]- | -1.39 | -1.30 | -1.07 | -0.88 | -0.77 | -0.84 | 0.42 | 1.44 | 1.71 | 4.32 |
| [2MIm]- | -1.47 | -1.43 | -1.60 | -1.03 | -0.97 | -0.82 | 0.30 | 1.30 | 1.06 | 3.14 |
| [Cyst]- | -1.46 | -1.37 | -1.30 | -0.97 | -0.91 | -0.88 | 0.27 | 1.26 | 1.34 | 3.59 |
| [Phe]- | -1.73 | -1.71 | -1.67 | -1.37 | -1.37 | -1.21 | -0.24 | 0.72 | 0.74 | 2.86 |
| [IMZ]- | -1.24 | -1.17 | -1.31 | -0.77 | -0.68 | -0.60 | 0.58 | 1.57 | 1.45 | 3.54 |
| [Thr]- | -1.46 | -1.39 | -1.28 | -0.99 | -0.89 | -0.91 | 0.29 | 1.30 | 1.36 | 3.72 |
| [I3A]- | -1.50 | -1.45 | -1.51 | -1.14 | -1.16 | -1.00 | 0.01 | 0.97 | 0.98 | 3.09 |
| [His]- | -1.20 | -1.08 | -1.06 | -0.72 | -0.65 | -0.68 | 0.53 | 1.55 | 1.64 | 3.99 |
| [3OAcMB]^-^ | -1.50 | -1.46 | -1.35 | -1.12 | -1.05 | -1.01 | 0.07 | 1.02 | 1.16 | 3.46 |
| [Phenolate]- | -1.29 | -1.25 | -1.46 | -0.99 | -0.96 | -0.78 | 0.24 | 1.16 | 1.04 | 2.78 |
| [DEPO_4_]- | -1.56 | -1.52 | -1.33 | -1.16 | -1.08 | -1.09 | 0.01 | 0.96 | 1.13 | 3.52 |
| [Ser]- | -0.93 | -0.80 | -0.73 | -0.43 | -0.30 | -0.47 | 0.90 | 1.93 | 2.10 | 4.65 |
| [2EIm]- | -1.45 | -1.43 | -1.63 | -1.05 | -1.00 | -0.81 | 0.20 | 1.14 | 0.84 | 2.74 |
| [DMO]- | -1.23 | -1.14 | -0.88 | -0.73 | -0.58 | -0.71 | 0.52 | 1.48 | 1.79 | 4.29 |
| [H2PO_3_]- | -0.80 | -0.64 | -0.59 | -0.29 | -0.10 | -0.32 | 1.10 | 2.11 | 2.28 | 4.73 |
| [Aspg]- | -0.86 | -0.69 | -0.58 | -0.30 | -0.13 | -0.31 | 1.03 | 2.02 | 2.23 | 4.67 |
| [DIsoPrPO_4_]- | -1.66 | -1.65 | -1.52 | -1.33 | -1.29 | -1.23 | -0.23 | 0.69 | 0.77 | 3.05 |
| [DMPO_4_]^-^ | -1.21 | -1.12 | -0.91 | -0.75 | -0.62 | -0.73 | 0.47 | 1.41 | 1.64 | 4.01 |
| [DBPO_4_]^-^ | -1.77 | -1.80 | -1.69 | -1.51 | -1.50 | -1.39 | -0.48 | 0.41 | 0.49 | 2.71 |
| [3-(OH)2-MB]^-^ | -0.99 | -0.91 | -0.93 | -0.62 | -0.55 | -0.57 | 0.59 | 1.54 | 1.63 | 3.86 |
| [ClAce]- | -1.01 | -0.90 | -0.80 | -0.57 | -0.44 | -0.52 | 0.66 | 1.58 | 1.76 | 3.95 |
| [ThioSal]- | -1.20 | -1.13 | -1.11 | -0.80 | -0.78 | -0.67 | 0.27 | 1.12 | 1.20 | 3.06 |
| [INDz]- | -1.19 | -1.15 | -1.33 | -0.85 | -0.82 | -0.63 | 0.28 | 1.13 | 0.99 | 2.58 |
| [Try]- | -1.31 | -1.26 | -1.31 | -0.98 | -0.97 | -0.83 | 0.08 | 0.96 | 0.94 | 2.80 |
| [2ClMAcr]- | -1.21 | -1.15 | -1.15 | -0.86 | -0.81 | -0.74 | 0.26 | 1.13 | 1.17 | 3.08 |
| [2BrMAcr]- | -1.15 | -1.08 | -1.04 | -0.75 | -0.75 | -0.63 | 0.27 | 1.10 | 1.18 | 2.99 |
| [2OHMAcr]- | -1.01 | -0.92 | -0.83 | -0.58 | -0.47 | -0.51 | 0.59 | 1.47 | 1.58 | 3.63 |
| [5ATeAz]- | -0.44 | -0.25 | -0.12 | 0.20 | 0.38 | 0.17 | 1.45 | 2.36 | 2.60 | 4.68 |
| [C3C]- | -1.02 | -0.93 | -0.74 | -0.59 | -0.50 | -0.53 | 0.45 | 1.27 | 1.50 | 3.56 |
| [DEABSO_3_]- | -1.41 | -1.41 | -1.22 | -1.07 | -0.98 | -0.97 | -0.05 | 0.76 | 0.88 | 2.999 |
| [Be123TAz]- | -0.93 | -0.85 | -0.84 | -0.50 | -0.43 | -0.37 | 0.54 | 1.34 | 1.36 | 2.97 |
| [IsoPAPSO_3_]- | -1.26 | -1.22 | -0.99 | -0.88 | -0.75 | -0.82 | 0.17 | 0.99 | 1.18 | 3.273 |
| [AceSal]- | -1.03 | -0.97 | -0.90 | -0.67 | -0.58 | -0.55 | 0.38 | 1.18 | 1.28 | 3.13 |
| [DEAPSO_3_]- | -1.31 | -1.29 | -1.09 | -0.94 | -0.84 | -0.86 | 0.10 | 0.91 | 1.05 | 3.172 |
| [56DMBeIm]- | 1.98 | -1.18 | -1.37 | -0.87 | -0.82 | -0.60 | 0.18 | 0.94 | 0.65 | 2.00 |
| [TeAz]- | -0.51 | -0.35 | -0.22 | 0.07 | 0.23 | 0.07 | 1.23 | 2.09 | 2.29 | 4.20 |
| [IsoPABSO_3_]- | -1.13 | -1.07 | -0.84 | -0.72 | -0.58 | -0.68 | 0.34 | 1.16 | 1.37 | 3.456 |
| [DMABSO_3_]- | -1.15 | -1.10 | -0.86 | -0.76 | -0.63 | -0.72 | 0.28 | 1.09 | 1.33 | 3.412 |
| [BAPSO_3_]- | -1.22 | -1.18 | -0.97 | -0.85 | -0.73 | -0.79 | 0.17 | 0.97 | 1.15 | 3.194 |
| [2TFMAcr]- | -0.96 | -0.88 | -0.86 | -0.61 | -0.52 | -0.49 | 0.45 | 1.26 | 1.28 | 3.02 |
| [Tau]- | -0.50 | -0.32 | -0.09 | 0.06 | 0.31 | 0.02 | 1.29 | 2.15 | 2.46 | 4.638 |
| [MeSO_3_]- | -0.46 | -0.30 | -0.01 | 0.07 | 0.31 | -0.01 | 1.27 | 2.13 | 2.55 | 4.828 |
| [HAPSO_3_]- | -1.35 | -1.35 | -1.17 | -1.05 | -0.96 | -0.96 | -0.09 | 0.69 | 0.84 | 2.822 |
| [GluMA]- | -0.41 | -0.31 | -0.32 | -0.05 | 0.07 | -0.07 | 1.10 | 1.97 | 2.12 | 4.23 |
| [Pur]- | -0.83 | -0.73 | -0.72 | -0.34 | -0.25 | -0.22 | 0.67 | 1.43 | 1.32 | 2.70 |
| [BeIm]- | 2.20 | -0.78 | -0.95 | -0.46 | -0.36 | -0.24 | 0.58 | 1.29 | 1.04 | 2.21 |
| [Tyro]- | -0.50 | -0.41 | -0.48 | -0.17 | -0.12 | -0.14 | 0.85 | 1.66 | 1.72 | 3.54 |
| [AspMA]- | -0.18 | -0.05 | -0.04 | 0.23 | 0.38 | 0.18 | 1.38 | 2.22 | 2.40 | 4.44 |
| [H2PO_4_]- | 0.36 | 0.57 | 0.70 | 0.87 | 0.32 | 0.66 | 2.12 | 2.98 | 3.41 | 5.80 |
| [XylSO_3_]- | -0.50 | -0.40 | -0.22 | -0.14 | 0.00 | -0.10 | 0.71 | 1.32 | 1.53 | 2.996 |
| [Gen]- | 0.69 | 0.89 | 0.86 | 1.11 | 1.26 | 0.94 | 2.18 | 2.95 | 3.25 | 5.29 |
| [2NIm]- | -0.20 | -0.04 | 0.01 | 0.26 | 0.46 | 0.34 | 1.17 | 1.74 | 1.75 | 2.79 |
| [EtSO_4_]- | 0.17 | 0.37 | 0.59 | 0.60 | 0.88 | 0.53 | 1.50 | 2.03 | 2.39 | 3.673 |
| [MeSO_4_]- | 0.36 | 0.59 | 0.82 | 0.84 | 1.14 | 0.73 | 1.76 | 2.30 | 2.69 | 3.975 |
| [OMeSO_4_]- | -0.41 | -0.35 | -0.20 | -0.19 | -0.04 | -0.15 | 0.48 | 0.95 | 1.15 | 2.251 |

**Table S4:** COSMO-RS predicted excess enthalpy, H^E^ (kcal/mol) of lignin in different ILs at 363.15 K

| Anion /Cation | [DPrPyrr]+ | [TBA]+ | [TEP]+ | [TBP]+ | [TEA]+ | [EMPyrr]+ | [BMPyrr]+ | [TEMP]+ | [THTeDP]+ | [BDMIM]^+^ | [TEMA]+ | [TBMP]+ |
| --- | --- | --- | --- | --- | --- | --- | --- | --- | --- | --- | --- | --- |
| [Ace]- | -6.49 | -6.38 | -6.41 | -6.33 | -6.40 | -6.21 | -6.09 | -6.06 | -5.89 | -5.96 | -6.03 | -5.86 |
| [PyZ]- | -6.43 | -6.36 | -6.30 | -6.30 | -6.27 | -6.06 | -6.02 | -5.93 | -5.86 | -5.84 | -5.90 | -5.82 |
| [Prop]- | -6.37 | -6.30 | -6.27 | -6.26 | -6.24 | -6.01 | -5.97 | -5.90 | -5.83 | -5.85 | -5.86 | -5.78 |
| [But]- | -6.28 | -6.24 | -6.16 | -6.19 | -6.12 | -5.88 | -5.88 | -5.80 | -5.77 | -5.76 | -5.74 | -5.72 |
| [Valerate]- | -6.19 | -6.18 | -6.08 | -6.13 | -6.02 | -5.77 | -5.80 | -5.71 | -5.72 | -5.69 | -5.64 | -5.66 |
| [Hexa]- | -6.15 | -6.15 | -6.03 | -6.11 | -5.97 | -5.72 | -5.76 | -5.67 | -5.71 | -5.66 | -5.59 | -5.64 |
| [Hep]- | -6.11 | -6.12 | -5.98 | -6.08 | -5.92 | -5.66 | -5.72 | -5.62 | -5.68 | -5.62 | -5.54 | -5.60 |
| [N-MCb]- | -6.10 | -6.00 | -6.03 | -5.96 | -6.02 | -5.81 | -5.72 | -5.70 | -5.54 | -5.60 | -5.66 | -5.51 |
| [Oct]- | -6.08 | -6.10 | -5.95 | -6.06 | -5.89 | -5.63 | -5.69 | -5.59 | -5.66 | -5.59 | -5.51 | -5.58 |
| [Gly]- | -6.05 | -5.95 | -5.98 | -5.91 | -5.96 | -5.76 | -5.66 | -5.65 | -5.50 | -5.55 | -5.61 | -5.47 |
| [Dec]- | -6.01 | -6.04 | -5.88 | -6.00 | -5.81 | -5.56 | -5.63 | -5.52 | -5.61 | -5.53 | -5.44 | -5.53 |
| [Ala]- | -5.89 | -5.85 | -5.81 | -5.81 | -5.77 | -5.54 | -5.51 | -5.47 | -5.41 | -5.41 | -5.41 | -5.37 |
| [Crotonate]- | -5.88 | -5.83 | -5.79 | -5.79 | -5.75 | -5.51 | -5.49 | -5.44 | -5.37 | -5.38 | -5.38 | -5.35 |
| [Pro]- | -5.82 | -5.81 | -5.73 | -5.77 | -5.67 | -5.42 | -5.43 | -5.38 | -5.37 | -5.34 | -5.30 | -5.32 |
| [Abt]- | -5.78 | -5.78 | -5.62 | -5.73 | -5.58 | -5.36 | -5.44 | -5.28 | -5.33 | -5.31 | -5.23 | -5.30 |
| [2OZ]- | -5.75 | -5.68 | -5.67 | -5.64 | -5.64 | -5.42 | -5.36 | -5.34 | -5.22 | -5.25 | -5.29 | -5.20 |
| [2MIm]- | -5.74 | -5.74 | -5.60 | -5.69 | -5.55 | -5.32 | -5.36 | -5.25 | -5.28 | -5.21 | -5.19 | -5.25 |
| [Lys]- | -5.69 | -5.70 | -5.60 | -5.66 | -5.55 | -5.30 | -5.34 | -5.29 | -5.31 | -5.27 | -5.21 | -5.26 |
| [2AIm]- | -5.68 | -5.64 | -5.60 | -5.59 | -5.57 | -5.37 | -5.32 | -5.28 | -5.22 | -5.18 | -5.23 | -5.19 |
| [For]- | -5.67 | -5.51 | -5.63 | -5.47 | -5.65 | -5.51 | -5.31 | -5.33 | -5.04 | -5.19 | -5.34 | -5.05 |
| [Isole]- | -5.58 | -5.59 | -5.47 | -5.55 | -5.41 | -5.16 | -5.22 | -5.13 | -5.17 | -5.13 | -5.05 | -5.13 |
| [IMZ]- | -5.58 | -5.55 | -5.46 | -5.49 | -5.43 | -5.22 | -5.20 | -5.13 | -5.09 | -5.05 | -5.08 | -5.07 |
| [Lac]- | -5.52 | -5.38 | -5.46 | -5.34 | -5.46 | -5.29 | -5.18 | -5.16 | -4.93 | -5.08 | -5.15 | -4.95 |
| [Acryl]- | -5.51 | -5.45 | -5.43 | -5.41 | -5.40 | -5.19 | -5.13 | -5.10 | -5.00 | -5.02 | -5.05 | -4.98 |
| [124TAz]- | -5.48 | -5.44 | -5.40 | -5.39 | -5.37 | -5.18 | -5.11 | -5.08 | -4.99 | -4.96 | -5.04 | -4.97 |
| [2EIm]- | -5.48 | -5.50 | -5.33 | -5.45 | -5.28 | -5.04 | -5.12 | -5.00 | -5.05 | -4.98 | -4.93 | -5.03 |
| [Glyco]- | -5.46 | -5.26 | -5.42 | -5.22 | -5.45 | -5.32 | -5.13 | -5.14 | -4.80 | -5.02 | -5.16 | -4.84 |
| [Val]- | -5.37 | -5.37 | -5.26 | -5.33 | -5.20 | -4.96 | -5.01 | -4.93 | -4.95 | -4.92 | -4.85 | -4.92 |
| [IsoPrMPO_3_]- | -5.31 | -5.33 | -5.22 | -5.30 | -5.15 | -4.89 | -4.94 | -4.89 | -4.92 | -4.87 | -4.79 | -4.88 |
| [Phenolate]- | -5.25 | -5.27 | -5.11 | -5.21 | -5.06 | -4.82 | -4.89 | -4.77 | -4.81 | -4.75 | -4.71 | -4.80 |
| [EMPO_3_]- | -5.20 | -5.20 | -5.13 | -5.17 | -5.07 | -4.81 | -4.83 | -4.81 | -4.79 | -4.76 | -4.72 | -4.75 |
| [Leu]- | -5.16 | -5.18 | -5.05 | -5.14 | -4.99 | -4.75 | -4.81 | -4.73 | -4.76 | -4.73 | -4.65 | -4.74 |
| [Arg]- | -5.03 | -4.99 | -4.95 | -4.95 | -4.92 | -4.74 | -4.75 | -4.71 | -4.66 | -4.67 | -4.65 | -4.65 |
| [Met]- | -5.01 | -5.03 | -4.91 | -4.99 | -4.85 | -4.60 | -4.66 | -4.60 | -4.63 | -4.56 | -4.51 | -4.61 |
| [Ben]- | -4.94 | -4.96 | -4.84 | -4.91 | -4.78 | -4.54 | -4.59 | -4.52 | -4.53 | -4.48 | -4.44 | -4.52 |
| [INDz]- | -4.91 | -4.95 | -4.78 | -4.90 | -4.72 | -4.49 | -4.57 | -4.47 | -4.51 | -4.42 | -4.39 | -4.51 |
| [IMCrb]- | -4.90 | -4.84 | -4.84 | -4.80 | -4.82 | -4.64 | -4.58 | -4.56 | -4.45 | -4.47 | -4.52 | -4.45 |
| [I3A]- | -4.70 | -4.69 | -4.58 | -4.65 | -4.54 | -4.35 | -4.42 | -4.31 | -4.30 | -4.30 | -4.26 | -4.34 |
| [Cyst]- | -4.67 | -4.68 | -4.59 | -4.64 | -4.54 | -4.32 | -4.34 | -4.30 | -4.29 | -4.24 | -4.22 | -4.28 |
| [56DMBeIm]- | -4.65 | -4.70 | -4.52 | -4.65 | -4.45 | -4.23 | -4.34 | -4.22 | -4.27 | -4.20 | -4.14 | -4.29 |
| [Phe]- | -4.63 | -4.67 | -4.53 | -4.63 | -4.46 | -4.22 | -4.30 | -4.23 | -4.27 | -4.21 | -4.14 | -4.27 |
| [Thr]- | -4.55 | -4.55 | -4.49 | -4.52 | -4.43 | -4.20 | -4.21 | -4.20 | -4.17 | -4.16 | -4.12 | -4.16 |
| [DOPyrr]^-^ | -4.55 | -4.51 | -4.51 | -4.48 | -4.46 | -4.24 | -4.20 | -4.21 | -4.09 | -4.12 | -4.14 | -4.11 |
| [His]- | -4.46 | -4.44 | -4.37 | -4.41 | -4.33 | -4.14 | -4.17 | -4.12 | -4.08 | -4.08 | -4.05 | -4.10 |
| [3OAcMB]- | -4.42 | -4.44 | -4.35 | -4.41 | -4.29 | -4.04 | -4.09 | -4.05 | -4.05 | -4.02 | -3.97 | -4.05 |
| [Glu]- | -4.41 | -4.37 | -4.35 | -4.33 | -4.32 | -4.14 | -4.12 | -4.10 | -4.02 | -4.06 | -4.05 | -4.03 |
| [NMO]- | -4.35 | -4.31 | -4.32 | -4.28 | -4.28 | -4.06 | -4.02 | -4.03 | -3.90 | -3.95 | -3.97 | -3.93 |
| [H_2_PO_3_]- | -4.33 | -4.19 | -4.30 | -4.15 | -4.32 | -4.19 | -4.03 | -4.05 | -3.80 | -3.95 | -4.06 | -3.83 |
| [Ser]- | -4.32 | -4.24 | -4.27 | -4.21 | -4.25 | -4.07 | -4.01 | -4.01 | -3.85 | -3.94 | -3.97 | -3.88 |
| [BeIm]- | -4.28 | -4.34 | -4.16 | -4.29 | -4.09 | -3.86 | -3.96 | -3.87 | -3.93 | -3.84 | -3.78 | -3.96 |
| [MCb]- | -4.27 | -4.22 | -4.25 | -4.19 | -4.21 | -4.01 | -3.93 | -3.97 | -3.81 | -3.87 | -3.91 | -3.83 |
| [ThioSal]- | -4.23 | -4.27 | -4.13 | -4.23 | -4.07 | -3.84 | -3.92 | -3.85 | -3.89 | -3.80 | -3.76 | -3.90 |
| [2ClMAcr]- | -4.18 | -4.21 | -4.09 | -4.18 | -4.03 | -3.79 | -3.86 | -3.80 | -3.81 | -3.78 | -3.71 | -3.83 |
| [3-(OH)2-MB]^-^ | -4.17 | -4.14 | -4.08 | -4.09 | -4.05 | -3.85 | -3.87 | -3.81 | -3.73 | -3.79 | -3.76 | -3.78 |
| [Try]- | -4.09 | -4.12 | -3.99 | -4.08 | -3.94 | -3.75 | -3.84 | -3.75 | -3.76 | -3.74 | -3.68 | -3.80 |
| [Aspg]- | -4.05 | -3.99 | -4.01 | -3.95 | -3.99 | -3.83 | -3.77 | -3.77 | -3.66 | -3.71 | -3.74 | -3.67 |
| [2BrMAcr]- | -4.02 | -4.07 | -3.91 | -4.03 | -3.85 | -3.62 | -3.71 | -3.63 | -3.67 | -3.58 | -3.54 | -3.69 |
| [ClAce]- | -4.02 | -4.00 | -3.98 | -3.97 | -3.93 | -3.72 | -3.69 | -3.70 | -3.60 | -3.64 | -3.63 | -3.63 |
| [2OHMAcr]- | -3.95 | -3.96 | -3.89 | -3.93 | -3.83 | -3.60 | -3.63 | -3.61 | -3.56 | -3.56 | -3.53 | -3.59 |
| [DEPO_4_]^-^ | -3.90 | -3.93 | -3.86 | -3.91 | -3.79 | -3.54 | -3.58 | -3.58 | -3.55 | -3.55 | -3.48 | -3.57 |
| [DIsoPrPO_4_]- | -3.90 | -3.94 | -3.84 | -3.91 | -3.76 | -3.52 | -3.58 | -3.56 | -3.56 | -3.56 | -3.46 | -3.58 |
| [DMO]- | -3.88 | -3.84 | -3.87 | -3.81 | -3.82 | -3.60 | -3.55 | -3.60 | -3.44 | -3.51 | -3.52 | -3.49 |
| [Be123TAz]- | -3.83 | -3.89 | -3.76 | -3.85 | -3.68 | -3.46 | -3.52 | -3.48 | -3.50 | -3.41 | -3.39 | -3.53 |
| [DMPO_4_]^-^ | -3.80 | -3.80 | -3.78 | -3.77 | -3.71 | -3.48 | -3.48 | -3.50 | -3.41 | -3.44 | -3.41 | -3.44 |
| [DBPO_4_]^-^ | -3.75 | -3.80 | -3.69 | -3.78 | -3.62 | -3.38 | -3.45 | -3.43 | -3.43 | -3.43 | -3.33 | -3.45 |
| [AceSal]- | -3.72 | -3.76 | -3.65 | -3.73 | -3.58 | -3.35 | -3.42 | -3.38 | -3.39 | -3.35 | -3.29 | -3.42 |
| [Pur]- | -3.63 | -3.72 | -3.55 | -3.68 | -3.46 | -3.22 | -3.32 | -3.28 | -3.35 | -3.22 | -3.16 | -3.37 |
| [GluMA]- | -3.62 | -3.60 | -3.56 | -3.57 | -3.51 | -3.31 | -3.33 | -3.30 | -3.22 | -3.28 | -3.24 | -3.28 |
| [2TFMAcr]- | -3.60 | -3.64 | -3.52 | -3.61 | -3.46 | -3.22 | -3.30 | -3.25 | -3.25 | -3.25 | -3.16 | -3.29 |
| [5ATeAz]- | -3.57 | -3.50 | -3.57 | -3.47 | -3.56 | -3.43 | -3.29 | -3.35 | -3.17 | -3.19 | -3.32 | -3.18 |
| [Tyro]- | -3.46 | -3.47 | -3.37 | -3.43 | -3.32 | -3.14 | -3.21 | -3.14 | -3.10 | -3.13 | -3.07 | -3.17 |
| [TeAz]- | -3.45 | -3.42 | -3.44 | -3.39 | -3.40 | -3.23 | -3.14 | -3.19 | -3.04 | -3.04 | -3.13 | -3.08 |
| [C3C]- | -3.38 | -3.41 | -3.33 | -3.39 | -3.26 | -3.04 | -3.10 | -3.08 | -3.08 | -3.03 | -2.99 | -3.10 |
| [AspMA]- | -3.37 | -3.35 | -3.32 | -3.32 | -3.28 | -3.09 | -3.09 | -3.08 | -2.98 | -3.04 | -3.02 | -3.04 |
| [DEABSO_3_]- | -3.15 | -3.19 | -3.13 | -3.18 | -3.05 | -2.82 | -2.87 | -2.89 | -2.83 | -2.87 | -2.78 | -2.90 |
| [DEAPSO_3_]- | -3.12 | -3.16 | -3.10 | -3.14 | -3.02 | -2.79 | -2.84 | -2.86 | -2.79 | -2.84 | -2.75 | -2.86 |
| [Tau]- | -2.96 | -2.92 | -2.98 | -2.89 | -2.94 | -2.76 | -2.68 | -2.76 | -2.58 | -2.67 | -2.70 | -2.63 |
| [IsoPAPSO_3_]- | -2.89 | -2.93 | -2.87 | -2.91 | -2.79 | -2.56 | -2.61 | -2.64 | -2.58 | -2.62 | -2.54 | -2.65 |
| [IsoPABSO_3_]- | -2.85 | -2.89 | -2.85 | -2.87 | -2.77 | -2.54 | -2.58 | -2.61 | -2.54 | -2.58 | -2.51 | -2.61 |
| [H2PO_4_]- | -2.82 | -2.68 | -2.83 | -2.65 | -2.85 | -2.75 | -2.56 | -2.63 | -2.33 | -2.52 | -2.64 | -2.40 |
| [MeSO_3_]- | -2.79 | -2.72 | -2.82 | -2.70 | -2.79 | -2.62 | -2.50 | -2.60 | -2.33 | -2.49 | -2.55 | -2.43 |
| [BAPSO_3_]- | -2.76 | -2.81 | -2.75 | -2.79 | -2.67 | -2.45 | -2.50 | -2.53 | -2.46 | -2.50 | -2.42 | -2.53 |
| [DMABSO_3_]- | -2.75 | -2.79 | -2.75 | -2.77 | -2.67 | -2.43 | -2.47 | -2.52 | -2.42 | -2.48 | -2.41 | -2.51 |
| [Gen]- | -2.73 | -2.68 | -2.69 | -2.65 | -2.66 | -2.52 | -2.51 | -2.49 | -2.33 | -2.44 | -2.45 | -2.43 |
| [HAPSO_3_]- | -2.73 | -2.78 | -2.71 | -2.76 | -2.63 | -2.41 | -2.47 | -2.49 | -2.44 | -2.48 | -2.39 | -2.51 |
| [2NIm]- | -2.51 | -2.60 | -2.47 | -2.57 | -2.39 | -2.16 | -2.24 | -2.23 | -2.25 | -2.19 | -2.13 | -2.32 |
| [XylSO_3_]- | -1.89 | -1.94 | -1.87 | -1.92 | -1.80 | -1.59 | -1.66 | -1.68 | -1.59 | -1.64 | -1.58 | -1.71 |
| [EtSO_4_]- | -1.04 | -1.06 | -1.08 | -1.05 | -1.01 | -0.81 | -0.82 | -0.90 | -0.71 | -0.84 | -0.80 | -0.88 |
| [MeSO_4_]- | -1.02 | -1.02 | -1.07 | -1.01 | -1.01 | -0.82 | -0.79 | -0.89 | -0.68 | -0.81 | -0.81 | -0.84 |
| [OMeSO_4_]- | -0.93 | -0.96 | -0.94 | -0.94 | -0.87 | -0.69 | -0.75 | -0.79 | -0.59 | -0.78 | -0.70 | -0.80 |

**Table S4 (continued…):** COSMO-RS predicted excess enthalpy, H^E^ (kcal/mol) of lignin in different ILs at 363.15 K

| Anion /Cation | [MTOA]+ | [EDMIM]+ | [HMG]+ | [123TMIM]+ | [EDMPrA]+ | [DEM-2-MeoEA]^+^ | [12MeoEMPyrr]+ | [TMEP]+ | [ADMPy]+ | [TMGH]+ |
| --- | --- | --- | --- | --- | --- | --- | --- | --- | --- | --- |
| [Ace]- | -5.81 | -5.85 | -5.73 | -5.75 | -5.65 | -5.61 | -5.60 | -5.60 | -5.49 | -5.63 |
| [PyZ]- | -5.84 | -5.67 | -5.56 | -5.54 | -5.52 | -5.49 | -5.51 | -5.44 | -5.31 | -5.28 |
| [Prop]- | -5.76 | -5.68 | -5.58 | -5.55 | -5.49 | -5.47 | -5.45 | -5.41 | -5.37 | -5.41 |
| [But]- | -5.71 | -5.58 | -5.48 | -5.43 | -5.39 | -5.38 | -5.36 | -5.29 | -5.29 | -5.29 |
| [Valerate]- | -5.66 | -5.49 | -5.40 | -5.34 | -5.31 | -5.30 | -5.28 | -5.19 | -5.22 | -5.20 |
| [Hexa]- | -5.64 | -5.45 | -5.37 | -5.29 | -5.27 | -5.26 | -5.24 | -5.15 | -5.19 | -5.15 |
| [Hep]- | -5.62 | -5.40 | -5.32 | -5.24 | -5.22 | -5.22 | -5.20 | -5.10 | -5.15 | -5.10 |
| [N-MCb]- | -5.47 | -5.49 | -5.38 | -5.39 | -5.29 | -5.26 | -5.24 | -5.25 | -5.17 | -5.27 |
| [Oct]- | -5.60 | -5.37 | -5.29 | -5.21 | -5.19 | -5.20 | -5.18 | -5.07 | -5.13 | -5.07 |
| [Gly]- | -5.43 | -5.44 | -5.33 | -5.34 | -5.24 | -5.21 | -5.19 | -5.21 | -5.13 | -5.22 |
| [Dec]- | -5.55 | -5.30 | -5.23 | -5.15 | -5.13 | -5.14 | -5.12 | -5.00 | -5.07 | -5.01 |
| [Ala]- | -5.36 | -5.25 | -5.16 | -5.12 | -5.07 | -5.05 | -5.02 | -4.99 | -4.98 | -4.98 |
| [Crotonate]- | -5.32 | -5.21 | -5.12 | -5.07 | -5.04 | -5.02 | -5.00 | -4.95 | -4.93 | -4.93 |
| [Pro]- | -5.31 | -5.16 | -5.07 | -5.01 | -4.97 | -4.96 | -4.93 | -4.88 | -4.92 | -4.88 |
| [Abt]- | -5.33 | -5.08 | -5.01 | -4.94 | -4.95 | -4.96 | -4.97 | -4.81 | -4.82 | -4.73 |
| [2OZ]- | -5.16 | -5.11 | -5.02 | -5.00 | -4.94 | -4.91 | -4.89 | -4.88 | -4.82 | -4.85 |
| [2MIm]- | -5.29 | -4.99 | -4.92 | -4.83 | -4.87 | -4.86 | -4.86 | -4.75 | -4.71 | -4.53 |
| [Lys]- | -5.26 | -5.08 | -5.01 | -4.93 | -4.91 | -4.90 | -4.87 | -4.81 | -4.88 | -4.80 |
| [2AIm]- | -5.20 | -5.04 | -4.94 | -4.92 | -4.89 | -4.86 | -4.86 | -4.83 | -4.74 | -4.69 |
| [For]- | -4.98 | -5.15 | -5.02 | -5.11 | -4.95 | -4.90 | -4.89 | -4.96 | -4.77 | -5.00 |
| [Isole]- | -5.14 | -4.92 | -4.84 | -4.76 | -4.76 | -4.75 | -4.72 | -4.63 | -4.69 | -4.60 |
| [IMZ]- | -5.09 | -4.87 | -4.78 | -4.74 | -4.74 | -4.72 | -4.72 | -4.65 | -4.57 | -4.45 |
| [Lac]- | -4.88 | -4.99 | -4.88 | -4.91 | -4.81 | -4.78 | -4.77 | -4.77 | -4.67 | -4.80 |
| [Acryl]- | -4.95 | -4.87 | -4.79 | -4.75 | -4.71 | -4.68 | -4.66 | -4.65 | -4.59 | -4.60 |
| [124TAz]- | -4.96 | -4.83 | -4.73 | -4.72 | -4.69 | -4.65 | -4.65 | -4.64 | -4.53 | -4.53 |
| [2EIm]- | -5.07 | -4.74 | -4.68 | -4.58 | -4.64 | -4.63 | -4.62 | -4.50 | -4.49 | -4.28 |
| [Glyco]- | -4.75 | -4.99 | -4.86 | -4.95 | -4.80 | -4.76 | -4.76 | -4.80 | -4.62 | -4.85 |
| [Val]- | -4.93 | -4.72 | -4.65 | -4.57 | -4.56 | -4.55 | -4.52 | -4.45 | -4.51 | -4.41 |
| [IsoPrMPO_3_]- | -4.87 | -4.67 | -4.60 | -4.51 | -4.49 | -4.49 | -4.45 | -4.39 | -4.48 | -4.38 |
| [Phenolate]- | -4.84 | -4.52 | -4.46 | -4.35 | -4.42 | -4.40 | -4.39 | -4.27 | -4.27 | -4.05 |
| [EMPO_3_]- | -4.72 | -4.59 | -4.52 | -4.44 | -4.41 | -4.40 | -4.35 | -4.32 | -4.39 | -4.34 |
| [Leu]- | -4.74 | -4.52 | -4.46 | -4.37 | -4.37 | -4.36 | -4.34 | -4.25 | -4.32 | -4.20 |
| [Arg]- | -4.65 | -4.53 | -4.47 | -4.42 | -4.40 | -4.40 | -4.38 | -4.33 | -4.34 | -4.31 |
| [Met]- | -4.61 | -4.36 | -4.31 | -4.20 | -4.23 | -4.22 | -4.19 | -4.12 | -4.18 | -4.05 |
| [Ben]- | -4.53 | -4.27 | -4.22 | -4.10 | -4.16 | -4.14 | -4.12 | -4.04 | -4.06 | -3.92 |
| [INDz]- | -4.54 | -4.20 | -4.15 | -4.03 | -4.12 | -4.10 | -4.11 | -3.99 | -3.98 | -3.75 |
| [IMCrb]- | -4.42 | -4.34 | -4.28 | -4.23 | -4.22 | -4.19 | -4.18 | -4.17 | -4.09 | -4.11 |
| [I3A]- | -4.34 | -4.10 | -4.07 | -3.96 | -4.03 | -4.02 | -4.02 | -3.91 | -3.91 | -3.76 |
| [Cyst]- | -4.28 | -4.06 | -4.01 | -3.92 | -3.94 | -3.92 | -3.90 | -3.86 | -3.87 | -3.77 |
| [56DMBeIm]- | -4.32 | -3.97 | -3.93 | -3.81 | -3.91 | -3.89 | -3.89 | -3.77 | -3.78 | -3.52 |
| [Phe]- | -4.27 | -4.00 | -3.96 | -3.84 | -3.89 | -3.88 | -3.85 | -3.77 | -3.84 | -3.67 |
| [Thr]- | -4.14 | -4.00 | -3.93 | -3.85 | -3.83 | -3.82 | -3.77 | -3.75 | -3.82 | -3.70 |
| [DOPyrr]^-^ | -4.05 | -3.99 | -3.92 | -3.87 | -3.83 | -3.81 | -3.77 | -3.78 | -3.78 | -3.75 |
| [His]- | -4.10 | -3.92 | -3.87 | -3.79 | -3.81 | -3.80 | -3.78 | -3.72 | -3.74 | -3.64 |
| [3OAcMB]- | -4.03 | -3.83 | -3.78 | -3.67 | -3.70 | -3.69 | -3.65 | -3.60 | -3.67 | -3.51 |
| [Glu]- | -4.01 | -3.93 | -3.87 | -3.82 | -3.79 | -3.78 | -3.75 | -3.73 | -3.74 | -3.70 |
| [NMO]- | -3.87 | -3.82 | -3.76 | -3.70 | -3.67 | -3.64 | -3.60 | -3.62 | -3.62 | -3.59 |
| [H_2_PO_3_]- | -3.79 | -3.92 | -3.80 | -3.87 | -3.74 | -3.70 | -3.69 | -3.74 | -3.61 | -3.71 |
| [Ser]- | -3.84 | -3.84 | -3.76 | -3.74 | -3.68 | -3.66 | -3.63 | -3.63 | -3.61 | -3.60 |
| [BeIm]- | -3.98 | -3.61 | -3.57 | -3.44 | -3.55 | -3.53 | -3.52 | -3.41 | -3.44 | -3.15 |
| [MCb]- | -3.76 | -3.77 | -3.69 | -3.67 | -3.60 | -3.57 | -3.53 | -3.57 | -3.55 | -3.57 |
| [ThioSal]- | -3.91 | -3.59 | -3.56 | -3.42 | -3.52 | -3.50 | -3.48 | -3.40 | -3.42 | -3.24 |
| [2ClMAcr]- | -3.82 | -3.58 | -3.53 | -3.42 | -3.47 | -3.45 | -3.41 | -3.35 | -3.41 | -3.22 |
| [3-(OH)2-MB]^-^ | -3.76 | -3.63 | -3.57 | -3.50 | -3.51 | -3.50 | -3.48 | -3.42 | -3.43 | -3.32 |
| [Try]- | -3.81 | -3.55 | -3.53 | -3.41 | -3.49 | -3.48 | -3.47 | -3.37 | -3.40 | -3.24 |
| [Aspg]- | -3.65 | -3.61 | -3.55 | -3.52 | -3.48 | -3.46 | -3.43 | -3.43 | -3.41 | -3.40 |
| [2BrMAcr]- | -3.69 | -3.35 | -3.34 | -3.18 | -3.30 | -3.28 | -3.27 | -3.18 | -3.19 | -3.03 |
| [ClAce]- | -3.60 | -3.50 | -3.43 | -3.38 | -3.35 | -3.32 | -3.28 | -3.29 | -3.31 | -3.22 |
| [2OHMAcr]- | -3.57 | -3.40 | -3.34 | -3.25 | -3.26 | -3.24 | -3.20 | -3.18 | -3.23 | -3.08 |
| [DEPO_4_]^-^ | -3.52 | -3.38 | -3.33 | -3.23 | -3.22 | -3.22 | -3.16 | -3.14 | -3.26 | -3.13 |
| [DIsoPrPO_4_]- | -3.54 | -3.38 | -3.33 | -3.23 | -3.23 | -3.22 | -3.17 | -3.13 | -3.26 | -3.12 |
| [DMO]- | -3.40 | -3.40 | -3.34 | -3.28 | -3.24 | -3.22 | -3.17 | -3.19 | -3.23 | -3.18 |
| [Be123TAz]- | -3.51 | -3.22 | -3.19 | -3.07 | -3.15 | -3.12 | -3.10 | -3.05 | -3.07 | -2.89 |
| [DMPO_4_]^-^ | -3.38 | -3.30 | -3.25 | -3.17 | -3.14 | -3.13 | -3.07 | -3.08 | -3.15 | -3.06 |
| [DBPO_4_]^-^ | -3.41 | -3.25 | -3.20 | -3.10 | -3.10 | -3.10 | -3.05 | -3.00 | -3.14 | -3.00 |
| [AceSal]- | -3.40 | -3.16 | -3.13 | -3.00 | -3.06 | -3.05 | -3.01 | -2.95 | -3.03 | -2.82 |
| [Pur]- | -3.37 | -3.00 | -2.98 | -2.83 | -2.94 | -2.92 | -2.89 | -2.83 | -2.89 | -2.66 |
| [GluMA]- | -3.25 | -3.13 | -3.07 | -3.00 | -3.00 | -3.00 | -2.96 | -2.92 | -2.97 | -2.83 |
| [2TFMAcr]- | -3.28 | -3.05 | -3.01 | -2.89 | -2.94 | -2.92 | -2.88 | -2.83 | -2.92 | -2.70 |
| [5ATeAz]- | -3.14 | -3.16 | -3.07 | -3.11 | -3.03 | -2.99 | -2.98 | -3.06 | -2.91 | -3.01 |
| [Tyro]- | -3.15 | -2.96 | -2.92 | -2.82 | -2.88 | -2.87 | -2.85 | -2.77 | -2.82 | -2.65 |
| [TeAz]- | -3.02 | -2.96 | -2.89 | -2.88 | -2.84 | -2.80 | -2.78 | -2.84 | -2.75 | -2.77 |
| [C3C]- | -3.08 | -2.86 | -2.84 | -2.71 | -2.77 | -2.76 | -2.72 | -2.68 | -2.75 | -2.59 |
| [AspMA]- | -3.01 | -2.91 | -2.85 | -2.78 | -2.78 | -2.77 | -2.73 | -2.71 | -2.75 | -2.62 |
| [DEABSO_3_]- | -2.82 | -2.72 | -2.68 | -2.58 | -2.57 | -2.57 | -2.52 | -2.50 | -2.65 | -2.50 |
| [DEAPSO_3_]- | -2.78 | -2.69 | -2.65 | -2.55 | -2.54 | -2.54 | -2.48 | -2.46 | -2.61 | -2.47 |
| [Tau]- | -2.56 | -2.60 | -2.53 | -2.51 | -2.44 | -2.42 | -2.36 | -2.43 | -2.45 | -2.43 |
| [IsoPAPSO_3_]- | -2.57 | -2.48 | -2.44 | -2.34 | -2.33 | -2.33 | -2.26 | -2.26 | -2.42 | -2.27 |
| [IsoPABSO_3_]- | -2.53 | -2.44 | -2.40 | -2.31 | -2.30 | -2.29 | -2.23 | -2.23 | -2.38 | -2.24 |
| [H2PO_4_]- | -2.32 | -2.52 | -2.43 | -2.50 | -2.35 | -2.33 | -2.30 | -2.38 | -2.26 | -2.40 |
| [MeSO_3_]- | -2.31 | -2.44 | -2.37 | -2.36 | -2.27 | -2.24 | -2.19 | -2.28 | -2.27 | -2.29 |
| [BAPSO_3_]- | -2.45 | -2.36 | -2.32 | -2.23 | -2.22 | -2.22 | -2.15 | -2.15 | -2.31 | -2.16 |
| [DMABSO_3_]- | -2.41 | -2.34 | -2.30 | -2.21 | -2.19 | -2.19 | -2.13 | -2.13 | -2.28 | -2.14 |
| [Gen]- | -2.38 | -2.33 | -2.29 | -2.23 | -2.25 | -2.24 | -2.22 | -2.19 | -2.17 | -2.08 |
| [HAPSO_3_]- | -2.43 | -2.33 | -2.30 | -2.20 | -2.20 | -2.20 | -2.14 | -2.12 | -2.28 | -2.14 |
| [2NIm]- | -2.30 | -2.00 | -1.99 | -1.84 | -1.94 | -1.92 | -1.87 | -1.84 | -1.93 | -1.65 |
| [XylSO_3_]- | -1.63 | -1.50 | -1.49 | -1.36 | -1.41 | -1.40 | -1.35 | -1.34 | -1.46 | -1.28 |
| [EtSO_4_]- | -0.76 | -0.75 | -0.72 | -0.62 | -0.63 | -0.62 | -0.55 | -0.59 | -0.72 | -0.54 |
| [MeSO_4_]- | -0.72 | -0.74 | -0.70 | -0.63 | -0.61 | -0.60 | -0.53 | -0.60 | -0.68 | -0.55 |
| [OMeSO_4_]- | -0.66 | -0.68 | -0.66 | -0.57 | -0.58 | -0.57 | -0.52 | -0.52 | -0.67 | -0.51 |

**Table S4 (continued…):** COSMO-RS predicted excess enthalpy, H^E^ (kcal/mol) of lignin in different ILs at 363.15 K

| Anion /Cation | [TMBP]+ | [BeTPheP]+ | [TEM]+ | [M12MThEPyr]+ | [2ETeG]+ | [B3MPy]+ | [BeBIM]+ | [VBeTMA]+ | [DMA-TMA]+ | [TMA]+ | [BeMIM]+ |
| --- | --- | --- | --- | --- | --- | --- | --- | --- | --- | --- | --- |
| [Ace]- | -5.46 | -5.29 | -5.44 | -5.32 | -5.31 | -5.11 | -5.01 | -4.98 | -5.02 | -5.22 | -4.93 |
| [PyZ]- | -5.36 | -5.11 | -4.97 | -5.24 | -4.72 | -4.94 | -4.90 | -4.87 | -4.93 | -4.97 | -4.75 |
| [Prop]- | -5.34 | -5.22 | -5.23 | -5.20 | -5.14 | -5.00 | -4.94 | -4.89 | -4.91 | -4.95 | -4.83 |
| [But]- | -5.26 | -5.17 | -5.12 | -5.12 | -5.06 | -4.93 | -4.88 | -4.83 | -4.82 | -4.79 | -4.77 |
| [Valerate]- | -5.18 | -5.13 | -5.04 | -5.05 | -4.99 | -4.87 | -4.83 | -4.78 | -4.76 | -4.68 | -4.72 |
| [Hexa]- | -5.15 | -5.12 | -4.99 | -5.02 | -4.96 | -4.84 | -4.82 | -4.76 | -4.73 | -4.63 | -4.70 |
| [Hep]- | -5.11 | -5.09 | -4.94 | -4.99 | -4.92 | -4.81 | -4.79 | -4.73 | -4.70 | -4.59 | -4.67 |
| [N-MCb]- | -5.12 | -4.98 | -5.09 | -4.98 | -4.99 | -4.81 | -4.71 | -4.66 | -4.69 | -4.84 | -4.62 |
| [Oct]- | -5.09 | -5.08 | -4.91 | -4.97 | -4.89 | -4.79 | -4.77 | -4.71 | -4.68 | -4.56 | -4.66 |
| [Gly]- | -5.08 | -4.94 | -5.03 | -4.93 | -4.92 | -4.77 | -4.68 | -4.62 | -4.64 | -4.80 | -4.57 |
| [Dec]- | -5.03 | -5.04 | -4.85 | -4.93 | -4.83 | -4.73 | -4.73 | -4.67 | -4.63 | -4.50 | -4.62 |
| [Ala]- | -4.93 | -4.85 | -4.81 | -4.78 | -4.74 | -4.65 | -4.58 | -4.51 | -4.50 | -4.50 | -4.46 |
| [Crotonate]- | -4.90 | -4.78 | -4.78 | -4.75 | -4.71 | -4.60 | -4.53 | -4.46 | -4.48 | -4.46 | -4.40 |
| [Pro]- | -4.85 | -4.81 | -4.72 | -4.70 | -4.69 | -4.58 | -4.53 | -4.45 | -4.42 | -4.35 | -4.40 |
| [Abt]- | -4.85 | -4.75 | -4.49 | -4.80 | -4.40 | -4.50 | -4.52 | -4.50 | -4.50 | -4.38 | -4.42 |
| [2OZ]- | -4.79 | -4.64 | -4.64 | -4.64 | -4.50 | -4.49 | -4.40 | -4.34 | -4.36 | -4.43 | -4.28 |
| [2MIm]- | -4.77 | -4.56 | -4.19 | -4.63 | -3.95 | -4.41 | -4.39 | -4.32 | -4.36 | -4.21 | -4.21 |
| [Lys]- | -4.82 | -4.81 | -4.67 | -4.67 | -4.65 | -4.58 | -4.55 | -4.45 | -4.41 | -4.28 | -4.41 |
| [2AIm]- | -4.76 | -4.56 | -4.37 | -4.62 | -4.12 | -4.43 | -4.37 | -4.30 | -4.34 | -4.37 | -4.21 |
| [For]- | -4.74 | -4.52 | -4.79 | -4.60 | -4.61 | -4.42 | -4.28 | -4.26 | -4.33 | -4.75 | -4.22 |
| [Isole]- | -4.66 | -4.62 | -4.43 | -4.52 | -4.40 | -4.39 | -4.37 | -4.29 | -4.26 | -4.12 | -4.23 |
| [IMZ]- | -4.61 | -4.38 | -4.10 | -4.47 | -3.84 | -4.26 | -4.22 | -4.15 | -4.20 | -4.18 | -4.04 |
| [Lac]- | -4.64 | -4.48 | -4.61 | -4.53 | -4.49 | -4.34 | -4.24 | -4.23 | -4.26 | -4.43 | -4.18 |
| [Acryl]- | -4.57 | -4.42 | -4.46 | -4.41 | -4.36 | -4.27 | -4.19 | -4.12 | -4.16 | -4.20 | -4.06 |
| [124TAz]- | -4.55 | -4.35 | -4.30 | -4.39 | -4.11 | -4.20 | -4.14 | -4.08 | -4.13 | -4.22 | -3.99 |
| [2EIm]- | -4.55 | -4.37 | -3.93 | -4.42 | -3.70 | -4.21 | -4.21 | -4.14 | -4.16 | -3.95 | -4.03 |
| [Glyco]- | -4.59 | -4.37 | -4.63 | -4.51 | -4.45 | -4.28 | -4.15 | -4.17 | -4.23 | -4.58 | -4.12 |
| [Val]- | -4.47 | -4.43 | -4.26 | -4.31 | -4.23 | -4.21 | -4.18 | -4.09 | -4.07 | -3.93 | -4.04 |
| [IsoPrMPO_3_]- | -4.40 | -4.41 | -4.28 | -4.23 | -4.31 | -4.18 | -4.14 | -4.03 | -3.99 | -3.84 | -3.99 |
| [Phenolate]- | -4.32 | -4.13 | -3.80 | -4.18 | -3.66 | -3.99 | -3.98 | -3.90 | -3.93 | -3.72 | -3.79 |
| [EMPO_3_]- | -4.30 | -4.30 | -4.27 | -4.13 | -4.29 | -4.09 | -4.03 | -3.92 | -3.89 | -3.80 | -3.89 |
| [Leu]- | -4.28 | -4.24 | -4.04 | -4.15 | -4.00 | -4.02 | -4.01 | -3.93 | -3.90 | -3.74 | -3.87 |
| [Arg]- | -4.32 | -4.27 | -4.16 | -4.22 | -4.11 | -4.09 | -4.08 | -4.00 | -3.99 | -3.89 | -3.96 |
| [Met]- | -4.15 | -4.09 | -3.93 | -3.99 | -3.90 | -3.91 | -3.88 | -3.77 | -3.76 | -3.57 | -3.71 |
| [Ben]- | -4.07 | -3.95 | -3.81 | -3.90 | -3.75 | -3.80 | -3.76 | -3.67 | -3.69 | -3.51 | -3.59 |
| [INDz]- | -4.05 | -3.86 | -3.50 | -3.90 | -3.32 | -3.73 | -3.72 | -3.65 | -3.69 | -3.45 | -3.53 |
| [IMCrb]- | -4.09 | -3.94 | -3.98 | -3.95 | -3.87 | -3.82 | -3.75 | -3.69 | -3.73 | -3.75 | -3.62 |
| [I3A]- | -3.96 | -3.81 | -3.60 | -3.86 | -3.51 | -3.69 | -3.69 | -3.63 | -3.66 | -3.46 | -3.54 |
| [Cyst]- | -3.86 | -3.76 | -3.64 | -3.70 | -3.58 | -3.61 | -3.58 | -3.47 | -3.48 | -3.34 | -3.40 |
| [56DMBeIm]- | -3.85 | -3.67 | -3.22 | -3.72 | -3.02 | -3.55 | -3.56 | -3.49 | -3.51 | -3.26 | -3.37 |
| [Phe]- | -3.82 | -3.77 | -3.56 | -3.66 | -3.54 | -3.59 | -3.57 | -3.47 | -3.46 | -3.25 | -3.40 |
| [Thr]- | -3.74 | -3.71 | -3.57 | -3.56 | -3.54 | -3.55 | -3.50 | -3.37 | -3.36 | -3.24 | -3.34 |
| [DOPyrr]^-^ | -3.71 | -3.62 | -3.61 | -3.53 | -3.54 | -3.50 | -3.41 | -3.31 | -3.32 | -3.31 | -3.27 |
| [His]- | -3.74 | -3.65 | -3.51 | -3.60 | -3.45 | -3.51 | -3.50 | -3.40 | -3.41 | -3.26 | -3.34 |
| [3OAcMB]- | -3.62 | -3.55 | -3.39 | -3.43 | -3.35 | -3.43 | -3.36 | -3.25 | -3.25 | -3.07 | -3.20 |
| [Glu]- | -3.69 | -3.63 | -3.55 | -3.57 | -3.49 | -3.50 | -3.45 | -3.36 | -3.36 | -3.29 | -3.33 |
| [NMO]- | -3.55 | -3.44 | -3.49 | -3.37 | -3.44 | -3.36 | -3.25 | -3.15 | -3.17 | -3.16 | -3.11 |
| [H_2_PO_3_]- | -3.56 | -3.39 | -3.45 | -3.45 | -3.27 | -3.33 | -3.23 | -3.17 | -3.23 | -3.52 | -3.14 |
| [Ser]- | -3.56 | -3.46 | -3.42 | -3.42 | -3.32 | -3.34 | -3.27 | -3.20 | -3.21 | -3.25 | -3.16 |
| [BeIm]- | -3.50 | -3.34 | -2.88 | -3.34 | -2.69 | -3.23 | -3.24 | -3.13 | -3.16 | -2.87 | -3.02 |
| [MCb]- | -3.47 | -3.38 | -3.48 | -3.29 | -3.44 | -3.28 | -3.17 | -3.07 | -3.09 | -3.17 | -3.05 |
| [ThioSal]- | -3.46 | -3.33 | -3.14 | -3.29 | -3.07 | -3.21 | -3.19 | -3.08 | -3.11 | -2.88 | -2.99 |
| [2ClMAcr]- | -3.40 | -3.32 | -3.11 | -3.22 | -3.07 | -3.18 | -3.16 | -3.05 | -3.05 | -2.83 | -2.98 |
| [3-(OH)2-MB]^-^ | -3.42 | -3.32 | -3.16 | -3.29 | -3.07 | -3.19 | -3.17 | -3.09 | -3.10 | -2.98 | -3.03 |
| [Try]- | -3.44 | -3.34 | -3.11 | -3.33 | -3.06 | -3.21 | -3.22 | -3.15 | -3.16 | -2.94 | -3.07 |
| [Aspg]- | -3.37 | -3.28 | -3.24 | -3.25 | -3.15 | -3.18 | -3.12 | -3.03 | -3.05 | -3.05 | -3.00 |
| [2BrMAcr]- | -3.25 | -3.12 | -2.95 | -3.08 | -2.91 | -2.98 | -2.98 | -2.87 | -2.90 | -2.64 | -2.77 |
| [ClAce]- | -3.25 | -3.17 | -3.12 | -3.05 | -3.07 | -3.06 | -2.99 | -2.87 | -2.89 | -2.85 | -2.83 |
| [2OHMAcr]- | -3.18 | -3.11 | -2.97 | -2.99 | -2.92 | -3.00 | -2.94 | -2.82 | -2.82 | -2.68 | -2.76 |
| [DEPO_4_]^-^ | -3.16 | -3.18 | -3.08 | -2.96 | -3.14 | -3.03 | -2.96 | -2.83 | -2.79 | -2.63 | -2.81 |
| [DIsoPrPO_4_]- | -3.17 | -3.20 | -3.05 | -2.99 | -3.11 | -3.03 | -2.98 | -2.87 | -2.82 | -2.64 | -2.85 |
| [DMO]- | -3.13 | -3.05 | -3.10 | -2.94 | -3.09 | -2.99 | -2.87 | -2.76 | -2.77 | -2.73 | -2.74 |
| [Be123TAz]- | -3.10 | -3.00 | -2.79 | -2.91 | -2.70 | -2.86 | -2.85 | -2.74 | -2.75 | -2.53 | -2.65 |
| [DMPO_4_]^-^ | -3.05 | -3.02 | -3.00 | -2.85 | -3.02 | -2.92 | -2.83 | -2.70 | -2.69 | -2.59 | -2.68 |
| [DBPO_4_]^-^ | -3.05 | -3.10 | -2.94 | -2.89 | -3.00 | -2.92 | -2.88 | -2.77 | -2.71 | -2.54 | -2.75 |
| [AceSal]- | -3.00 | -2.92 | -2.71 | -2.81 | -2.66 | -2.83 | -2.78 | -2.66 | -2.67 | -2.44 | -2.60 |
| [Pur]- | -2.91 | -2.85 | -2.57 | -2.70 | -2.51 | -2.69 | -2.70 | -2.56 | -2.56 | -2.26 | -2.47 |
| [GluMA]- | -2.93 | -2.87 | -2.70 | -2.78 | -2.65 | -2.76 | -2.72 | -2.62 | -2.62 | -2.47 | -2.58 |
| [2TFMAcr]- | -2.89 | -2.84 | -2.62 | -2.69 | -2.59 | -2.72 | -2.69 | -2.57 | -2.57 | -2.33 | -2.52 |
| [5ATeAz]- | -2.90 | -2.75 | -2.84 | -2.76 | -2.68 | -2.67 | -2.59 | -2.52 | -2.56 | -2.80 | -2.47 |
| [Tyro]- | -2.83 | -2.75 | -2.53 | -2.70 | -2.48 | -2.63 | -2.63 | -2.55 | -2.55 | -2.34 | -2.48 |
| [TeAz]- | -2.73 | -2.60 | -2.64 | -2.55 | -2.51 | -2.51 | -2.44 | -2.33 | -2.37 | -2.50 | -2.27 |
| [C3C]- | -2.72 | -2.66 | -2.56 | -2.52 | -2.55 | -2.58 | -2.52 | -2.39 | -2.40 | -2.20 | -2.34 |
| [AspMA]- | -2.70 | -2.64 | -2.50 | -2.55 | -2.43 | -2.54 | -2.49 | -2.39 | -2.39 | -2.27 | -2.35 |
| [DEABSO_3_]- | -2.53 | -2.59 | -2.47 | -2.35 | -2.53 | -2.45 | -2.40 | -2.27 | -2.21 | -2.03 | -2.27 |
| [DEAPSO_3_]- | -2.49 | -2.55 | -2.43 | -2.31 | -2.50 | -2.42 | -2.36 | -2.23 | -2.17 | -1.99 | -2.22 |
| [Tau]- | -2.34 | -2.30 | -2.35 | -2.16 | -2.33 | -2.24 | -2.14 | -2.02 | -2.02 | -2.04 | -2.01 |
| [IsoPAPSO_3_]- | -2.29 | -2.36 | -2.26 | -2.10 | -2.35 | -2.23 | -2.17 | -2.03 | -1.97 | -1.79 | -2.03 |
| [IsoPABSO_3_]- | -2.25 | -2.31 | -2.22 | -2.06 | -2.31 | -2.19 | -2.13 | -1.99 | -1.93 | -1.75 | -1.99 |
| [H2PO_4_]- | -2.19 | -2.07 | -2.22 | -2.10 | -2.09 | -2.03 | -1.92 | -1.87 | -1.92 | -2.23 | -1.85 |
| [MeSO_3_]- | -2.15 | -2.07 | -2.24 | -1.96 | -2.23 | -2.05 | -1.92 | -1.80 | -1.82 | -1.93 | -1.80 |
| [BAPSO_3_]- | -2.18 | -2.26 | -2.15 | -2.00 | -2.24 | -2.13 | -2.07 | -1.93 | -1.87 | -1.68 | -1.93 |
| [DMABSO_3_]- | -2.15 | -2.21 | -2.14 | -1.95 | -2.24 | -2.10 | -2.03 | -1.88 | -1.83 | -1.65 | -1.89 |
| [Gen]- | -2.17 | -2.06 | -1.98 | -2.06 | -1.90 | -2.00 | -1.96 | -1.90 | -1.93 | -1.84 | -1.84 |
| [HAPSO_3_]- | -2.16 | -2.25 | -2.12 | -1.99 | -2.21 | -2.11 | -2.06 | -1.93 | -1.87 | -1.68 | -1.93 |
| [2NIm]- | -1.92 | -1.87 | -1.57 | -1.69 | -1.49 | -1.80 | -1.77 | -1.60 | -1.62 | -1.31 | -1.54 |
| [XylSO_3_]- | -1.39 | -1.39 | -1.31 | -1.19 | -1.37 | -1.34 | -1.29 | -1.15 | -1.14 | -0.91 | -1.13 |
| [EtSO_4_]- | -0.59 | -0.62 | -0.59 | -0.37 | -0.68 | -0.61 | -0.52 | -0.35 | -0.34 | -0.16 | -0.35 |
| [MeSO_4_]- | -0.56 | -0.56 | -0.59 | -0.34 | -0.64 | -0.57 | -0.47 | -0.29 | -0.31 | -0.20 | -0.30 |
| [OMeSO_4_]- | -0.56 | -0.62 | -0.53 | -0.41 | -0.63 | -0.58 | -0.53 | -0.41 | -0.38 | -0.18 | -0.42 |

**Table S4 (continued…):** COSMO-RS predicted excess enthalpy, H^E^ (kcal/mol) of lignin in different ILs at 363.15 K

| Anion /Cation | [TB(3OH)PrA]+ | [p-AnisEt2NH]+ | [DEMA]+ | [BeTMA]+ | [EMIM]+ | [AMIM]+ | [BeEtoMIM]+ | [BMIM]+ | [EPy]+ | [HMIM]+ | [BPy]+ |
| --- | --- | --- | --- | --- | --- | --- | --- | --- | --- | --- | --- |
| [Ace]- | -4.94 | -4.89 | -4.91 | -4.79 | -4.78 | -4.72 | -4.61 | -4.66 | -4.70 | -4.61 | -4.63 |
| [PyZ]- | -4.48 | -4.48 | -4.32 | -4.67 | -4.45 | -4.38 | -4.48 | -4.40 | -4.48 | -4.39 | -4.47 |
| [Prop]- | -4.82 | -4.76 | -4.70 | -4.68 | -4.61 | -4.57 | -4.54 | -4.54 | -4.56 | -4.52 | -4.53 |
| [But]- | -4.75 | -4.70 | -4.60 | -4.62 | -4.52 | -4.50 | -4.50 | -4.48 | -4.47 | -4.46 | -4.47 |
| [Valerate]- | -4.70 | -4.65 | -4.52 | -4.57 | -4.45 | -4.43 | -4.45 | -4.42 | -4.40 | -4.41 | -4.41 |
| [Hexa]- | -4.68 | -4.63 | -4.48 | -4.55 | -4.41 | -4.41 | -4.44 | -4.39 | -4.37 | -4.39 | -4.39 |
| [Hep]- | -4.65 | -4.60 | -4.44 | -4.52 | -4.37 | -4.37 | -4.42 | -4.36 | -4.33 | -4.36 | -4.36 |
| [N-MCb]- | -4.65 | -4.58 | -4.59 | -4.47 | -4.47 | -4.41 | -4.32 | -4.37 | -4.39 | -4.33 | -4.34 |
| [Oct]- | -4.63 | -4.59 | -4.41 | -4.51 | -4.35 | -4.35 | -4.40 | -4.33 | -4.31 | -4.34 | -4.34 |
| [Gly]- | -4.58 | -4.53 | -4.53 | -4.42 | -4.43 | -4.37 | -4.29 | -4.33 | -4.36 | -4.29 | -4.30 |
| [Dec]- | -4.57 | -4.54 | -4.36 | -4.47 | -4.29 | -4.30 | -4.37 | -4.28 | -4.26 | -4.29 | -4.29 |
| [Ala]- | -4.45 | -4.39 | -4.30 | -4.29 | -4.25 | -4.21 | -4.20 | -4.20 | -4.19 | -4.19 | -4.19 |
| [Crotonate]- | -4.44 | -4.35 | -4.27 | -4.26 | -4.19 | -4.15 | -4.14 | -4.15 | -4.13 | -4.13 | -4.13 |
| [Pro]- | -4.41 | -4.35 | -4.22 | -4.23 | -4.17 | -4.14 | -4.15 | -4.14 | -4.11 | -4.13 | -4.12 |
| [Abt]- | -4.20 | -4.21 | -4.00 | -4.33 | -4.04 | -4.06 | -4.18 | -4.04 | -4.09 | -4.05 | -4.12 |
| [2OZ]- | -4.17 | -4.18 | -4.11 | -4.15 | -4.10 | -4.04 | -4.02 | -4.03 | -4.05 | -4.00 | -4.02 |
| [2MIm]- | -3.75 | -3.84 | -3.56 | -4.12 | -3.85 | -3.80 | -4.00 | -3.88 | -3.91 | -3.90 | -3.97 |
| [Lys]- | -4.41 | -4.35 | -4.20 | -4.24 | -4.17 | -4.15 | -4.19 | -4.16 | -4.12 | -4.17 | -4.15 |
| [2AIm]- | -3.89 | -3.94 | -3.77 | -4.10 | -3.96 | -3.88 | -3.98 | -3.92 | -3.97 | -3.92 | -3.97 |
| [For]- | -4.23 | -4.17 | -4.31 | -4.09 | -4.17 | -4.08 | -3.91 | -4.00 | -4.10 | -3.93 | -3.97 |
| [Isole]- | -4.18 | -4.12 | -3.95 | -4.08 | -3.94 | -3.94 | -4.01 | -3.95 | -3.92 | -3.96 | -3.96 |
| [IMZ]- | -3.65 | -3.70 | -3.47 | -3.95 | -3.75 | -3.67 | -3.82 | -3.73 | -3.79 | -3.74 | -3.81 |
| [Lac]- | -4.18 | -4.14 | -4.16 | -4.06 | -4.06 | -4.00 | -3.91 | -3.94 | -4.00 | -3.89 | -3.92 |
| [Acryl]- | -4.10 | -4.00 | -3.95 | -3.93 | -3.89 | -3.83 | -3.82 | -3.83 | -3.83 | -3.82 | -3.82 |
| [124TAz]- | -3.92 | -3.84 | -3.74 | -3.88 | -3.78 | -3.70 | -3.76 | -3.73 | -3.76 | -3.71 | -3.74 |
| [2EIm]- | -3.50 | -3.64 | -3.32 | -3.95 | -3.65 | -3.61 | -3.83 | -3.69 | -3.72 | -3.73 | -3.80 |
| [Glyco]- | -4.11 | -4.07 | -4.19 | -4.02 | -4.06 | -3.99 | -3.82 | -3.89 | -4.00 | -3.82 | -3.87 |
| [Val]- | -4.02 | -3.95 | -3.79 | -3.89 | -3.78 | -3.76 | -3.83 | -3.78 | -3.75 | -3.80 | -3.79 |
| [IsoPrMPO_3_]- | -4.10 | -3.97 | -3.83 | -3.82 | -3.76 | -3.74 | -3.78 | -3.76 | -3.70 | -3.77 | -3.75 |
| [Phenolate]- | -3.56 | -3.49 | -3.21 | -3.71 | -3.44 | -3.40 | -3.60 | -3.49 | -3.50 | -3.52 | -3.58 |
| [EMPO_3_]- | -4.05 | -3.91 | -3.82 | -3.71 | -3.71 | -3.68 | -3.67 | -3.69 | -3.63 | -3.68 | -3.65 |
| [Leu]- | -3.81 | -3.75 | -3.56 | -3.73 | -3.59 | -3.58 | -3.66 | -3.60 | -3.57 | -3.61 | -3.62 |
| [Arg]- | -3.95 | -3.89 | -3.76 | -3.83 | -3.75 | -3.73 | -3.78 | -3.74 | -3.71 | -3.75 | -3.74 |
| [Met]- | -3.74 | -3.62 | -3.46 | -3.57 | -3.46 | -3.43 | -3.52 | -3.49 | -3.42 | -3.50 | -3.49 |
| [Ben]- | -3.60 | -3.48 | -3.32 | -3.48 | -3.34 | -3.30 | -3.40 | -3.37 | -3.31 | -3.39 | -3.39 |
| [INDz]- | -3.24 | -3.21 | -2.92 | -3.47 | -3.19 | -3.14 | -3.36 | -3.24 | -3.24 | -3.28 | -3.33 |
| [IMCrb]- | -3.68 | -3.57 | -3.52 | -3.51 | -3.47 | -3.41 | -3.42 | -3.43 | -3.41 | -3.42 | -3.42 |
| [I3A]- | -3.43 | -3.36 | -3.16 | -3.48 | -3.25 | -3.23 | -3.39 | -3.30 | -3.28 | -3.33 | -3.36 |
| [Cyst]- | -3.42 | -3.32 | -3.17 | -3.27 | -3.19 | -3.14 | -3.23 | -3.20 | -3.14 | -3.22 | -3.20 |
| [56DMBeIm]- | -2.94 | -2.99 | -2.68 | -3.32 | -3.01 | -2.98 | -3.22 | -3.08 | -3.09 | -3.12 | -3.19 |
| [Phe]- | -3.41 | -3.30 | -3.12 | -3.28 | -3.15 | -3.13 | -3.24 | -3.19 | -3.13 | -3.22 | -3.21 |
| [Thr]- | -3.33 | -3.25 | -3.12 | -3.17 | -3.15 | -3.11 | -3.15 | -3.15 | -3.11 | -3.16 | -3.15 |
| [DOPyrr]^-^ | -3.30 | -3.21 | -3.16 | -3.12 | -3.14 | -3.08 | -3.06 | -3.10 | -3.07 | -3.08 | -3.07 |
| [His]- | -3.35 | -3.24 | -3.09 | -3.23 | -3.13 | -3.09 | -3.19 | -3.15 | -3.10 | -3.17 | -3.16 |
| [3OAcMB]- | -3.18 | -3.09 | -2.93 | -3.06 | -3.00 | -2.96 | -3.02 | -3.02 | -2.97 | -3.03 | -3.03 |
| [Glu]- | -3.32 | -3.25 | -3.15 | -3.19 | -3.16 | -3.12 | -3.15 | -3.15 | -3.12 | -3.15 | -3.14 |
| [NMO]- | -3.21 | -3.09 | -3.06 | -2.96 | -3.01 | -2.94 | -2.91 | -2.97 | -2.93 | -2.96 | -2.94 |
| [H_2_PO_3_]- | -3.04 | -2.99 | -3.01 | -3.02 | -3.07 | -2.97 | -2.91 | -2.94 | -3.04 | -2.90 | -2.96 |
| [Ser]- | -3.13 | -3.06 | -2.99 | -3.02 | -3.02 | -2.96 | -2.96 | -2.97 | -2.98 | -2.95 | -2.97 |
| [BeIm]- | -2.65 | -2.66 | -2.33 | -2.96 | -2.68 | -2.64 | -2.90 | -2.77 | -2.74 | -2.83 | -2.87 |
| [MCb]- | -3.20 | -3.05 | -3.06 | -2.89 | -2.97 | -2.90 | -2.84 | -2.91 | -2.88 | -2.88 | -2.86 |
| [ThioSal]- | -3.02 | -2.86 | -2.67 | -2.90 | -2.74 | -2.70 | -2.85 | -2.81 | -2.72 | -2.85 | -2.83 |
| [2ClMAcr]- | -2.96 | -2.85 | -2.65 | -2.87 | -2.74 | -2.71 | -2.83 | -2.78 | -2.73 | -2.82 | -2.82 |
| [3-(OH)2-MB]^-^ | -2.95 | -2.88 | -2.72 | -2.93 | -2.81 | -2.77 | -2.87 | -2.82 | -2.81 | -2.83 | -2.85 |
| [Try]- | -3.01 | -2.92 | -2.72 | -3.00 | -2.81 | -2.80 | -2.95 | -2.87 | -2.82 | -2.91 | -2.91 |
| [Aspg]- | -2.98 | -2.91 | -2.84 | -2.87 | -2.87 | -2.81 | -2.83 | -2.84 | -2.83 | -2.84 | -2.83 |
| [2BrMAcr]- | -2.86 | -2.69 | -2.50 | -2.70 | -2.52 | -2.49 | -2.65 | -2.60 | -2.48 | -2.65 | -2.61 |
| [ClAce]- | -2.90 | -2.76 | -2.68 | -2.69 | -2.70 | -2.63 | -2.66 | -2.68 | -2.65 | -2.69 | -2.68 |
| [2OHMAcr]- | -2.78 | -2.66 | -2.52 | -2.63 | -2.59 | -2.53 | -2.61 | -2.61 | -2.55 | -2.63 | -2.61 |
| [DEPO_4_]^-^ | -2.96 | -2.81 | -2.70 | -2.64 | -2.66 | -2.63 | -2.65 | -2.67 | -2.59 | -2.68 | -2.64 |
| [DIsoPrPO_4_]- | -2.95 | -2.82 | -2.68 | -2.68 | -2.66 | -2.64 | -2.69 | -2.68 | -2.60 | -2.69 | -2.66 |
| [DMO]- | -2.86 | -2.73 | -2.71 | -2.58 | -2.67 | -2.59 | -2.55 | -2.63 | -2.58 | -2.61 | -2.59 |
| [Be123TAz]- | -2.67 | -2.53 | -2.32 | -2.56 | -2.42 | -2.37 | -2.53 | -2.47 | -2.39 | -2.51 | -2.49 |
| [DMPO_4_]^-^ | -2.84 | -2.68 | -2.61 | -2.51 | -2.57 | -2.52 | -2.51 | -2.56 | -2.50 | -2.56 | -2.53 |
| [DBPO_4_]^-^ | -2.85 | -2.72 | -2.59 | -2.59 | -2.56 | -2.55 | -2.59 | -2.58 | -2.51 | -2.59 | -2.56 |
| [AceSal]- | -2.58 | -2.46 | -2.28 | -2.48 | -2.39 | -2.35 | -2.47 | -2.45 | -2.38 | -2.48 | -2.47 |
| [Pur]- | -2.51 | -2.35 | -2.11 | -2.37 | -2.22 | -2.18 | -2.38 | -2.31 | -2.19 | -2.37 | -2.32 |
| [GluMA]- | -2.55 | -2.45 | -2.29 | -2.45 | -2.39 | -2.35 | -2.43 | -2.40 | -2.37 | -2.41 | -2.42 |
| [2TFMAcr]- | -2.50 | -2.39 | -2.20 | -2.40 | -2.30 | -2.27 | -2.39 | -2.36 | -2.30 | -2.40 | -2.39 |
| [5ATeAz]- | -2.55 | -2.42 | -2.43 | -2.36 | -2.41 | -2.31 | -2.29 | -2.33 | -2.34 | -2.30 | -2.29 |
| [Tyro]- | -2.45 | -2.34 | -2.15 | -2.40 | -2.25 | -2.23 | -2.37 | -2.30 | -2.26 | -2.33 | -2.34 |
| [TeAz]- | -2.40 | -2.23 | -2.20 | -2.16 | -2.20 | -2.10 | -2.12 | -2.15 | -2.12 | -2.14 | -2.11 |
| [C3C]- | -2.47 | -2.30 | -2.18 | -2.22 | -2.19 | -2.15 | -2.22 | -2.24 | -2.14 | -2.27 | -2.23 |
| [AspMA]- | -2.34 | -2.23 | -2.09 | -2.22 | -2.18 | -2.13 | -2.21 | -2.19 | -2.16 | -2.20 | -2.21 |
| [DEABSO_3_]- | -2.26 | -2.27 | -2.14 | -2.09 | -2.12 | -2.09 | -2.13 | -2.14 | -2.06 | -2.15 | -2.11 |
| [DEAPSO_3_]- | -2.24 | -2.23 | -2.11 | -2.05 | -2.09 | -2.06 | -2.09 | -2.11 | -2.02 | -2.12 | -2.08 |
| [Tau]- | -2.13 | -2.01 | -2.00 | -1.85 | -1.99 | -1.90 | -1.86 | -1.94 | -1.90 | -1.92 | -1.89 |
| [IsoPAPSO_3_]- | -2.19 | -2.06 | -1.96 | -1.85 | -1.92 | -1.88 | -1.91 | -1.94 | -1.84 | -1.94 | -1.89 |
| [IsoPABSO_3_]- | -2.14 | -2.01 | -1.91 | -1.81 | -1.88 | -1.84 | -1.86 | -1.90 | -1.80 | -1.90 | -1.85 |
| [H2PO_4_]- | -1.93 | -1.81 | -1.87 | -1.74 | -1.85 | -1.76 | -1.67 | -1.73 | -1.80 | -1.68 | -1.70 |
| [MeSO_3_]- | -2.04 | -1.84 | -1.90 | -1.64 | -1.83 | -1.73 | -1.63 | -1.75 | -1.72 | -1.71 | -1.69 |
| [BAPSO_3_]- | -2.10 | -1.96 | -1.85 | -1.76 | -1.82 | -1.79 | -1.81 | -1.84 | -1.74 | -1.85 | -1.80 |
| [DMABSO_3_]- | -2.10 | -1.93 | -1.84 | -1.71 | -1.79 | -1.75 | -1.76 | -1.81 | -1.70 | -1.81 | -1.76 |
| [Gen]- | -1.88 | -1.75 | -1.63 | -1.77 | -1.70 | -1.65 | -1.73 | -1.71 | -1.69 | -1.72 | -1.73 |
| [HAPSO_3_]- | -2.08 | -1.95 | -1.84 | -1.76 | -1.80 | -1.78 | -1.81 | -1.83 | -1.73 | -1.84 | -1.79 |
| [2NIm]- | -1.50 | -1.38 | -1.14 | -1.43 | -1.35 | -1.30 | -1.49 | -1.46 | -1.33 | -1.52 | -1.48 |
| [XylSO_3_]- | -1.37 | -1.14 | -1.03 | -1.00 | -1.03 | -0.99 | -1.06 | -1.09 | -0.96 | -1.12 | -1.06 |
| [EtSO_4_]- | -0.71 | -0.40 | -0.33 | -0.20 | -0.34 | -0.27 | -0.31 | -0.38 | -0.25 | -0.40 | -0.33 |
| [MeSO_4_]- | -0.67 | -0.35 | -0.30 | -0.14 | -0.31 | -0.23 | -0.25 | -0.33 | -0.23 | -0.35 | -0.28 |
| [OMeSO_4_]- | -0.65 | -0.43 | -0.34 | -0.29 | -0.35 | -0.33 | -0.36 | -0.39 | -0.30 | -0.40 | -0.36 |

**Table S4 (continued…):** COSMO-RS predicted excess enthalpy, H^E^ (kcal/mol) of lignin in different ILs at 363.15 K

| Anion /Cation | [13DMIM]+ | [OMIM]+ | [FurEt2NH]+ | [MPyrr]+ | [VanEt2NH]+ | [DMBA]+ | [DMOA]+ | [(3OH)PrTMA]+ | [ACh]+ | [TMEOHP]+ | [Ch]+ |
| --- | --- | --- | --- | --- | --- | --- | --- | --- | --- | --- | --- |
| [Ace]- | -4.76 | -4.58 | -4.41 | -4.51 | -4.26 | -4.25 | -4.14 | -4.26 | -4.14 | -4.16 | -3.93 |
| [PyZ]- | -4.38 | -4.38 | -3.97 | -3.79 | -3.69 | -3.57 | -3.54 | -3.72 | -3.95 | -3.57 | -3.28 |
| [Prop]- | -4.55 | -4.50 | -4.28 | -4.27 | -4.12 | -4.09 | -4.02 | -4.03 | -3.99 | -3.92 | -3.67 |
| [But]- | -4.45 | -4.45 | -4.23 | -4.17 | -4.06 | -4.02 | -3.97 | -3.92 | -3.91 | -3.82 | -3.57 |
| [Valerate]- | -4.37 | -4.40 | -4.18 | -4.10 | -4.02 | -3.97 | -3.93 | -3.85 | -3.86 | -3.75 | -3.51 |
| [Hexa]- | -4.33 | -4.38 | -4.16 | -4.06 | -4.00 | -3.94 | -3.91 | -3.81 | -3.85 | -3.71 | -3.48 |
| [Hep]- | -4.29 | -4.35 | -4.13 | -4.02 | -3.98 | -3.91 | -3.88 | -3.78 | -3.83 | -3.68 | -3.45 |
| [N-MCb]- | -4.44 | -4.29 | -4.12 | -4.20 | -3.98 | -3.96 | -3.86 | -3.96 | -3.82 | -3.87 | -3.63 |
| [Oct]- | -4.26 | -4.33 | -4.12 | -4.00 | -3.96 | -3.89 | -3.86 | -3.76 | -3.83 | -3.66 | -3.44 |
| [Gly]- | -4.40 | -4.26 | -4.07 | -4.13 | -3.92 | -3.89 | -3.81 | -3.88 | -3.76 | -3.79 | -3.54 |
| [Dec]- | -4.21 | -4.28 | -4.07 | -3.95 | -3.92 | -3.84 | -3.82 | -3.72 | -3.81 | -3.61 | -3.40 |
| [Ala]- | -4.18 | -4.17 | -3.94 | -3.89 | -3.78 | -3.73 | -3.69 | -3.64 | -3.58 | -3.55 | -3.28 |
| [Crotonate]- | -4.12 | -4.12 | -3.89 | -3.87 | -3.74 | -3.70 | -3.66 | -3.63 | -3.56 | -3.54 | -3.28 |
| [Pro]- | -4.09 | -4.12 | -3.89 | -3.82 | -3.74 | -3.68 | -3.65 | -3.55 | -3.52 | -3.47 | -3.21 |
| [Abt]- | -3.96 | -4.05 | -3.77 | -3.55 | -3.53 | -3.46 | -3.43 | -3.48 | -3.75 | -3.33 | -3.16 |
| [2OZ]- | -4.05 | -3.98 | -3.72 | -3.71 | -3.54 | -3.49 | -3.43 | -3.45 | -3.48 | -3.35 | -3.08 |
| [2MIm]- | -3.73 | -3.91 | -3.37 | -3.02 | -3.04 | -2.88 | -2.92 | -2.93 | -3.36 | -2.76 | -2.45 |
| [Lys]- | -4.07 | -4.16 | -3.93 | -3.82 | -3.78 | -3.72 | -3.71 | -3.55 | -3.50 | -3.48 | -3.21 |
| [2AIm]- | -3.89 | -3.92 | -3.47 | -3.29 | -3.21 | -3.07 | -3.07 | -3.16 | -3.39 | -3.03 | -2.72 |
| [For]- | -4.21 | -3.89 | -3.74 | -3.97 | -3.60 | -3.62 | -3.48 | -3.76 | -3.57 | -3.66 | -3.49 |
| [Isole]- | -3.85 | -3.96 | -3.69 | -3.54 | -3.52 | -3.45 | -3.45 | -3.32 | -3.37 | -3.23 | -2.98 |
| [IMZ]- | -3.65 | -3.75 | -3.22 | -2.95 | -2.92 | -2.76 | -2.79 | -2.89 | -3.22 | -2.74 | -2.42 |
| [Lac]- | -4.05 | -3.86 | -3.71 | -3.80 | -3.59 | -3.56 | -3.46 | -3.62 | -3.51 | -3.53 | -3.32 |
| [Acryl]- | -3.82 | -3.80 | -3.56 | -3.56 | -3.41 | -3.38 | -3.34 | -3.35 | -3.25 | -3.26 | -3.00 |
| [124TAz]- | -3.72 | -3.71 | -3.38 | -3.31 | -3.18 | -3.09 | -3.07 | -3.19 | -3.20 | -3.09 | -2.81 |
| [2EIm]- | -3.52 | -3.75 | -3.18 | -2.80 | -2.85 | -2.69 | -2.74 | -2.69 | -3.20 | -2.52 | -2.22 |
| [Glyco]- | -4.10 | -3.77 | -3.67 | -3.86 | -3.54 | -3.54 | -3.38 | -3.71 | -3.55 | -3.61 | -3.45 |
| [Val]- | -3.68 | -3.80 | -3.52 | -3.38 | -3.36 | -3.30 | -3.30 | -3.16 | -3.16 | -3.08 | -2.82 |
| [IsoPrMPO_3_]- | -3.66 | -3.77 | -3.54 | -3.45 | -3.42 | -3.36 | -3.36 | -3.20 | -3.09 | -3.13 | -2.88 |
| [Phenolate]- | -3.30 | -3.54 | -3.04 | -2.68 | -2.76 | -2.61 | -2.67 | -2.70 | -2.94 | -2.56 | -2.27 |
| [EMPO_3_]- | -3.62 | -3.67 | -3.49 | -3.47 | -3.40 | -3.35 | -3.32 | -3.19 | -3.02 | -3.14 | -2.88 |
| [Leu]- | -3.49 | -3.61 | -3.33 | -3.16 | -3.16 | -3.08 | -3.09 | -2.97 | -3.02 | -2.88 | -2.64 |
| [Arg]- | -3.67 | -3.75 | -3.53 | -3.43 | -3.41 | -3.34 | -3.34 | -3.25 | -3.20 | -3.19 | -2.94 |
| [Met]- | -3.34 | -3.51 | -3.21 | -3.08 | -3.06 | -3.00 | -3.01 | -2.86 | -2.84 | -2.79 | -2.51 |
| [Ben]- | -3.21 | -3.40 | -3.07 | -2.92 | -2.90 | -2.84 | -2.88 | -2.75 | -2.75 | -2.66 | -2.38 |
| [INDz]- | -3.04 | -3.30 | -2.79 | -2.45 | -2.51 | -2.37 | -2.43 | -2.42 | -2.73 | -2.28 | -2.00 |
| [IMCrb]- | -3.40 | -3.42 | -3.17 | -3.18 | -3.05 | -3.01 | -2.99 | -2.99 | -2.88 | -2.92 | -2.66 |
| [I3A]- | -3.14 | -3.35 | -3.00 | -2.78 | -2.81 | -2.74 | -2.78 | -2.70 | -2.84 | -2.60 | -2.37 |
| [Cyst]- | -3.07 | -3.23 | -2.92 | -2.80 | -2.77 | -2.70 | -2.73 | -2.60 | -2.56 | -2.53 | -2.23 |
| [56DMBeIm]- | -2.87 | -3.15 | -2.60 | -2.22 | -2.29 | -0.31 | -2.20 | -2.18 | -2.62 | -2.03 | -1.77 |
| [Phe]- | -3.02 | -3.23 | -2.92 | -2.75 | -2.76 | -2.70 | -2.73 | -2.56 | -2.57 | -2.49 | -2.24 |
| [Thr]- | -3.05 | -3.16 | -2.86 | -2.75 | -2.72 | -2.64 | -2.66 | -2.52 | -2.47 | -2.45 | -2.17 |
| [DOPyrr]^-^ | -3.07 | -3.07 | -2.81 | -2.81 | -2.69 | -2.64 | -2.60 | -2.57 | -2.47 | -2.51 | -2.23 |
| [His]- | -3.02 | -3.19 | -2.89 | -2.74 | -2.75 | -2.68 | -2.72 | -2.60 | -2.59 | -2.53 | -2.28 |
| [3OAcMB]- | -2.88 | -3.04 | -2.70 | -2.57 | -2.56 | -2.48 | -2.49 | -2.37 | -2.36 | -2.30 | -2.03 |
| [Glu]- | -3.09 | -3.14 | -2.89 | -2.82 | -2.78 | -2.70 | -2.70 | -2.64 | -2.57 | -2.58 | -2.33 |
| [NMO]- | -2.93 | -2.95 | -2.70 | -2.73 | -2.60 | -2.57 | -2.54 | -2.49 | -2.32 | -2.44 | -2.17 |
| [H_2_PO_3_]- | -3.08 | -2.89 | -2.61 | -2.66 | -2.47 | -2.38 | -2.32 | -2.60 | -2.52 | -2.50 | -2.31 |
| [Ser]- | -2.97 | -2.94 | -2.69 | -2.64 | -2.56 | -2.48 | -2.46 | -2.50 | -2.44 | -2.42 | -2.17 |
| [BeIm]- | -2.52 | -2.86 | -2.28 | -1.88 | -2.00 | -0.22 | -1.93 | -1.83 | -2.21 | -1.70 | -1.40 |
| [MCb]- | -2.92 | -2.86 | -2.67 | -2.75 | -2.60 | -2.56 | -2.52 | -2.52 | -2.27 | -2.47 | -2.23 |
| [ThioSal]- | -2.60 | -2.87 | -2.49 | -2.30 | -2.33 | -2.26 | -2.32 | -2.18 | -2.19 | -2.09 | -1.82 |
| [2ClMAcr]- | -2.61 | -2.84 | -2.48 | -2.27 | -2.32 | -2.24 | -2.30 | -2.13 | -2.15 | -2.05 | -1.79 |
| [3-(OH)2-MB]^-^ | -2.71 | -2.84 | -2.52 | -2.35 | -2.37 | -2.27 | -2.30 | -2.26 | -2.30 | -2.16 | -1.92 |
| [Try]- | -2.69 | -2.93 | -2.60 | -2.38 | -2.43 | -2.36 | -2.42 | -2.30 | -2.39 | -2.21 | -2.00 |
| [Aspg]- | -2.81 | -2.84 | -2.56 | -2.52 | -2.45 | -2.38 | -2.39 | -2.37 | -2.28 | -2.30 | -2.05 |
| [2BrMAcr]- | -2.36 | -2.68 | -2.32 | -2.14 | -2.18 | -2.12 | -2.20 | -2.00 | -1.98 | -1.92 | -1.65 |
| [ClAce]- | -2.61 | -2.69 | -2.40 | -2.33 | -2.28 | -2.22 | -2.24 | -2.16 | -2.02 | -2.10 | -1.83 |
| [2OHMAcr]- | -2.47 | -2.64 | -2.29 | -2.16 | -2.16 | -2.08 | -2.12 | -1.99 | -1.93 | -1.92 | -1.64 |
| [DEPO_4_]^-^ | -2.55 | -2.68 | -2.46 | -2.40 | -2.38 | -2.32 | -2.33 | -2.14 | -1.97 | -2.11 | -1.85 |
| [DIsoPrPO_4_]- | -2.55 | -2.69 | -2.47 | -2.38 | -2.38 | -2.32 | -2.33 | -2.14 | -2.03 | -2.10 | -1.87 |
| [DMO]- | -2.59 | -2.59 | -2.37 | -2.42 | -2.29 | -2.26 | -2.22 | -2.15 | -1.95 | -2.12 | -1.85 |
| [Be123TAz]- | -2.28 | -2.53 | -2.17 | -1.97 | -2.01 | -1.92 | -1.98 | -1.84 | -1.85 | -1.77 | -1.48 |
| [DMPO_4_]^-^ | -2.48 | -2.56 | -2.32 | -2.31 | -2.25 | -2.20 | -2.20 | -2.07 | -1.85 | -2.03 | -1.76 |
| [DBPO_4_]^-^ | -2.46 | -2.59 | -2.39 | -2.30 | -2.30 | -2.24 | -2.24 | -2.07 | -1.97 | -2.03 | -1.81 |
| [AceSal]- | -2.26 | -2.50 | -2.11 | -1.93 | -1.97 | -1.88 | -1.93 | -1.79 | -1.81 | -1.71 | -1.45 |
| [Pur]- | -2.05 | -2.41 | -2.00 | -1.76 | -1.85 | -1.75 | -1.85 | -1.60 | -1.61 | -1.54 | -1.23 |
| [GluMA]- | -2.29 | -2.42 | -2.12 | -1.96 | -2.00 | -1.89 | -1.91 | -1.85 | -1.84 | -1.78 | -1.53 |
| [2TFMAcr]- | -2.17 | -2.42 | -2.05 | -1.85 | -1.90 | -1.84 | -1.91 | -1.71 | -1.70 | -1.63 | -1.38 |
| [5ATeAz]- | -2.40 | -2.29 | -2.07 | -2.16 | -1.98 | -1.90 | -1.88 | -2.03 | -1.83 | -1.98 | -1.75 |
| [Tyro]- | -2.14 | -2.35 | -2.04 | -1.83 | -1.89 | -1.81 | -1.86 | -1.76 | -1.80 | -1.68 | -1.47 |
| [TeAz]- | -2.14 | -2.14 | -1.87 | -1.91 | -1.78 | -1.70 | -1.71 | -1.76 | -1.56 | -1.72 | -1.45 |
| [C3C]- | -2.07 | -2.29 | -1.98 | -1.89 | -1.89 | -1.84 | -1.89 | -1.70 | -1.57 | -1.65 | -1.39 |
| [AspMA]- | -2.09 | -2.21 | -1.90 | -1.76 | -1.79 | -1.68 | -1.71 | -1.66 | -1.62 | -1.59 | -1.33 |
| [DEABSO_3_]- | -2.02 | -2.15 | -1.96 | -1.89 | -1.85 | -1.83 | -1.84 | -1.53 | -1.49 | -1.49 | -1.25 |
| [DEAPSO_3_]- | -1.98 | -2.11 | -1.92 | -1.86 | -1.82 | -1.80 | -1.80 | -1.50 | -1.44 | -1.46 | -1.22 |
| [Tau]- | -1.93 | -1.91 | -1.71 | -1.76 | -1.65 | -1.60 | -1.59 | -1.51 | -1.27 | -1.49 | -1.23 |
| [IsoPAPSO_3_]- | -1.81 | -1.94 | -1.77 | -1.73 | -1.72 | -1.67 | -1.68 | -1.43 | -1.24 | -1.42 | -1.18 |
| [IsoPABSO_3_]- | -1.77 | -1.90 | -1.72 | -1.68 | -1.67 | -1.62 | -1.64 | -1.39 | -1.19 | -1.38 | -1.13 |
| [H2PO_4_]- | -1.88 | -1.66 | -1.49 | -1.62 | -1.45 | -1.36 | -1.30 | -1.57 | -1.35 | -1.51 | -1.50 |
| [MeSO_3_]- | -1.79 | -1.69 | -1.53 | -1.67 | -1.51 | -1.48 | -1.43 | -1.46 | -1.11 | -1.45 | -1.20 |
| [BAPSO_3_]- | -1.71 | -1.85 | -1.68 | -1.63 | -1.64 | -1.58 | -1.60 | -1.35 | -1.16 | -1.35 | -1.11 |
| [DMABSO_3_]- | -1.68 | -1.81 | -1.64 | -1.62 | -1.61 | -1.56 | -1.57 | -1.34 | -1.10 | -1.34 | -1.09 |
| [Gen]- | -1.62 | -1.73 | -1.46 | -1.35 | -1.38 | -1.28 | -1.32 | -1.33 | -1.27 | -1.26 | -1.04 |
| [HAPSO_3_]- | -1.70 | -1.84 | -1.68 | -1.63 | -1.63 | -1.58 | -1.59 | -1.35 | -1.18 | -1.34 | -1.12 |
| [2NIm]- | -1.19 | -1.56 | -1.08 | -0.83 | -0.96 | -0.83 | -0.96 | -0.70 | -0.72 | -0.63 | -0.32 |
| [XylSO_3_]- | -0.90 | -1.13 | -0.90 | -0.83 | -0.88 | -0.83 | -0.89 | -0.66 | -0.43 | -0.65 | -0.43 |
| [EtSO_4_]- | -0.22 | -0.41 | -0.18 | -0.15 | -0.22 | -0.13 | -0.21 | -0.01 | 0.36 | -0.03 | 0.23 |
| [MeSO_4_]- | -0.21 | -0.36 | -0.12 | -0.13 | -0.17 | -0.07 | -0.15 | -0.01 | 0.40 | -0.03 | 0.24 |
| [OMeSO_4_]- | -0.26 | -0.41 | -0.26 | -0.21 | -0.27 | -0.21 | -0.23 | -0.09 | 0.17 | -0.10 | 0.07 |

**Table S4 (continued…):** COSMO-RS predicted excess enthalpy, H^E^ (kcal/mol) of lignin in different ILs at 363.15 K

| Anion /Cation | [BeDM(2OH)EA]+ | [EUrMIM]+ | [12HyE3MIM]+ | [CPOPMIM]+ | [D(OH)EDMA]+ | [DMUr]+ | [Tris(2OH)EMA]+ | [Gun]+ |
| --- | --- | --- | --- | --- | --- | --- | --- | --- |
| [Ace]- | -3.64 | -3.53 | -3.54 | -3.34 | -2.77 | -2.22 | -1.77 | -0.77 |
| [PyZ]- | -3.13 | -3.23 | -2.91 | -2.66 | -1.91 | -1.52 | -0.87 | -0.42 |
| [Prop]- | -3.51 | -3.42 | -3.34 | -3.20 | -2.55 | -2.12 | -1.60 | -0.83 |
| [But]- | -3.47 | -3.38 | -3.27 | -3.16 | -2.50 | -2.12 | -1.59 | -0.86 |
| [Valerate]- | -3.43 | -3.35 | -3.22 | -3.13 | -2.47 | -2.13 | -1.58 | -0.86 |
| [Hexa]- | -3.42 | -3.34 | -3.19 | -3.12 | -2.46 | -2.14 | -1.59 | -0.87 |
| [Hep]- | -3.40 | -3.32 | -3.17 | -3.11 | -2.45 | -2.15 | -1.59 | -0.87 |
| [N-MCb]- | -3.38 | -3.29 | -3.30 | -3.12 | -2.54 | -2.00 | -1.60 | -0.59 |
| [Oct]- | -3.39 | -3.31 | -3.15 | -3.10 | -2.45 | -2.15 | -1.59 | -0.86 |
| [Gly]- | -3.31 | -3.26 | -3.23 | -3.03 | -2.43 | -1.92 | -1.48 | -0.61 |
| [Dec]- | -3.36 | -3.29 | -3.11 | -3.07 | -2.43 | -2.14 | -1.59 | -0.84 |
| [Ala]- | -3.18 | -3.14 | -3.03 | -2.89 | -2.22 | -1.82 | -1.33 | -0.64 |
| [Crotonate]- | -3.16 | -3.07 | -3.00 | -2.87 | -2.24 | -1.79 | -1.37 | -0.61 |
| [Pro]- | -3.15 | -3.11 | -2.98 | -2.88 | -2.21 | -1.86 | -1.36 | -0.63 |
| [Abt]- | -3.11 | -3.10 | -2.80 | -2.72 | -2.14 | -1.94 | -1.31 | -0.94 |
| [2OZ]- | -2.92 | -2.98 | -2.83 | -2.61 | -2.01 | -1.63 | -1.14 | -0.38 |
| [2MIm]- | -2.48 | -2.80 | -2.24 | -1.98 | -1.17 | -1.15 | -0.30 | -0.38 |
| [Lys]- | -3.21 | -3.19 | -3.04 | -2.94 | -2.26 | -1.97 | -1.44 | -0.87 |
| [2AIm]- | -2.61 | -2.86 | -2.47 | -2.18 | -1.44 | -1.18 | -0.50 | -0.18 |
| [For]- | -3.03 | -2.96 | -3.06 | -2.78 | -2.36 | -1.73 | -1.39 | -0.30 |
| [Isole]- | -2.99 | -2.95 | -2.76 | -2.67 | -2.01 | -1.76 | -1.20 | -0.75 |
| [IMZ]- | -2.37 | -2.65 | -2.18 | -1.90 | -1.12 | -0.92 | -0.22 | -0.17 |
| [Lac]- | -3.07 | -2.98 | -3.00 | -2.85 | -2.32 | -1.81 | -1.45 | -0.44 |
| [Acryl]- | -2.86 | -2.77 | -2.72 | -2.57 | -1.97 | -1.47 | -1.11 | -0.44 |
| [124TAz]- | -2.64 | -2.68 | -2.49 | -2.27 | -1.63 | -1.18 | -0.71 | -0.27 |
| [2EIm]- | -2.31 | -2.68 | -2.05 | -1.80 | -1.02 | -1.15 | -0.23 | -0.44 |
| [Glyco]- | -3.04 | -2.95 | -3.05 | -2.83 | -2.41 | -1.81 | -1.51 | -0.30 |
| [Val]- | -2.83 | -2.80 | -2.62 | -2.53 | -1.87 | -1.62 | -1.07 | -0.69 |
| [IsoPrMPO_3_]- | -2.88 | -2.77 | -2.69 | -2.66 | -1.98 | -1.63 | -1.20 | -0.57 |
| [Phenolate]- | -2.34 | -2.43 | -2.04 | -1.87 | -1.10 | -0.93 | -0.27 | -0.53 |
| [EMPO_3_]- | -2.84 | -2.71 | -2.70 | -2.66 | -2.02 | -1.60 | -1.26 | -0.48 |
| [Leu]- | -2.66 | -2.65 | -2.44 | -2.35 | -1.71 | -1.48 | -0.95 | -0.60 |
| [Arg]- | -2.94 | -2.92 | -2.78 | -2.69 | -2.09 | -1.79 | -1.35 | -0.85 |
| [Met]- | -2.55 | -2.53 | -2.34 | -2.25 | -1.62 | -1.33 | -0.88 | -0.53 |
| [Ben]- | -2.41 | -2.37 | -2.18 | -2.07 | -1.46 | -1.16 | -0.73 | -0.46 |
| [INDz]- | -2.09 | -2.27 | -1.80 | -1.60 | -0.92 | -0.86 | -0.19 | -0.51 |
| [IMCrb]- | -2.54 | -2.48 | -2.42 | -2.27 | -1.72 | -1.24 | -0.94 | -0.25 |
| [I3A]- | -2.42 | -2.44 | -2.16 | -2.05 | -1.48 | -1.30 | -0.78 | -0.71 |
| [Cyst]- | -2.25 | -2.27 | -2.07 | -1.95 | -1.33 | -1.03 | -0.60 | -0.37 |
| [56DMBeIm]- | -1.88 | -2.20 | -1.61 | -1.38 | -0.73 | -0.88 | -0.08 | -0.52 |
| [Phe]- | -2.30 | -2.28 | -2.07 | -1.99 | -1.40 | -1.17 | -0.72 | -0.48 |
| [Thr]- | -2.19 | -2.23 | -2.07 | -1.94 | -1.29 | -1.01 | -0.57 | -0.20 |
| [DOPyrr]^-^ | -2.15 | -2.16 | -2.08 | -1.93 | -1.35 | -0.95 | -0.64 | 0.02 |
| [His]- | -2.32 | -2.31 | -2.12 | -2.05 | -1.45 | -1.17 | -0.76 | -0.49 |
| [3OAcMB]- | -2.06 | -2.08 | -1.91 | -1.80 | -1.19 | -0.90 | -0.53 | -0.07 |
| [Glu]- | -2.31 | -2.31 | -2.20 | -2.08 | -1.50 | -1.15 | -0.80 | -0.28 |
| [NMO]- | -2.08 | -2.05 | -2.02 | -1.88 | -1.33 | -0.87 | -0.63 | 0.05 |
| [H_2_PO_3_]- | -2.01 | -2.06 | -2.03 | -1.80 | -1.28 | -0.74 | -0.44 | 0.34 |
| [Ser]- | -2.08 | -2.10 | -2.00 | -1.87 | -1.28 | -0.88 | -0.54 | 0.03 |
| [BeIm]- | -1.57 | -1.89 | -1.31 | -1.09 | -0.41 | -0.55 | 0.18 | -0.49 |
| [MCb]- | -2.08 | -2.00 | -2.05 | -1.93 | -1.39 | -0.87 | -0.68 | 0.05 |
| [ThioSal]- | -1.90 | -1.90 | -1.65 | -1.55 | -0.98 | -0.74 | -0.35 | -0.30 |
| [2ClMAcr]- | -1.88 | -1.88 | -1.66 | -1.57 | -0.96 | -0.79 | -0.34 | -0.43 |
| [3-(OH)2-MB]^-^ | -1.95 | -1.97 | -1.77 | -1.67 | -1.07 | -0.82 | -0.40 | -0.21 |
| [Try]- | -2.09 | -2.10 | -1.83 | -1.75 | -1.23 | -1.10 | -0.62 | -0.65 |
| [Aspg]- | -1.99 | -2.03 | -1.91 | -1.77 | -1.21 | -0.83 | -0.52 | -0.01 |
| [2BrMAcr]- | -1.76 | -1.72 | -1.48 | -1.42 | -0.88 | -0.67 | -0.30 | -0.38 |
| [ClAce]- | -1.81 | -1.78 | -1.71 | -1.58 | -0.99 | -0.59 | -0.32 | -0.06 |
| [2OHMAcr]- | -1.69 | -1.71 | -1.55 | -1.43 | -0.82 | -0.52 | -0.20 | 0.02 |
| [DEPO_4_]^-^ | -1.90 | -1.84 | -1.79 | -1.76 | -1.16 | -0.85 | -0.57 | -0.06 |
| [DIsoPrPO_4_]- | -1.93 | -1.88 | -1.79 | -1.76 | -1.19 | -0.94 | -0.61 | -0.14 |
| [DMO]- | -1.78 | -1.78 | -1.77 | -1.63 | -1.10 | -0.68 | -0.48 | 0.13 |
| [Be123TAz]- | -1.59 | -1.64 | -1.36 | -1.25 | -0.68 | -0.50 | -0.09 | -0.22 |
| [DMPO_4_]^-^ | -1.76 | -1.71 | -1.70 | -1.63 | -1.04 | -0.65 | -0.43 | 0.10 |
| [DBPO_4_]^-^ | -1.87 | -1.82 | -1.73 | -1.72 | -1.17 | -0.92 | -0.61 | -0.10 |
| [AceSal]- | -1.53 | -1.59 | -1.37 | -1.25 | -0.70 | -0.49 | -0.14 | 0.08 |
| [Pur]- | -1.41 | -1.48 | -1.16 | -1.07 | -0.46 | -0.37 | 0.10 | -0.25 |
| [GluMA]- | -1.59 | -1.61 | -1.44 | -1.37 | -0.78 | -0.52 | -0.19 | 0.10 |
| [2TFMAcr]- | -1.50 | -1.52 | -1.31 | -1.22 | -0.64 | -0.52 | -0.09 | -0.25 |
| [5ATeAz]- | -1.53 | -1.55 | -1.52 | -1.31 | -0.88 | -0.34 | -0.17 | 0.39 |
| [Tyro]- | -1.56 | -1.58 | -1.35 | -1.29 | -0.77 | -0.63 | -0.23 | -0.27 |
| [TeAz]- | -1.31 | -1.33 | -1.26 | -1.09 | -0.61 | -0.09 | 0.05 | 0.39 |
| [C3C]- | -1.46 | -1.43 | -1.33 | -1.25 | -0.75 | -0.44 | -0.23 | 0.20 |
| [AspMA]- | -1.38 | -1.41 | -1.25 | -1.17 | -0.58 | -0.29 | -0.01 | 0.26 |
| [DEABSO_3_]- | -1.34 | -1.47 | -1.27 | -1.20 | -0.70 | -0.59 | -0.25 | 0.08 |
| [DEAPSO_3_]- | -1.30 | -1.43 | -1.24 | -1.17 | -0.66 | -0.54 | -0.21 | 0.11 |
| [Tau]- | -1.17 | -1.22 | -1.20 | -1.06 | -0.57 | -0.16 | -0.03 | 0.43 |
| [IsoPAPSO_3_]- | -1.28 | -1.28 | -1.21 | -1.19 | -0.67 | -0.43 | -0.23 | 0.10 |
| [IsoPABSO_3_]- | -1.22 | -1.23 | -1.17 | -1.13 | -0.61 | -0.36 | -0.16 | 0.16 |
| [H2PO_4_]- | -1.06 | -1.02 | -1.12 | -0.99 | -0.58 | 0.04 | 0.05 | 1.03 |
| [MeSO_3_]- | -1.07 | -1.01 | -1.14 | -1.03 | -0.57 | 0.00 | -0.02 | 0.64 |
| [BAPSO_3_]- | -1.21 | -1.20 | -1.14 | -1.13 | -0.61 | -0.38 | -0.18 | 0.14 |
| [DMABSO_3_]- | -1.18 | -1.15 | -1.13 | -1.12 | -0.60 | -0.31 | -0.16 | 0.18 |
| [Gen]- | -1.07 | -1.05 | -0.93 | -0.89 | -0.41 | -0.09 | 0.08 | 0.31 |
| [HAPSO_3_]- | -1.22 | -1.22 | -1.15 | -1.14 | -0.64 | -0.43 | -0.23 | 0.11 |
| [2NIm]- | -0.53 | -0.72 | -0.37 | -0.26 | 0.30 | 0.37 | 0.66 | 0.21 |
| [XylSO_3_]- | -0.56 | -0.51 | -0.46 | -0.46 | -0.04 | 0.21 | 0.28 | 0.44 |
| [EtSO_4_]- | 0.09 | 0.16 | 0.10 | 0.05 | 0.55 | 0.98 | 0.80 | 0.92 |
| [MeSO_4_]- | 0.16 | 0.23 | 0.14 | 0.11 | 0.60 | 1.15 | 0.89 | 1.07 |
| [OMeSO_4_]- | -0.06 | -0.02 | -0.01 | -0.07 | 0.29 | 0.54 | 0.47 | 0.63 |

**Table S5:** Summary of molecular dynamics simulations (different lignin polymers in [TBA][Gly] IL)

| Lignin/[TBA][Gly] system | Mol. Weight (g/mol) | IL molecules | Simulations box size (Å) | | |
| --- | --- | --- | --- | --- | --- |
|  |  |  | X | Y | Z |
| Lignin (DP = 4) | 646.68 | 456 | 64.19 | 64.19 | 64.19 |
| Lignin (DP = 4) | 772.89 | 456 | 64.05 | 64.05 | 64.05 |
| Lignin (DP = 6) | 1069.11 | 456 | 64.07 | 64.07 | 64.07 |
| Lignin (DP = 7) | 1265.31 | 456 | 64.27 | 64.27 | 64.27 |
| Lignin (DP = 8) | 1499.55 | 456 | 64.04 | 64.04 | 64.04 |
| Lignin (DP = 9) | 1711.75 | 456 | 64.25 | 64.25 | 64.25 |
| Lignin (DP = 12) | 1978.09 | 456 | 64.30 | 64.30 | 64.30 |
| Lignin (DP = 12) | 2338.40 | 456 | 64.39 | 64.39 | 64.39 |
| Lignin (DP = 13) | 2594.65 | 456 | 64.28 | 64.28 | 64.28 |
| Lignin (DP = 17) | 3427.49 | 456 | 64.39 | 64.39 | 64.39 |
| Lignin (DP = 26) | 4963.08 | 456 | 64.50 | 64.50 | 64.50 |

**Table S6:** Building of lignin polymer with different monomeric units and linkages using LigninBuilder tool. Chemical cstructure of lignin polymer is shown in Figure S1.

| ID | Monomers | Bond | | Linkage |
| --- | --- | --- | --- | --- |
|  | G |  |  |  |
|  | G | 1 | 2 | 5‒5 |
|  | S | 2 | 3 | β‒O‒4 |
|  | S | 3 | 4 | β‒O‒4 |
|  | S | 4 | 5 | β‒O‒4 |
|  | G | 5 | 6 | β‒O‒4 |
|  | G | 6 | 7 | 5‒5 |
|  | S | 7 | 15 | β‒O‒4 |
|  | G | 8 | 9 | β‒5 |
|  | G | 9 | 10 | β‒β |
|  | G | 10 | 11 | 4‒O‒5 |
|  | G | 11 | 12 | β‒O‒4 |
|  | G | 12 | 13 | β‒O‒4 |
|  | S | 13 | 14 | 4‒O‒5 |
|  | P | 6 | 8 | β‒O‒4 |
|  | G | 15 | 16 | β‒O‒4 |
|  | S | 16 | 17 | β‒β |
|  | G | 17 | 18 | 4‒O‒5 |
|  | G | 18 | 19 | β‒5 |
|  | P | 19 | 23 | β‒β |
|  | G | 20 | 21 | 4‒O‒5 |
|  | G | 21 | 22 | β‒1 |
|  | G | 18 | 20 | β‒O‒4 |
|  | G | 16 | 24 | 5‒5 |
|  | S | 24 | 25 | β‒O‒4 |
|  | G | 25 | 26 | β‒O‒4 |

**Table S7:** Lignin polymer with DP of 4 (646.68 g/mol) with different monomeric units and linkages using LigninBuilder tool

| ID | Monomers | Bond | | Linkage |
| --- | --- | --- | --- | --- |
|  | G |  |  |  |
|  | G | 1 | 2 | β‒1 |
|  | H | 2 | 3 | 4‒O‒5 |
|  | G | 2 | 4 | 5‒5 |

**Table S8:** Lignin polymer with DP of 4 (772.89 g/mol) with different monomeric units and linkages using LigninBuilder tool

| ID | Monomers | Bond | | Linkage |
| --- | --- | --- | --- | --- |
|  | G |  |  |  |
|  | S | 1 | 2 | β‒O‒4 |
|  | G | 2 | 3 | β‒O‒4 |
|  | S | 3 | 4 | β‒1 |

**Table S9:** Lignin polymer with DP of 6 (1069.10 g/mol) with different monomeric units and linkages using LigninBuilder tool

| ID | Monomers | Bond | | Linkage |
| --- | --- | --- | --- | --- |
|  | G |  |  |  |
|  | G | 1 | 2 | β‒1 |
|  | G | 2 | 3 | β‒O‒4 |
|  | G | 2 | 4 | 4‒O‒5 |
|  | G | 3 | 5 | β‒O‒4 |
|  | G | 3 | 6 | 5‒5 |

**Table S10:** Lignin polymer with DP of 7 (1265.31 g/mol) with different monomeric units and linkages using LigninBuilder tool

| ID | Monomers | Bond | | Linkage |
| --- | --- | --- | --- | --- |
|  | G |  |  |  |
|  | G | 1 | 2 | β‒1 |
|  | G | 2 | 3 | β‒O‒4 |
|  | G | 3 | 4 | β‒O‒4 |
|  | G | 3 | 5 | 5‒5 |
|  | G | 4 | 6 | 5‒5 |
|  | G | 4 | 7 | β‒O‒4 |

**Table S11:** Lignin polymer with DP of 8 (1499.55 g/mol) with different monomeric units and linkages using LigninBuilder tool

| ID | Monomers | Bond | | Linkage |
| --- | --- | --- | --- | --- |
|  | G |  |  |  |
|  | G | 1 | 2 | β‒O‒4 |
|  | G | 2 | 3 | β‒O‒4 |
|  | G | 2 | 4 | 5‒5 |
|  | G | 3 | 5 | β‒5 |
|  | G | 3 | 6 | 5‒5 |
|  | G | 4 | 7 | β‒O‒4 |
|  | G | 4 | 8 | β‒O‒4 |

**Table S12:** Lignin polymer with DP of 9 (1711.75 g/mol) with different monomeric units and linkages using LigninBuilder tool

| ID | Monomers | Bond | | Linkage |
| --- | --- | --- | --- | --- |
|  | G |  |  |  |
|  | G | 1 | 2 | 4‒O‒5 |
|  | S | 2 | 3 | β‒O‒4 |
|  | S | 3 | 4 | β‒β |
|  | G | 4 | 5 | β‒O‒4 |
|  | G | 5 | 6 | β‒O‒4 |
|  | S | 6 | 7 | 4‒O‒5 |
|  | G | 7 | 8 | β‒1 |
|  | G | 8 | 9 | 5‒5 |

**Table S13:** Lignin polymer with DP of 12 (1978. 1 g/mol) with different monomeric units and linkages using LigninBuilder tool

| ID | Monomers | Bond | | Linkage |
| --- | --- | --- | --- | --- |
|  | H |  |  |  |
|  | H | 1 | 2 | β‒O‒4 |
|  | H | 2 | 3 | β‒O‒4 |
|  | H | 3 | 4 | β‒O‒4 |
|  | H | 4 | 5 | β‒O‒4 |
|  | H | 5 | 6 | β‒O‒4 |
|  | H | 6 | 7 | β‒O‒4 |
|  | H | 7 | 8 | β‒O‒4 |
|  | H | 8 | 9 | β‒O‒4 |
|  | H | 9 | 10 | β‒O‒4 |
|  | H | 10 | 11 | β‒O‒4 |
|  | H | 11 | 12 | β‒O‒4 |

**Table S14:** Lignin polymer with DP of 12 (2338.40 g/mol) with different monomeric units and linkages using LigninBuilder tool

| ID | Monomers | Bond | | Linkage |
| --- | --- | --- | --- | --- |
|  | G |  |  |  |
|  | G | 1 | 2 | β‒O‒4 |
|  | G | 2 | 3 | β‒O‒4 |
|  | G | 3 | 4 | β‒O‒4 |
|  | G | 4 | 5 | β‒O‒4 |
|  | G | 5 | 6 | β‒O‒4 |
|  | G | 6 | 7 | β‒O‒4 |
|  | G | 7 | 8 | β‒O‒4 |
|  | G | 8 | 9 | β‒O‒4 |
|  | G | 9 | 10 | β‒O‒4 |
|  | G | 10 | 11 | β‒O‒4 |
|  | G | 11 | 12 | β‒O‒4 |

**Table S15:** Lignin polymer with DP of 13 (2594.65 g/mol) with different monomeric units and linkages using LigninBuilder tool

| ID | Monomers | Bond | | Linkage |
| --- | --- | --- | --- | --- |
|  | G |  |  |  |
|  | G | 1 | 2 | 5‒5 |
|  | S | 2 | 3 | β‒O‒4 |
|  | S | 3 | 4 | β‒O‒4 |
|  | S | 4 | 5 | β‒O‒4 |
|  | G | 5 | 6 | β‒O‒4 |
|  | S | 6 | 7 | β‒O‒4 |
|  | G | 7 | 8 | β‒5 |
|  | G | 8 | 9 | β‒β |
|  | S | 9 | 10 | 4‒O‒5 |
|  | G | 10 | 11 | β‒O‒4 |
|  | G | 11 | 12 | β‒O‒4 |
|  | S | 12 | 13 | 4‒O‒5 |

**Table S16:** Lignin polymer with DP of 17 (4963.1 g/mol) with different monomeric units and linkages using LigninBuilder tool

| ID | Monomers | Bond | | Linkage |
| --- | --- | --- | --- | --- |
|  | G |  |  |  |
|  | S | 1 | 2 | β‒O‒4 |
|  | S | 2 | 3 | β‒O‒4 |
|  | S | 3 | 4 | β‒O‒4 |
|  | S | 4 | 5 | β‒O‒4 |
|  | G | 5 | 6 | β‒O‒4 |
|  | G | 6 | 7 | 4‒O‒5 |
|  | S | 7 | 8 | β‒O‒4 |
|  | S | 8 | 9 | β‒1 |
|  | G | 9 | 10 | β‒O‒4 |
|  | G | 10 | 11 | 4‒O‒5 |
|  | S | 11 | 12 | β‒1 |
|  | G | 12 | 13 | β‒O‒4 |
|  | S | 13 | 14 | β‒O‒4 |
|  | G | 14 | 15 | β‒O‒4 |
|  | S | 15 | 16 | 4‒O‒5 |
|  | G | 16 | 17 | β‒O‒4 |

**Table S17:** Summary of molecular dynamics simulations (lignin (DP = 26) in different ILs)

| Lignin/IL system | IL molecules | Lignin molecule | Simulations box size (Å) | | |
| --- | --- | --- | --- | --- | --- |
|  |  |  | X | Y | Z |
| [TBA][Ala] | 434 | 1 | 64.84 | 64.84 | 64.84 |
| [TBA][Gly] | 456 | 1 | 64.50 | 64.50 | 64.50 |
| [TBA][Ace] | 462 | 1 | 64.11 | 64.11 | 64.11 |
| [TBA][Val] | 392 | 1 | 64.35 | 64.35 | 64.35 |
| [Ch][Lys] | 703 | 1 | 65.37 | 65.37 | 65.37 |

**Figure S1.** Correlation between the COSMO-RS calculated activity coefficient *vs* experimental lignin solubility in different ILs.

|  |  |
| --- | --- |

**Figure S2.** Experimental solubility of lignin in nine ILs and the COSMO-RS predicted results (a) *ln*(*γ*), and (b) *H^E^*) for the six lignin models.

|  |
| --- |
|  |

**Figure S3.** Effect of alkyl chain lengths of anions (a) and cations (b) on the dissolution of lignin. Origin2020 SR1 used to draw the heatmaps (https://www.originlab.com/2020).

**Figure S4:** COSMO-RS measured sigma potentials of lignin model structures at 363.15 K. COSMOTherm *version* 19.0.1 was used to calculate the sigma potentials (https://www.3ds.com/products-services/biovia/products/molecular-modeling-simulation/solvation-chemistry/biovia-cosmotherm/).

**Figure S5.** Quantum chemical-based predicted interaction energies between anion and cation of ILs. The interaction energies are calculated at B3LYP/6-311++G(d,p) level of theory and basis set using *Gaussin*09 program.

|  |  |
| --- | --- |

**Figure S6.** Dissociation constant (pK_a_) of lignin (a) and anion (b) moieties. The pK_a_ of lignin and anions were measured by using ChemAxon tool.

**Figure S7.** Chemical structure of lignin (DP = 26). The lignin molecule is composed of all possible linkages such as β‒β, β‒1, β‒5, 5‒5, β‒O‒4, α‒O‒4, and 4‒O‒5 linkages.

| 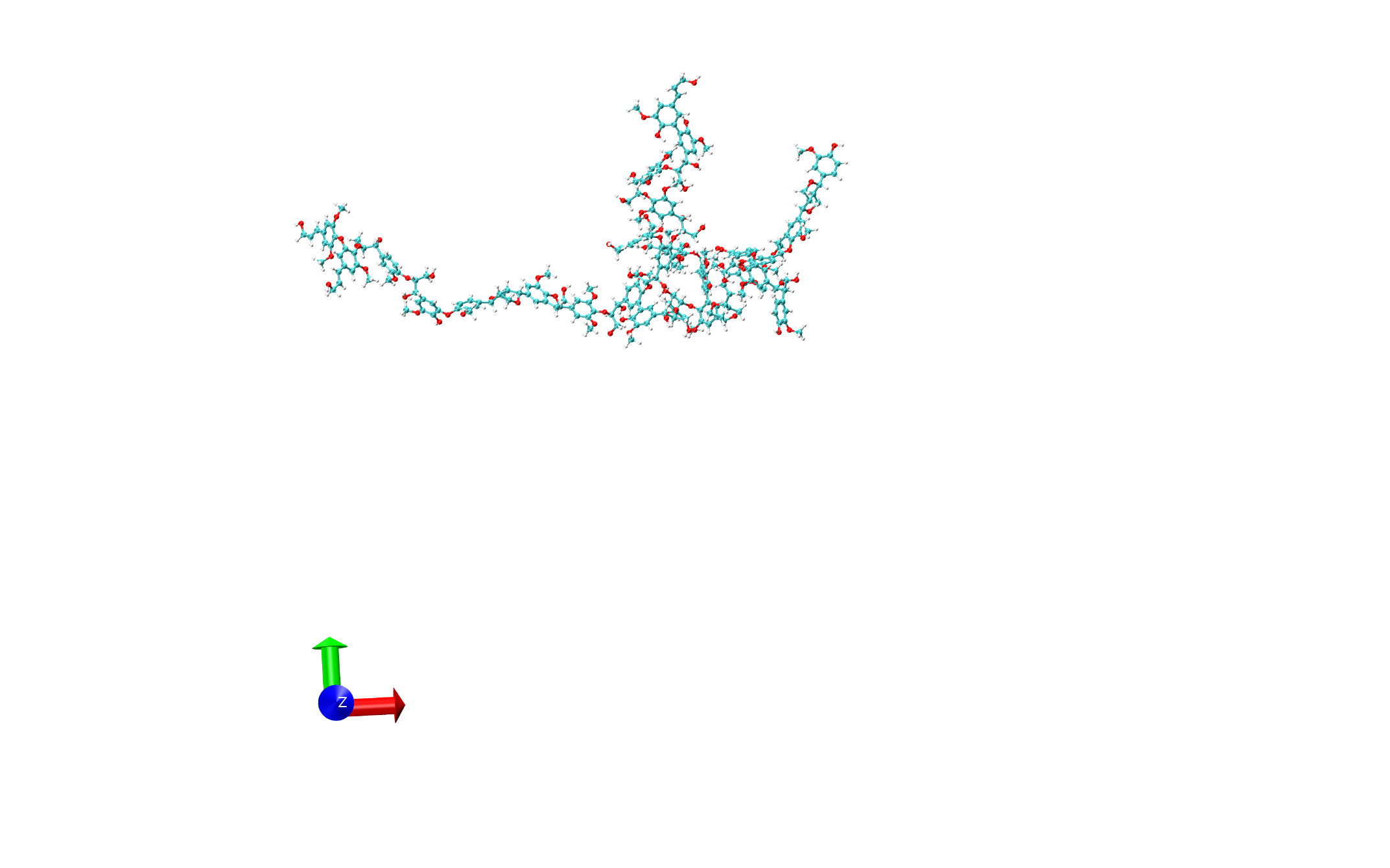   1. [TBA][Gly] |
| --- |
| 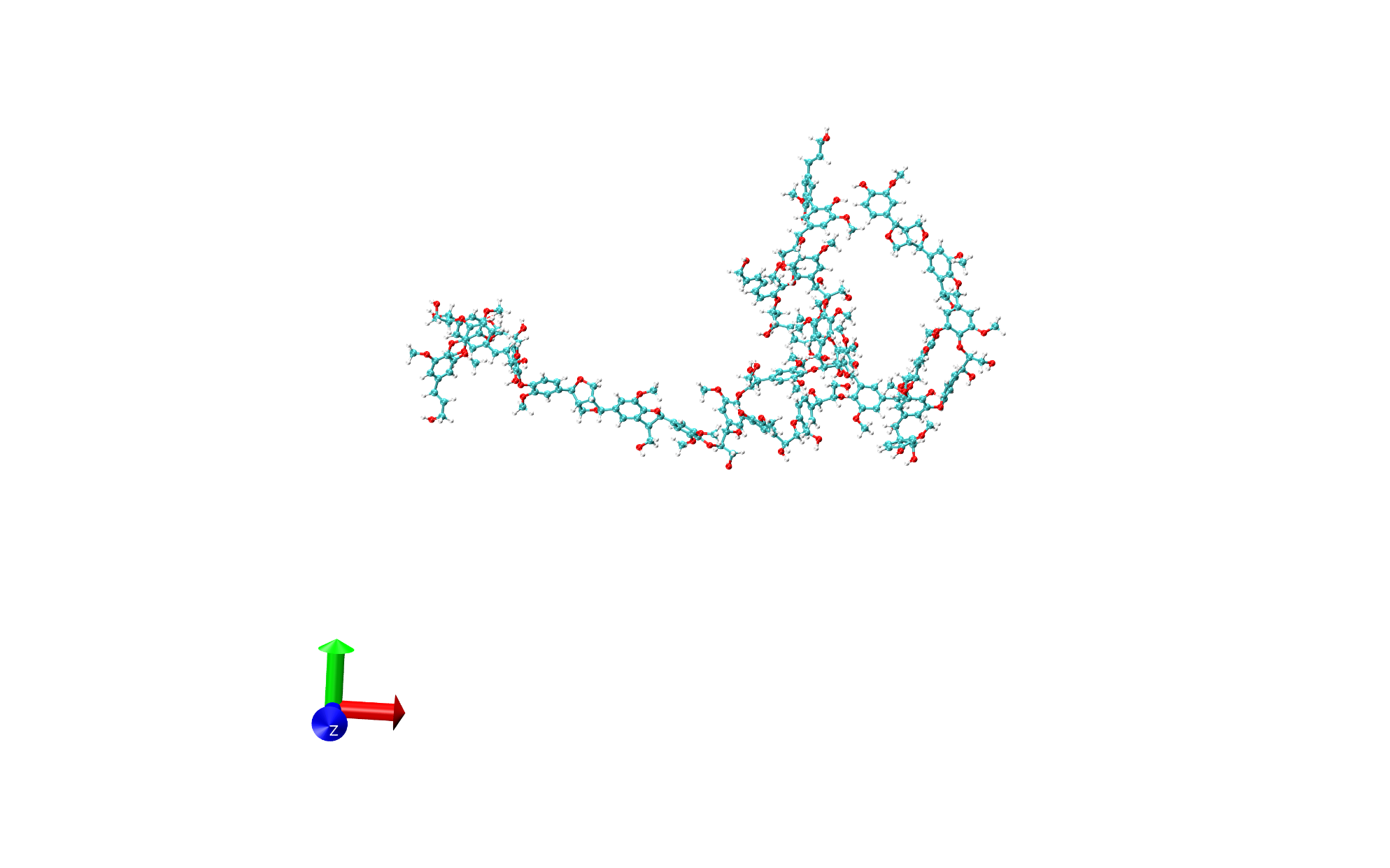   1. [TBA][Ala] |
| 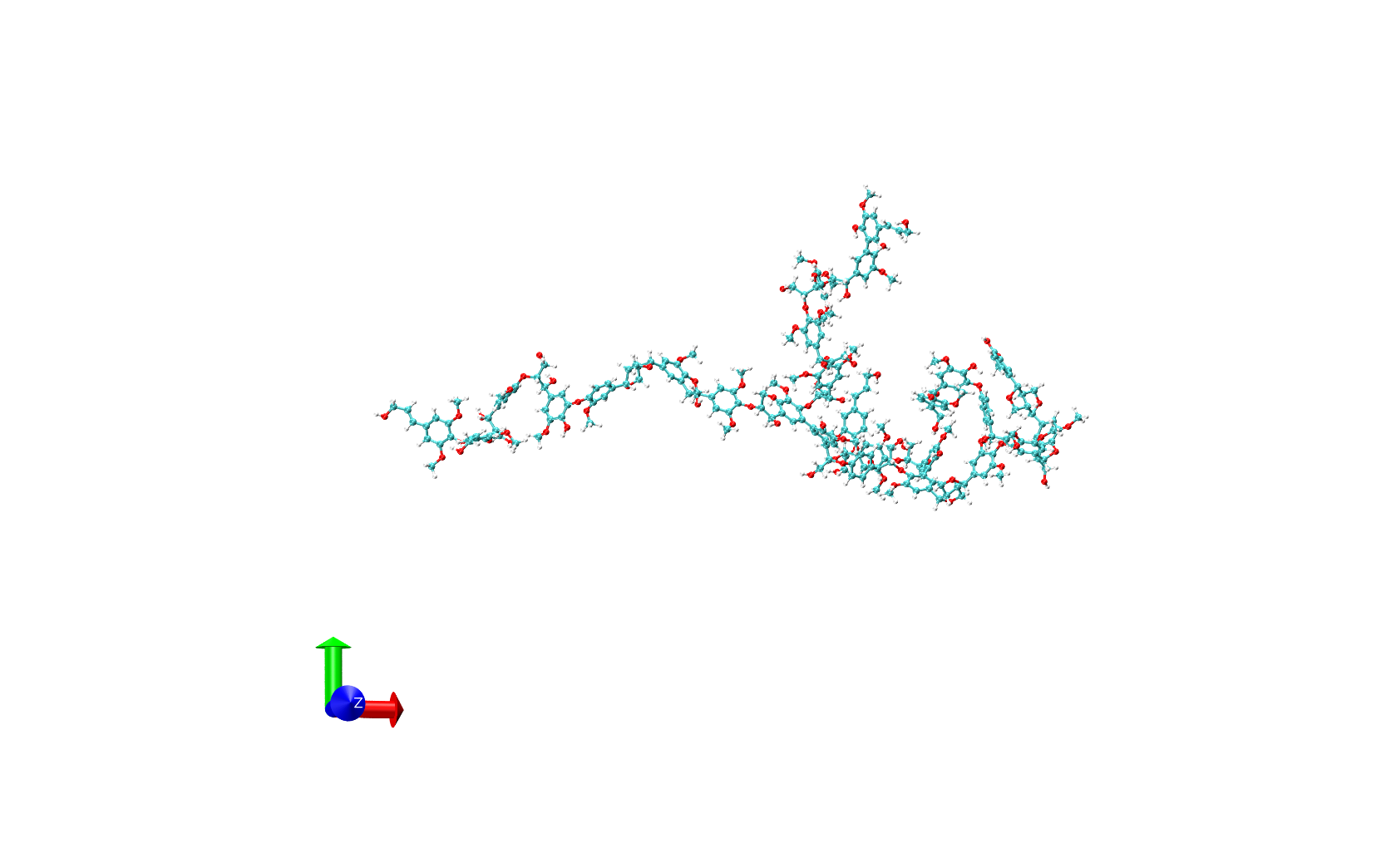   1. [Ch][Lys] |

**Figure S8.** Representative snapshots of the lignin from the MD simulation trajectories in ILs, which has the *R_g_* in Fig. 8. Carbon, oxygen, and hydrogen atoms are colored in cyan, red, and white, respectively.
